# Soft Hydrogen-Bonded Organic Frameworks Constructed Using a Flexible Organic Cage Hinge

**DOI:** 10.1021/jacs.3c09246

**Published:** 2023-10-12

**Authors:** Qiang Zhu, Lei Wei, Chengxi Zhao, Hang Qu, Bowen Liu, Thomas Fellowes, Siyuan Yang, Alexandra Longcake, Michael J. Hall, Michael R. Probert, Yingbo Zhao, Andrew I. Cooper, Marc A. Little

**Affiliations:** † Department of Chemistry and Materials Innovation Factory, University of Liverpool, Liverpool L7 3NY, U.K.; ‡ Leverhulme Research Centre for Functional Materials Design, University of Liverpool, Liverpool L7 3NY, U.K.; § School of Physical Science and Technology, ShanhaiTech University, Shanghai 201210, China; ∥ Key Laboratory for Advanced Materials and Joint International Research Laboratory of Precision Chemistry and Molecular Engineering, Feringa Nobel Prize Scientist Joint Research Center, Frontiers Science Center for Materiobiology and Dynamic Chemistry, Institute of Fine Chemicals, School of Chemistry and Molecular Engineering, East China University of Science and Technology, Shanghai 200237, China; ⊥ Chemistry, School of Natural and Environmental Sciences, Newcastle University, Newcastle upon Tyne NE1 7RU, U.K.

## Abstract

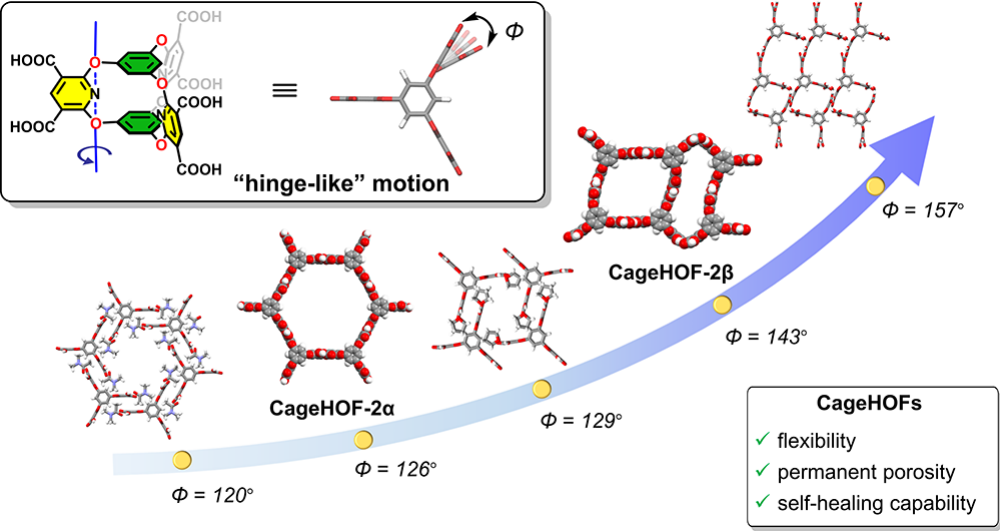

Soft porous crystals combine flexibility and porosity, allowing them to respond structurally to external physical and chemical environments. However, striking the right balance between flexibility and sufficient rigidity for porosity is challenging, particularly for molecular crystals formed by using weak intermolecular interactions. Here, we report a flexible oxygen-bridged prismatic organic cage molecule, **Cage-6-COOH**, which has three pillars that exhibit “hinge-like” rotational motion in the solid state. **Cage-6-COOH** can form a range of hydrogen-bonded organic frameworks (HOFs) where the “hinge” can accommodate a remarkable 67° dihedral angle range between neighboring units. This stems both from flexibility in the noncovalent hydrogen-bonding motifs in the HOFs and the molecular flexibility in the oxygen-linked cage hinge itself. The range of structures for **Cage-6-COOH** includes two topologically complex interpenetrated HOFs, **CageHOF-2α** and **CageHOF-2β**. **CageHOF-2α** is nonporous, while **CageHOF-2β** has permanent porosity and a surface area of 458 m^2^ g^–1^. The flexibility of **Cage-6-COOH** allows this molecule to rapidly transform from a low-crystallinity solid into the two crystalline interpenetrated HOFs, **CageHOF-2α** and **CageHOF-2β**, under mild conditions simply by using acetonitrile or ethanol vapor, respectively. This self-healing behavior was selective, with the **CageHOF-2β** structure exhibiting structural memory behavior.

## Introduction

Soft porous crystals have attracted much interest since they were proposed as third-generation porous framework materials by Kitagawa and co-workers in 1998.^1,2^ In contrast to rigid porous frameworks, soft porous crystals can adopt multistable forms that can switch interchangeably in response to external stimuli, such as guest adsorption, irradiation, or electric fields.^1−4^ By combining framework flexibility and porosity, soft porous crystals present unique opportunities in molecular separation, chemical sensing, and guest adsorption processes.^3−7^

Most soft porous crystals reported in the literature are coordination frameworks,^6,8−11^ such as the metal–organic frameworks (MOFs), MIL-53,^12^ and MIL-88,^13,14^ where the secondary building units and organic ligands can rotate to reshape the pores though “hinge-type” rotation.^15^ While there are now a few examples of MOFs that exhibit this type of structural flexibility, reports of flexible MOFs with switchable porosity are still somewhat rare compared to rigid MOFs.^16^ By contrast, molecular crystals, which do not contain extended coordination bonded frameworks, can exhibit more profound dynamic behavior: for example, to allow the encapsulation of guests such as enzymes.^17^ However, dynamic flexibility frequently results in a permanent loss of porosity in molecular crystals.

**Figure 1:**
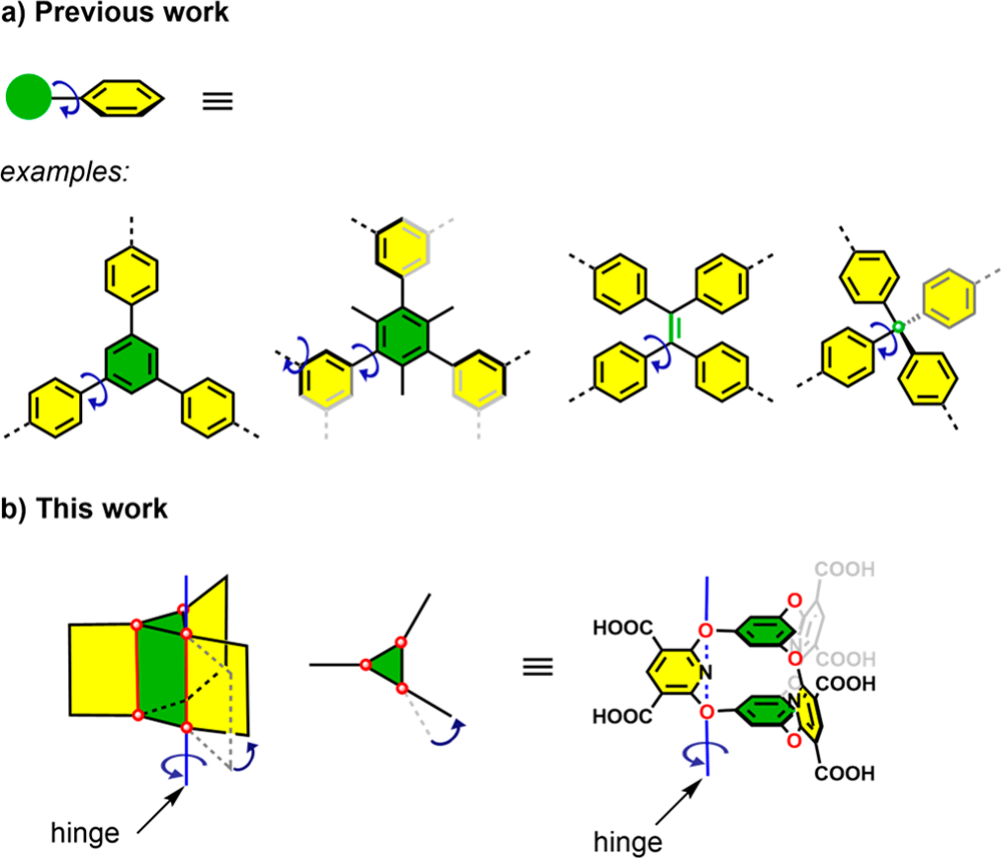
Representative flexible molecules used in the formation of soft HOFs. (a) Most examples involve carbon–carbon single bond-based rotation. (b) Here, we exploit “hinge-like” rotation for cage-based HOF linkers.

Porous organic molecular crystals, such as hydrogen-bonded organic frameworks (HOFs), can experience guest-adsorption-induced structural gate-opening behavior.^18−26^ In most cases, the flexibility of these HOFs can be explained in large part by related rotations around single carbon–carbon bonds (Figure 1a). A different approach is to mimic the “hinge-type” rotation used to tune the pore size in MOFs, which is synthetically tunable^27^ and, to a degree, computationally predictable.^15^ However, translating this approach into the area of HOFs requires new three-dimensional (3-D) building blocks that feature “hinge-like” rotatable groups. Such building blocks are rare, particularly for porous HOFs where building block rigidity has tended to be a prerequisite to prevent HOFs from collapsing into nonporous structures.^28,29^

Here, we prepared soft porous organic crystals using a flexible oxygen-bridged cage molecule (**Cage-6-COOH**, Figure 1b). **Cage-6-COOH** contains six trigonal prismatically arranged carboxylic acid groups and somewhat flexible oxygen bridges that allow the hinge-like rotation of trigonally arranged aromatic pillars. This conformational flexibility endows **Cage-6-COOH** with rich structural behavior in the solid state. We found that **Cage-6-COOH** can crystallize to form both a 3-fold (**CageHOF-2α**) and 2-fold (**CageHOF-2β**) interpenetrated HOF, along with five other solvated HOFs. In combination, this diverse family of **Cage-6-COOH** crystal structures revealed the extensive conformational flexibility of the aromatic pillars and their ability to rotate from 90° degrees in **CageHOF-2β** to 157° degrees in **CageHOF-2·H**_**2**_**O** in a hinge-like rotation. This profound flexibility of **Cage-6-COOH** enabled the soft HOF to self-heal, transforming into crystalline solids upon treatment with organic vapors. Moreover, **CageHOF-2α** switches to **CageHOF-2β** in response to ethanol vapor, which exhibits permanent porosity and has a Brunauer–Emmett–Teller (BET) surface area (*SA*_BET_) of 458 m^2^ g^–1^, as measured using N_2_ adsorption at 77 K.

## Results and Discussion

Triangular prismatic-shaped molecules are a common polyhedral motif for constructing 3-D extended frameworks, including materials with flexible structures^14^ and high storage capacities.^30^ To date, several porous HOFs have been prepared using triangular prismatic building blocks, and three main approaches have been used to control the geometries of the molecular cores (Figure S1).^21,31−39^ One approach uses triptycene-based cores.^31−34,39^ The second approach uses steric hindrance to control the rotation of functional groups appended to the benzene rings.^21,35,36^ Here, we explore the third approach, which uses oxygen-bridged trigonal prismatic organic cages^37,38^ (Figure 1b). An advantage of oxygen-bridged organic cages is their stability and excellent tolerance to a wide range of synthetic conditions and modifications, which has yielded a rich family of organic cages with diverse functions and geometries.^40−46^

We synthesized the carboxylic acid-functionalized cage compound, **Cage-6-COOH**, by hydrolyzing the cyano groups of a previously reported cage, **Cage-6-CN** (Schemes S1–S4).^40^ We confirmed the formation of **Cage-6-COOH** using ^1^H, ^13^C, HSQC (Figures S2–S4), and HMBC spectroscopy (Figure S5), along with MALDI-TOF mass spectroscopy. In the ^1^H NMR spectrum, a singlet corresponding to the proton of the carboxylic acid group was observed at a low field (δ = 13.2 ppm), indicating the successful conversion of **Cage-6-CN** to **Cage-6-COOH** (Figure S2). We also observed two singlets at δ = 8.78 and 6.76 ppm, assigned to the two aromatic protons, indicating that **Cage-6-COOH** adopts a high *D*_*3h*_-symmetric structure in solution.

### Crystallization of **Cage-6-COOH**

**Figure 2:**
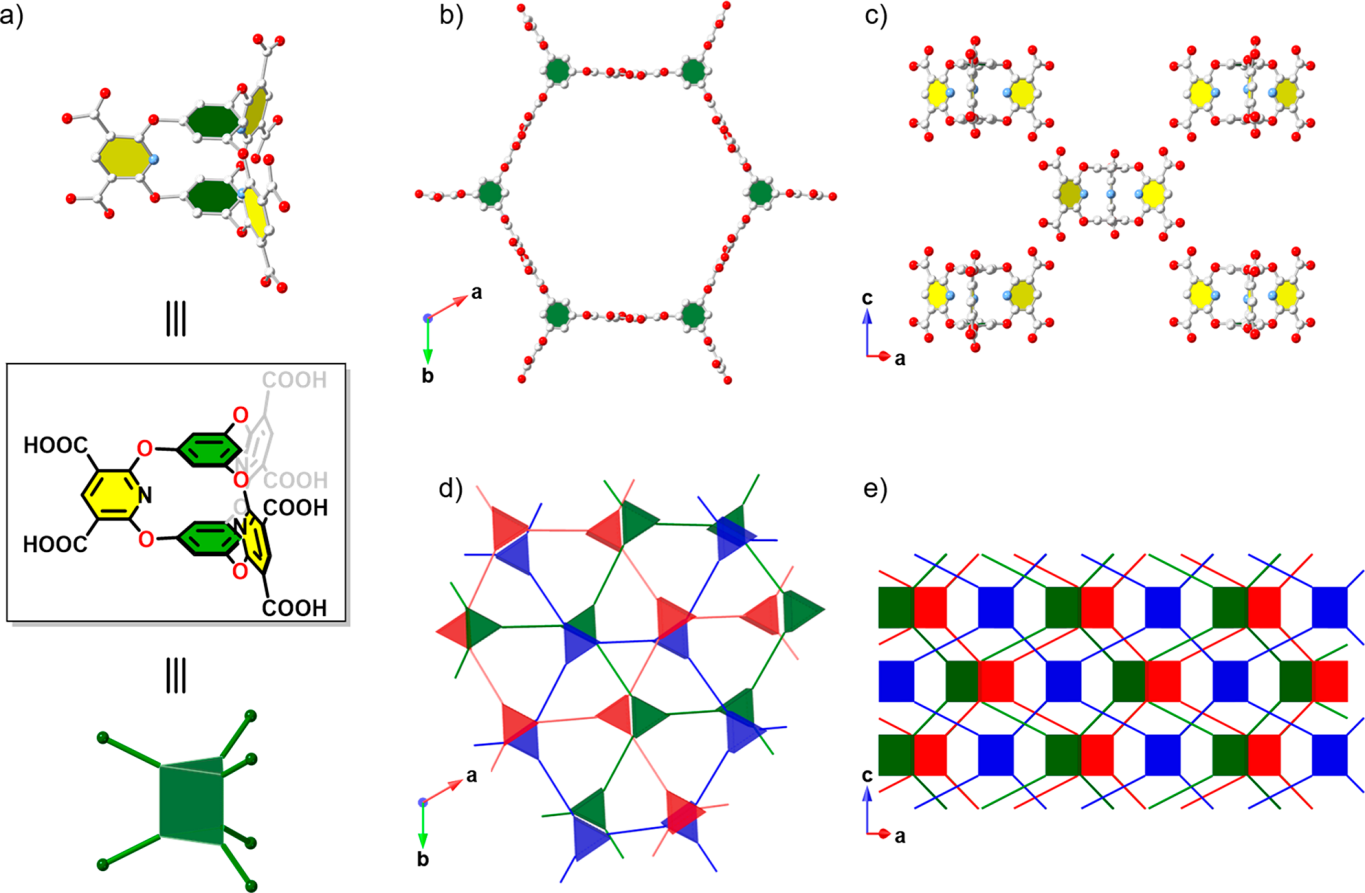
Crystal structures of **CageHOF-2α**. (a) The structure of **Cage-6-COOH** can be represented topologically as a triangular prism. (b) Top view and (c) side view of **CageHOF-2α** under the noninterpenetrated **acs** topology. Atom colors: C, white; N, blue; and O, red. H atoms are omitted for clarity. (d) Top view and (e) side view **CageHOF-2α** under the 3-fold interpenetrated **acs** topology, where the triangular prisms represent **Cage-6-COOH** and the cage nodes belonging to the different nets colored here in green, red, and blue.

We first crystallized **Cage-6-COOH** from tetrahydrofuran (THF) after carefully layering acetonitrile (CH_3_CN) on the THF solution (see the SI, Section 4 for full details). After leaving the crystallization undisturbed at room temperature for 5 days, we observed colorless needle-like crystals that were suitable for X-ray analysis (Figures S6 and S7). Single-crystal X-ray diffraction (SCXRD) analysis revealed that **Cage-6-COOH** crystallized from the THF/CH_3_CN solution in the hexagonal space group *P*6̅2*c* (*a* = 22.6444(8) Å, *c* = 16.4319(5) Å, *V* = 7296.9(6) Å^3^). In the *P*6̅2*c* crystal structure (referred to as **CageHOF-2α**), we found half of the **Cage-6-COOH** molecule in the asymmetric unit and the six carboxylic acid groups in a nearly triangular prismatic arrangement (Figure 2a). In the extended **CageHOF-2α** structure, each **Cage-6-COOH** hydrogen bonds to six neighboring **Cage-6-COOH** molecules via strongly directional carboxylic acid dimers at donor–acceptor distances in the range of 2.58–2.59 Å. This hydrogen-bonding motif yields a 3-D HOF with underlying **acs** network topology (Figure 2b,c). However, due to the large 2.2-nm-sized hexagonal-shaped pores (Figure 2b), three HOF networks with **acs** topologies interpenetrate in **CageHOF-2α** (Figure 2d,e).

We confirmed the bulk-phase purity of **CageHOF-2α** by powder X-ray diffraction (PXRD) using a Pawley refinement of the CH_3_CN-solvated material loaded in a capillary (see Figure S10 for refinement details). The Pawley refinement and the simulated PXRD pattern for **CageHOF-2α** indicate that the CH_3_CN-solvated crystalline sample was phase-pure. **CageHOF-2α** contains three different and modestly sized (<10 Å) triangular-shaped solvent-filled pores (Figure 2d,e), so we next investigated its stability to activation. However, all attempts to remove the solvent from the crystal pores, including heating (Figures S11 and S12) and supercritical CO_2_ drying (scCO_2_, Figure S13), caused **CageHOF-2α** to transform into a low-crystallinity solid (referred to as **CageHOF-2**) that was nonporous based on N_2_ sorption at 77 K (Figure S14). We used variable-temperature (VT) PXRD measurement to monitor the desolvation of the CH_3_CN-solvated **CageHOF-2α** sample. During the VT-PXRD study, we observed a contraction along the *a* = *b* unit cell axes while heating the sample to remove the solvent from the crystal pores. Over the same range, the structure appeared to remain unchanged along the *c* unit cell axis (Figure S15), which we attribute to the denser packing of **Cage-6-COOH** layers in the **CageHOF-2α** crystal structure (Figure S16).

**Figure 3:**
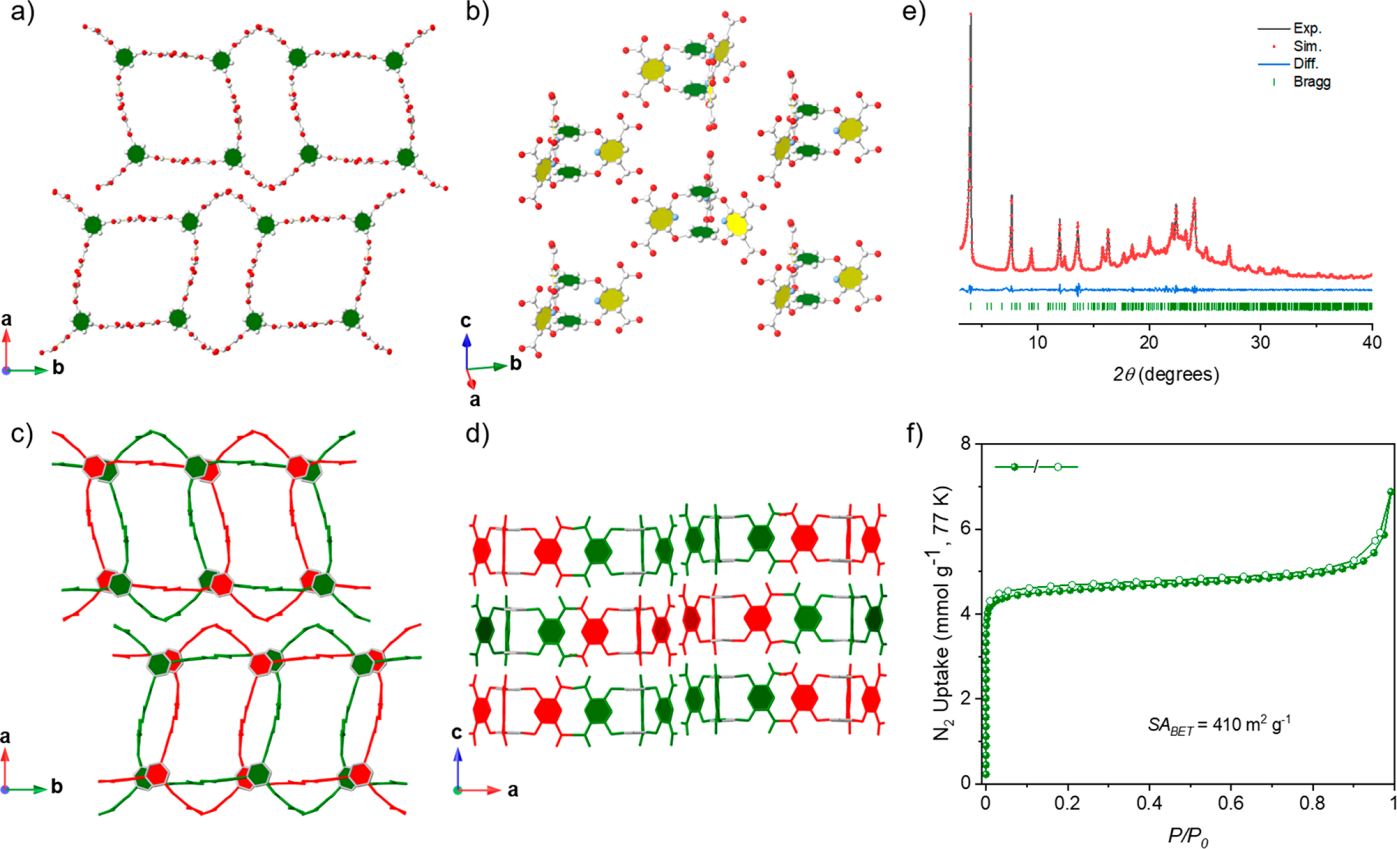
Crystal structures of **CageHOF-2β**. Top view (a) and side view (b) of one hydrogen-bonded network in **CageHOF-2β**, highlighting the distorted **acs** network topology. Atom colors: C, gray; N, blue; O, red; and H, white. Top view (c) and side view (d) of **CageHOF-2β**, highlighting the 2-fold interpenetrated structure. Disordered solvent molecules were omitted for clarity. (e) PXRD pattern fitting of solvated **CageHOF-2β** with Pawley refinement (Cu-Kα, *R*_wp_ = 2.39% and *R*_p_ = 1.68%, *P*2_1_2_1_2, *a* = 44.06 Å, *b* = 11.74 Å, *c* = 16.19 Å, *V* = 8374.5 Å^3^). (f) N_2_ sorption isotherms of **CageHOF-2β** at 77 K.

Next, we expanded our crystallization screen and discovered a new polymorph, **CageHOF-2β**, that was formed by crystallizing **Cage-6-COOH** from ethanol (EtOH) after leaving a solution to evaporate at 343 K. During the crystallization, we observed spindle-like crystals on the surface of the vial (Figure S8). Due to the small crystal size and weak diffraction, SCXRD quality was limited but sufficient to determine the crystal packing of **Cage-6-COOH**. As shown in Figure 3, the **CageHOF-2β** structure, which has orthorhombic *P*2_1_2_1_2 symmetry (*a* = 43.576(4) Å, *b* = 11.7207(9) Å, *c* = 16.2030(8) Å, and *V* = 8275.5(11) Å^3^), contains two pores with different sizes and shapes. In **CageHOF-2β**, each **Cage-6-COOH** molecule in the structure hydrogen bonds with six neighboring **Cage-6-COOH** molecules via carboxylic acid dimers at donor–acceptor distances in the range of 2.51–2.74 Å. However, unlike those in **CageHOF-2α**, the hydrogen-bond motifs in **CageHOF-2β** are not all planar, and the aromatic pillars have profoundly different orientations. For comparison, the dihedral angles in **CageHOF-2α** range between 116° and 126°, while the dihedral angles in **CageHOF-2β** range between 90° and 143°. In addition, there are two interpenetrated HOF networks in the extended **CageHOF-2β** structure compared to three in **CageHOF-2α**. We attribute these differences to the hinge-like rotation of the **Cage-6-COOH** pillars since the change in solvent did not appear to disrupt the hydrogen-bonding interactions between the cage molecules.

We performed a refinement on the PXRD of EtOH-solvated **CageHOF-2β** to confirm its phase purity. (See Figure 3e for the Pawley refinement and Figure S17 for the Rietveld refinement.) The excellent similarity between the experimental PXRD data and the PXRD refinements indicated that the bulk **CageHOF-2β** sample was phase-pure and that it closely matched the single-crystal structure.

### Properties of **CageHOF-2β**

We first investigated the stability of **CageHOF-2β** by *in situ* VT-PXRD analysis (Figures S18 and S19). No significant changes were observed in the VT-PXRD patterns of **CageHOF-2β** while increasing the temperature from 303 to 433 K or upon recooling the sample to room temperature, indicating that **CageHOF-2β** has good thermal stability (Figure S18). From the VT-PXRD study, we also observed that **CageHOF-2β** contracts along the *a* and *b* unit cell axes during heating while expanding along the *c* unit cell axis (Figure S20). We attribute this to the hydrogen-bonding interactions between **Cage-6-COOH** molecules reorganizing into a more planar geometry along the *c* unit cell axis, which enables the **Cage-6-COOH** molecules to pack more closely along the *a* and *b* unit cell axes (Figure S21).

This thermal stability encouraged us to obtain crystalline porous materials by careful desolvation treatment for **CageHOF-2β**. Before studying the porosity of **CageHOF-2β**, we first exchanged the EtOH crystallization solvent with acetone and then *n*-pentane over 2 days, noting that *n*-pentane is the most volatile and least likely to interact strongly with the pore walls (see SI Section 4 for details). Then, we activated the pores under a dynamic vacuum at room temperature. Unlike **CageHOF-2α**, the crystallinity of **CageHOF-2β** appeared to be preserved after activating the crystal, according to the PXRD patterns (Figure 3e and Figure S22). However, the NMR spectrum indicates that the activated **CageHOF-2β** still contained a trace amount of EtOH solvent (3.5 wt %, Figure S23). Thermogravimetric analysis (TGA, Figure S24) showed a 5% weight loss before 450 °C, in broad agreement with the NMR data.

Next, we recorded an N_2_ sorption isotherm for the activated **CageHOF-2β** at 77 K. This gave a type-I sorption isotherm with a sharp uptake over the low-pressure range (*P*/*P*_0_ = 0–0.01), indicating that **CageHOF-2β** possesses micropores (Figure 3f). The *SA*_BET_ also revealed a surface area of 410 m^2^ g^–1^ (Figures S25), which is similar to that of other soft porous HOFs reported in the literature.^22,24,47^ We also recorded a PXRD pattern of **CageHOF-2β** after the N_2_ sorption isotherm, which confirmed the good stability of **CageHOF-2β** during this sorption measurement (Figure S26).

### Hinge-like Flexibility of **Cage-6-COOH**

The crystal structures of **CageHOF-2α** (Figure S27 and Table S4) and **CageHOF-2β** (Figure S28 and Table S5) motivated us to explore further the crystallization behavior of **Cage-6-COOH** and its conformational flexibility. We found five other crystal structures by crystallizing **Cage-6-COOH** from the following solvent mixtures: dimethylacetamide (DMAc)/acetone (**CageHOF-2·DMAc**, Figure S29 and Table S6), diethyl sulfoxide (DESO) (**CageHOF-2·DESO**, Figure S30 and Table S7), *N*-methyl-2-pyrrolidone (NMP) (**CageHOF-2·NMP**, Figure S31 and Table S8), THF/pentane (**CageHOF-2·THF**, Figure S32 and Table S9), and EtOH/H_2_O (**CageHOF-2·H**_**2**_**O**, Figure S33 and Table S10). Notably, the crystal structures from NMP and DESO were discovered using encapsulated nanodroplet crystallization techniques,^48^ which we have used here for the first time to study the crystallization behavior of porous molecular HOFs (see SI Section 4 for details).

**Figure 4:**
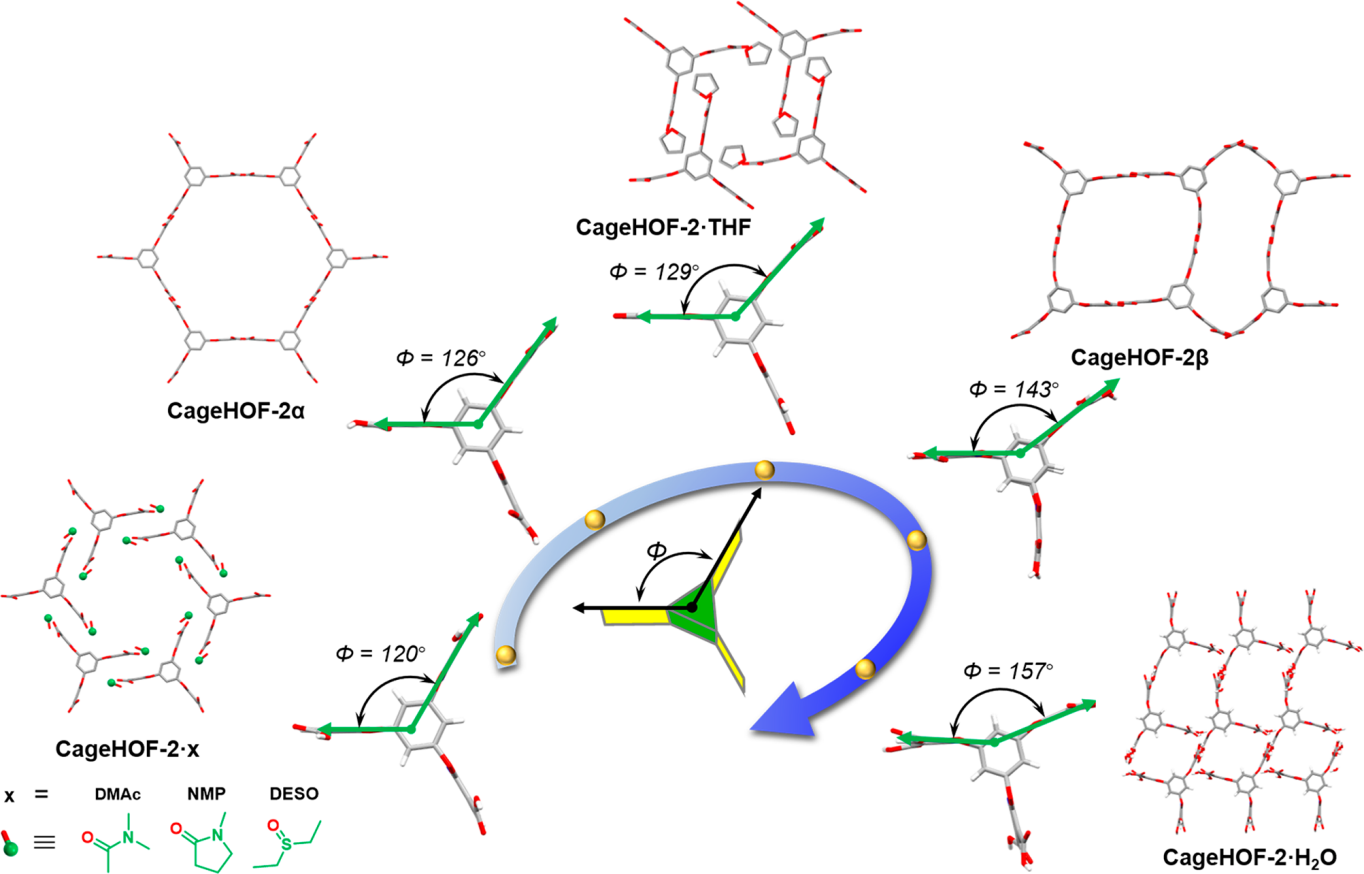
Scheme illustrating the hinge-like motion of **Cage-6-COOH** in various crystal structures. The dihedral angles of the pillar rings change from 90° to 157° in these crystal structures.

The crystal structures of **CageHOF-2·DMAc**, **CageHOF-2·NMP**, and **CageHOF-2·DESO** are isostructural with respect to the crystal packing of **Cage-6-COOH**, despite the differences between the physical properties of the crystallization solvents (Figure S34). In total, we measured seven SCXRD structures of **Cage-6-COOH** during this study. In combination, these structures exemplify the profound hinge-like flexibility of the oxygen bridges and hydrogen-bonded interactions (Figure 4, Figures S35–S37, and Tables S11–S13) and the apparent rigidity of the aligned 1,3,5-substituted aromatic rings in the cage core (Table S14). Indeed, we found that the dihedral angle (Φ in Figure 4) could rotate from 90° in **CageHOF-2β** to up to 157° in **CageHOF-2·H**_**2**_**O**. Furthermore, the three aromatic pillars in **Cage-6-COOH** appear to be independently flexible and typically have different conformations in each structure, highlighting the soft nature of these frameworks (Table S11). We used relaxed scan density functional theory (DFT) calculations to investigate the flexibility of **Cage-6-COOH**. These DFT calculations revealed that the lowest-energy conformer had dihedral angles of around 120°. However, the energy required to rotate the pillars of **Cage-6-COOH** by 60° from 120° was approximately 6.4 kcal mol^–1^ (Figure S38 and Section 1.2.7 for full details), which is within the usual energetic range of polymorphism.^49^ Here, this hinge-like rotation in **Cage-6-COOH** adapts to accommodate guests in the resulting crystals. It is therefore comparable to prototypical MOFs such as MIL-53, which has pores that can expand and contract using a “hinge-type” rotation,^4,6^ albeit through a different process.

Furthermore, we performed molecular dynamics (MD) simulations to investigate the flexibility of **Cage-6-COOH**. We performed these MD simulations using the RASPA software package and a 2 × 2 × 3 supercell generated using the SCXRD of **CageHOF-2α** (see Section 1.2.7 for details and Figure S39). These MD simulations indicate that **Cage-6-COOH** molecules exhibit “hinge-like” rotational motion in **CageHOF-2α** (Supporting Information Video 1). At the same time, the packing of the **Cage-6-COOH** layers in the structure appears to be rigid (Supporting Information Video 2). This observation agrees well with the VT-PXRD measurements performed using the CH_3_CN-solvated **CageHOF-2α** (Figures S11, S12, and S15), which indicated that the packing of **Cage-6-COOH** layers along the *c* unit cell axis remained essentially unchanged during the VT-PXRD measurement. We attribute the rigidity of hydrogen-bonded layers of **Cage-6-COOH** in **CageHOF-2α** to the order we observed in the desolvated PXRD patterns (Figure S13).

### Self-Healing Properties of **Cage-6-COOH**

**Figure 5:**
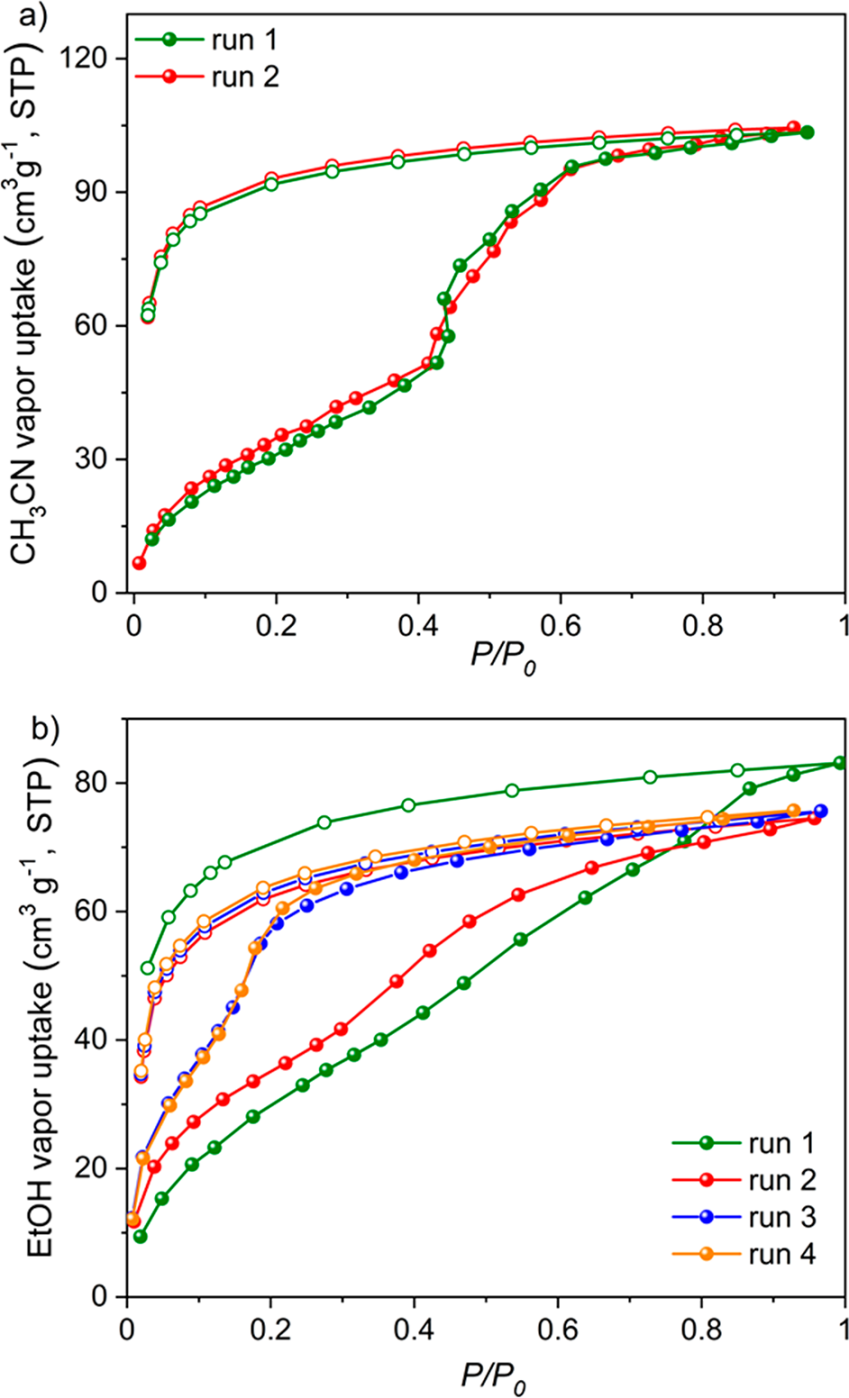
Organic vapor sorption isotherms of activated **Cage-HOF-2** at 298 K. (a) CH_3_CN, first cycle: green circles; second cycle: red circles. (b) EtOH, first cycle: green circles; second cycle: red circles; third cycle: blue circles; fourth cycle: orange circles. Adsorption curves shown as filled symbols, desorption curves shown as unfilled symbols.

Although HOFs can recover their crystallinity via recrystallization from solution, HOFs that self-heal via solid-state structural transformations are rare.^18,20,47^ The profound flexibility of **Cage-6-COOH** motivated us to study its self-healing properties. To do this, we conducted organic vapor sorption experiments by exposing an activated, solvent-free, activated **CageHOF-2** powder to CH_3_CN vapor. In the CH_3_CN sorption isotherm, we observed a stepwise increase in the uptake (Figure 5a) with a gradual increase over the low-pressure range, followed by a steep increase in the uptake at *P*/*P*_0_ = 0.43. The pores then appeared to saturate by about *P*/*P*_0_ = 0.85. From the CH_3_CN isotherm, we calculated that the experimental pore volumes of the activated **CageHOF-2** powder were 0.12 cm^3^ g^–1^ at *P*/*P*_0_ = 0.41, 0.23 cm^3^ g^–1^ at *P*/*P*_0_ = 0.68, and 0.25 cm^3^ g^–1^ at *P*/*P*_0_ = 0.93. These pore volumes are below the theoretical uptake of **CageHOF-2α** of 0.40 cm^3^ g^–1^ calculated using a 2.5 Å probe; however, this can be rationalized by the poor structural stability of activated **CageHOF-2α**. PXRD patterns recorded after CH_3_CN vapor exposure indicated that the **CageHOF-2** powder had switched to **CageHOF-2α** with high crystallinity. Further PXRD experiments suggested that this transformation was reversible; that is, the regenerated **CageHOF-2α** crystal transformed to **CageHOF-2** powder again after removing the CH_3_CN (Figures S40–S42). We also observed this type of gate open behavior during a CO_2_ adsorption isotherm recorded at 195 K (Figure S43), with the pore volume increasing from 0.024 cm^3^ g^–1^ at *P*/*P*_0_ = 0.21 to 0.185 cm^3^ g^–1^ at *P*/*P*_0_ = 0.96.

After the CH_3_CN vapor sorption isotherm, we used the same sample for an EtOH vapor sorption isotherm after degassing the material at 333 K for 12 h. Again, we observed a gate-opening-type sorption isotherm, with a sharp increase occurring from about *P*/*P*_0_ = 0.35, indicating the existence of a phase transformation (Figure 5b). In this case, the sorption process was irreversible until the fourth cycle. After that, the saturation point gradually moved to the low-pressure range, suggesting that the material transforms into a porous metastable phase step by step over successive adsorption experiments, as verified by PXRD analysis (Figure S44). From the EtOH isotherm, we calculated that the experimental pore volume of the **CageHOF-2** powder was 0.197 cm^3^ g^–1^ at *P*/*P*_0_ = 0.97, which is close to the theoretical value of **CageHOF-2β** of 0.22 cm^3^ g^–1^ calculated using a 2.5 Å probe. A further PXRD study confirmed that activated **CageHOF-2** powder transformed into the porous **CageHOF-2β** phase upon treatment with EtOH vapor (Figure S41). Remarkably, this structural transformation appeared to be complete after 5 min under saturated EtOH vapor at room temperature, as indicated by a dynamic PXRD study (Figure S45). This switching behavior is advantageous for the scale-up preparation of **CageHOF-2β** with improved porosity (458 m^2^ g^–1^, Figures S46 and S47). We propose that the fast crystalline transformation is due to the synergistic effect of the flexible oxygen bridges in **Cage-6-COOH** and flexible carboxylic acid hydrogen-bond dimers. Notably, we could not transform **CageHOF-2β** into **CageHOF-2α** after exposing **CageHOF-2β** to CH_3_CN vapor, suggesting that activated **CageHOF-2** powder exhibits a structural “memory” behavior, as observed in some soft porous MOF materials.^2,50^

We screened the self-healing behavior of activated **CageHOF-2** powder in response to different organic solvents, which suggests that the self-healing behavior strongly correlates with the type and functionality of the solvent (Figure S48). For example, we found that a trace amount of THF liquid cleanly transformed activated **CageHOF-2** powder into highly crystalline **CageHOF-2·THF** (Figures S49–S51). By contrast, MeOH transformed activated **CageHOF-2** powder into **CageHOF-2β**, whereas solvents without hydrogen-bond donor and acceptor atoms, such as hexane and toluene, had no apparent effect on the structure of the material (Figure S48). In addition, we found that after being immersed in water for several days, **CageHOF-2α** transformed to **CageHOF-2·H**_**2**_**O**. Interestingly, **CageHOF-2·THF** and **CageHOF-2·H**_**2**_**O** could then be transformed to **CageHOF-2β** using EtOH (Figures S52–S54).

## Conclusions

We have prepared a soft porous crystal using a flexible cage molecule, **Cage-6-COOH**. This cage molecule is decorated with six carboxylic acid groups, and it features a 3-D arrangement of rotationally flexible oxygen bridges that allow it to adapt its conformation in the solid state via hinge-like rotational motion. We found seven crystal structures of **Cage-6-COOH**, including topologically complex 3-D HOFs featuring 3-fold (**CageHOF-2α**) and 2-fold (**CageHOF-2β**) interpenetration. Of these HOFs, **CageHOF-2β** alone had permanent solid-state porosity and an *SA*_BET_ of 458 m^2^ g^–1^. **CageHOF-2α** collapsed into the low-crystallinity **CageHOF-2** powder during activation, as do many other interpenetrated HOFs, except for a few examples.^31,32,51^ However, the hinge-like flexibility of **Cage-6-COOH** enabled the **CageHOF-2** powder to self-heal to recover its **CageHOF-2α** structure using solvent vapor or to transform selectively into porous **CageHOF-2β**. These structural transformations were fast and complete within 5 min, with the crystalline solids exhibiting structural memory behavior during vapor sorption experiments.

Taken together, these results outline a new strategy to generate soft, crystalline, and porous HOFs using organic cage molecules. These soft porous crystalline HOFs mimic the structural behavior of certain MOFs, without the requirement of a coordination network. This lack of a coordination network seems to help **Cage-6-COOH** to rapidly self-heal and undergo rapid and profound structural transformations, even under mild conditions (5 min, solvent vapor, room temperature). This suggests a possible unique advantage of soft porous molecular systems over bonded framework materials. Further exploration of this approach, for example, by constructing HOFs with the molecular hinge motifs found in molecular machines,^52^ might help HOFs to mimic even more complex structural behavior found in nature that is also underpinned by “hinge-type” rotation.^52^

## Supporting Information Available

Figures S1–S54, Tables S1–S14, and Schemes S1–S4; synthetic procedures and methods, NMR, PXRD, SCXRD, ENaCt, TGA, and gas sorption analysis (PDF)
Supplementary Video 1 showing the **CageHOF-2α** MD simulation along the crystallographic *a* axis (MPG)
Supplementary Video 2 showing the **CageHOF-2α** MD simulation along the crystallographic *c* axis (MPG)

## Supplementary Material

ja3c09246_si_001.pdf

ja3c09246_si_002.mpg

ja3c09246_si_003.mpg
