# Supplementary material for: Soft Hydrogen-Bonded Organic Frameworks Constructed Using a Flexible Organic Cage Hinge
Source: J Am Chem Soc. 2023 Oct 12;145(42):23352–60. doi: 10.1021/jacs.3c09246 (PMC10603795; doi:10.1021/jacs.3c09246)
Supplement: Supplementary file 1 — ja3c09246_si_001.pdf [file ja3c09246_si_001.pdf]

Supporting Information for
Soft Hydrogen-Bonded Organic Frameworks Constructed Using a Flexible Organic Cage Hinge

Qiang Zhu,[1,2] Lei Wei,[3] Chengxi Zhao,[1,4] Hang Qu,[1] Bowen Liu,[1] Thomas Fellowes,[1,2] Siyuan Yang,[1] Alexandra Longcake,[5] Michael J. Hall,[5] Michael R. Probert,[5] Yingbo Zhao,[3] Andrew I. Cooper,[1,2*] Marc A. Little[1*]

[1] Materials Innovation Factory and Department of Chemistry, University of Liverpool, 51 Oxford Street, Liverpool L7 3NY, UK.

[2] Leverhulme Research Centre for Functional Materials Design, University of Liverpool, 51 Oxford Street, Liverpool L7 3NY, UK.

[3] School of Physical Science and Technology, ShanghaiTech University, Shanghai 201210, China.

[4] Key Laboratory for Advanced Materials and Joint International Research Laboratory of Precision Chemistry and Molecular Engineering, Feringa Nobel Prize Scientist Joint Research Center, Frontiers Science Center for Materiobiology and Dynamic Chemistry, Institute of Fine Chemicals, School of Chemistry and Molecular Engineering East China University of Science and Technology 130 Meilong Road, Shanghai, 200237, China.

[5] Chemistry, School of Natural and Environmental Sciences, Newcastle University, Newcastle upon Tyne NE1 7RU, UK

Section 1 Materials and methods 2

Section 2 Summary of triangular-prism-shaped molecules used in HOFs 5

Section 3 Synthetic procedures 6

Section 4 Crystallization of Cage-6-COOH 11

Section 5 Characterization of CageHOF-2α 17

Section 6 Characterization of CageHOF-2β 23

Section 7 Structural information 33

Section 8 Structural flexibility investigation 55

References 67

Section 1 Materials and methods

1.1 Materials

All reagents were obtained from Sigma-Aldrich, TCI Europe, Fisher, and Alfa Aesar and used as received. Anhydrous solvents were purchased from Acros Organics and used without further purification. All gases for sorption analysis were supplied by BOC at a purity of ≥99.9 %. Reactions were carried out under a nitrogen atmosphere using standard Schlenk techniques.

1.2 Methods

1.2.1 NMR

NMR spectra were recorded on a Bruker 400 NMR spectrometer at 400 MHz (^{1}H) and 100 MHz (^{13}C) and referenced against the residual ^{1}H or ^{13}C signal of the solvent.

1.2.2 Powder X-ray diffraction (PXRD)

Laboratory powder X-ray diffraction (PXRD) data patterns were collected in transmission mode on samples held on thin Mylar film in aluminium well plates on a Panalytical Empyrean diffractometer equipped with a high throughput screening (HTS) XYZ stage, X-ray focusing mirror, and PIXcel detector, using Cu-Kα radiation. For HT screening, PXRD patterns were measured over the 2θ range 2–40° in 0.013° steps over 10 minutes. Capillary PXRD patterns were collected on powdered samples loaded in borosilicate glass capillaries that were spun to improve averaging. Capillary PXRD patterns were collected on powdered samples loaded in borosilicate glass capillaries, and the capillaries were spun to improve averaging. For the variable temperature experiments, the capillaries were then heated from room temperature to 180 °C using an Oxford Cryosystems 700 series cryostream. An equilibration time of 1 hour was used at each temperature set point.

1.2.3 Single crystal X-ray diffraction (SCXRD)

SCXRD data for **CageHOF-2α**, **CageHOF-2β**, **CageHOF-2·DMAc**, **CageHOF-2·THF**, **CageHOF-2·H$_2$O**, was measured on a Rigaku MicroMax-007 HF rotating anode diffractometer (Mo-Kα radiation, λ = 0.71073 Å, Kappa 4-circle goniometer, Hybrid Pixel Array Detector), and data reduction was performed using CrysAlisPro. SCXRD data for **CageHOF-2·DESO** and **CageHOF-2·NMP** were collected on a

Rigaku XtaLAB Synergy PhotonJet micro-focus sealed X-ray tube diffractometer (Cu-Kα radiation, λ = 1.54184 Å, 4-circle goniometer, HyPix Arc-100 Detector). Structures were solved with SHELXT[1] and refined by full-matrix least-squares on $|F|^2$ by SHELXL[2], interfaced through the program OLEX2[3]. The hydrogen atoms on heteroatoms in **CageHOF-2·DESO** were located using the electron density difference map and refined with their Uiso values fixed at 1.5 times that of the Ueq value of the respective parent atom. All non-hydrogen atoms were refined anisotropically, and all hydrogen atoms were fixed in geometrically calculated positions and refined using the a riding model. For full refinement details, see Tables S4-10.

1.2.4 Gas sorption Analysis

Surface areas were measured by nitrogen sorption at 77.3 K. Powder samples were degassed offline, followed by degassing on the analysis port under vacuum at 25 °C for 15 hours. Isotherm measurements were performed using a Micromeritics ASAP 2020 characterization analyzer. CO_2 sorption isotherms were measured at 195 K using an IGA gravimetric adsorption apparatus (Hiden Isochema, Warrington, UK) fitted with an anti-condensation system, which was carried out in an ultrahigh vacuum system equipped with a diaphragm and turbopumps.

The organic vapor adsorption isotherms were collected using the MicrotracBELSorp-Aqua3 adsorption apparatus with a water circulator bath. Anhydrous solvents were degassed at least five times before being used for vapor adsorption. The sample was degassed at 25 °C for 16 h before and between each cycle of CH_3CN vapor sorption. Afterwards, the sample was degassed at 60 °C for 16 h and then applied for EtOH vapor sorption. The sample was degassed at 25 °C for 5 h between each cycle of EtOH vapor sorption.

1.2.5 Thermogravimetric analysis (TGA)

TGA analysis was carried out using a Netzsch TG 209 F1 Libra instrument using an Al_2O_3 crucible. The sample was heated at a rate of 20 °C/min from 25 °C to 900 °C under a dry nitrogen gas flow.

1.2.6 Optical microscopy images

Optical microscopy images for **CageHOF-2·DESO** and **CageHOF-2·NMP** were recorded using an Olympus SZX16 Microscope with 10X objective lenses and a Sony GXCAM-U3PRO-12 digital colour camera. All other optical microscopy images were recorded using an Olympus BX53 Microscope with 10X objective lenses and an Olympus DP26 digital colour camera under a reflection model.

1.2.7 Computational methods

The relaxed scan DFT calculations were performed with the Gaussian 16 suite of programs.[4] Hybrid M06-2X functional[5] was used during calculation and def2-SVP basis set[6,7] was applied for all atoms. VMD program[8] was used for visualization. The results from the DFT calculations are plotted in Figure S38.

The molecular dynamic (MD) simulations of **CageHOF-2α** were carried out using RASPA software package (version 2.0.47)[9]. For these MD simulations, we generated a 2 × 2 × 3 supercell from the SCXRD structure and used the experimental unit cell dimensions ($a = b = 45.288$ Å, $c = 49.296$ Å, $\alpha = \beta = 90°$, $\gamma = 120°$) as starting values. We performed the MD simulations using the OPLS All-Atom force field[10–12] and applied a potential cutoff of 12 Å. Electrostatic interactions were calculated using the partial charges from the FF. An NPT ensemble (constant number of moles, pressure and temperature) at 1 atm and 298 K was used with the Hoover barostat and thermostat.[13] A timestep of 0.5 fs was used for 0.65 ns in total. From these MD simulations, we found that the system energy was conserved over 100,000 steps (Figure S39). The results from the MD simulations are shown in Supplementary Video 1 (shown along the crystallographic *a* axis) and Supplementary Video 2 (shown along the crystallographic *c* axis)

Section 2 Summary of triangular-prism-shaped molecules used in HOFs

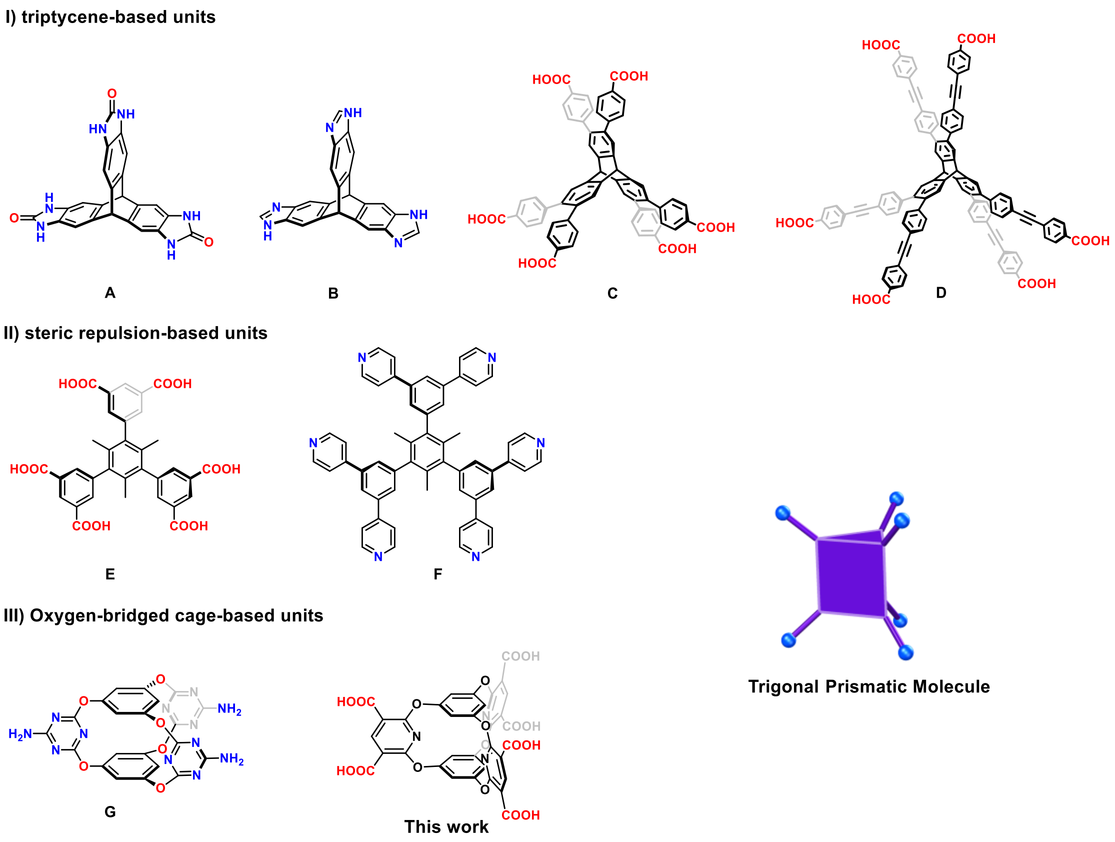

Figure S1 Representative triangular-prism-shaped molecules used in the construction of HOFs: A,[14,15] B,[16] C,[17] D,[18] E,[19,20] F,[21] G.[22,23]

Section 3 Synthetic procedure

Synthesis of 2-amino-6-chloropyridine-3,5-dicarbonitrile[24]

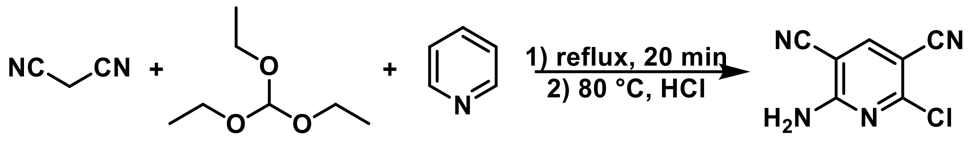

Scheme S1 Synthetic route for 2-amino-6-chloropyridine-3,5-dicarbonitrile.

A mixture of malononitrile (10 g, 151.38 mmol), triethyl orthoformate (12.6 ml, 75.68 mmol) and pyridine (6 ml, 75.68 mmol) was allowed to reflux for 20 minutes, then concentrated HCl (120 ml) was added at 80 °C. The mixture was stirred at 80 °C for 1 h and cooled to room temperature, and water (20 ml) was added. The formed precipitate that was formed was collected by filtration and washed successively with water, ethanol to afford a brown solid (6.3 g, 35.2 mmol, 46%).

^{1}H NMR (400 MHz, DMSO-d_6): δ 8.46 (s, 1H); ^{13}C NMR (100 MHz, DMSO-d_6): δ 160.6, 155.3, 149.5, 115.6, 115.03, 95.6, 89.7.

Synthesis of 2,6-dichloropyridine-3,5-dicarbonitrile (DCDCPy)[24]

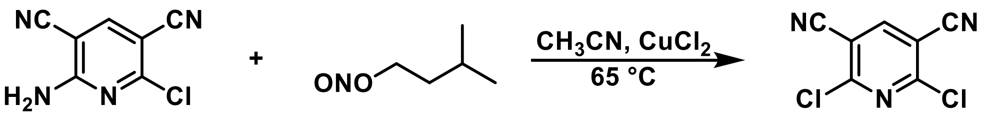

Scheme S2 Synthetic route for DCDCPy.

To a solution of 2-amino-6-chloropyridine-3,5-dicarbonitrile (3 g, 16.8 mmol) and $CuCl_2$ (3.39 g, 25.2 mmol) in dry CH_3CN (150 ml), isopentyl nitrite was added (2.95 g, 25.2 mmol). The mixture was heated at 65°C for 5 h. The solution was acidified (HCl, 2 N) to pH=3, extracted with CH_2Cl_2 (3 × 50 ml) and dried with Na_2SO_4. The solvent was removed under reduced pressure, and the crude product was purified by flash chromatography using petroleum ether/CH_2Cl_2 = 2:1 as eluent to yield 2,6-dichloropyridine-3,5-dicarbonitrile as a white solid (2.5 g, 76%).

^{1}H NMR (400 MHz, $CDCl_3$): δ 8.28 (s, 1H); ^{13}C NMR (100 MHz, $CDCl_3$): δ 155.7, 146.9, 112.2, 110.3.

Synthesis of Cage-6-CN[25]

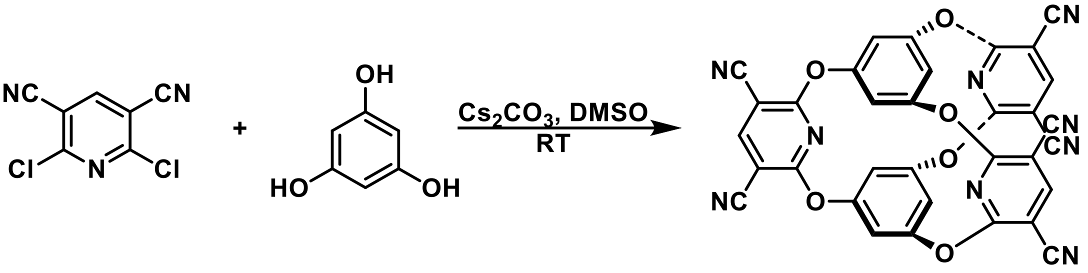

Scheme S3 Synthetic route for Cage-6-CN.

Cage-6-CN was synthesized according to a previously reported method.[25] Under an ambient atmosphere, phloroglucinol (200 mg, 1.59 mmol), 2,6-dichloropyridine-3,5-dicarbonitrile (472 mg, 2.38 mmol), and anhydrous Cs_2CO_3 (1.55 g, 4.76 mmol) were combined. Anhydrous DMSO (25 mL) was added and the reaction was stirred vigorously for 1 h. Then the reaction mixture was extracted with EtOAc after adding water to the reaction system. The combined organics were washed with brine (100 mL), dried over anhydrous Na_2SO_4, filtered, and concentrated in vacuo. The crude product was further purified by silica (DCM: EtOAc = 0–20%), yielding a high-purity white product (267 mg, 0.42 mmol, 53%).

^{1}H NMR (400 MHz, DMSO-d_6): δ 9.08 (s, 3H), 7.10 (s, 6H); ^{13}C NMR (100 MHz, DMSO-d_6): δ 165.2, 153.2, 152.0, 116.2, 113.8, 90.7.

Synthesis of Cage-6-COOH

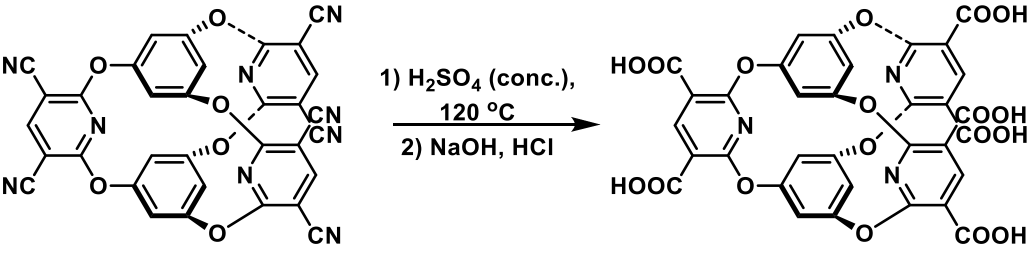

Scheme S4 Synthetic route for Cage-6-COOH.

The mixture of Cage-6-CN (200 mg, 0.32 mmol) and concentrated H_2SO_4 (6 ml) was stirred at 80 °C for 1 h. Then, H_2O (6 ml) was added to the mixture and stirred at 120 °C for 12 h. After cooling to room temperature, 1 M NaOH was added to it until pH > 9

and stirred for another 4 h. 1 M HCl was added to the system and the solid was filtrated and washed with H_2O and acetone, yielding white products (128 mg, 64%).

^{1}H NMR (400 MHz, DMSO-d_6): δ 13.19 (s, 6H_c), 8.78 (s, 3H_a), 6.75 (s, 6H_b); ^{13}C NMR (100 MHz, DMSO-d_6): δ 164.5, 162.9, 154.4, 148.8, 115.2, 108.2. Matrix assisted laser desorption ionization time of flight (MALDI-TOF) mass spectrometry $[M+H]^+$, $[C_{33}H_{15}N_3O_{18}+H]^+$: Calc. 742.04, Found 742.11.

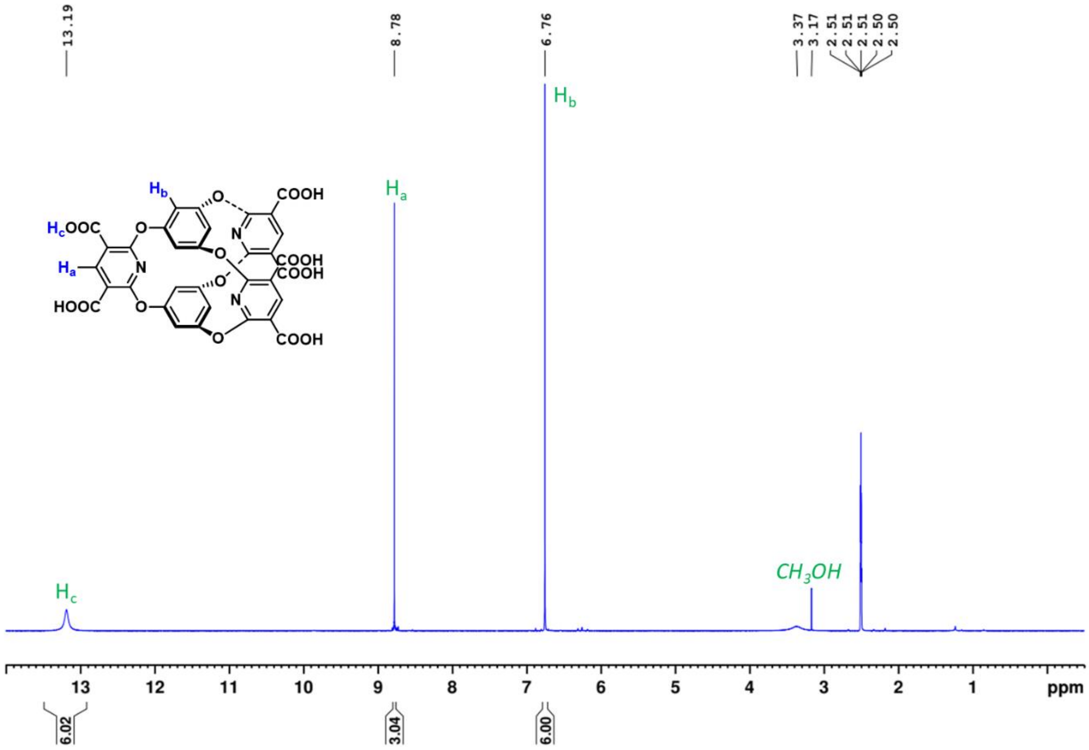

Figure S2 ^{1}H NMR (400 MHz, DMSO-d_6) spectrum of **Cage-6-COOH**.

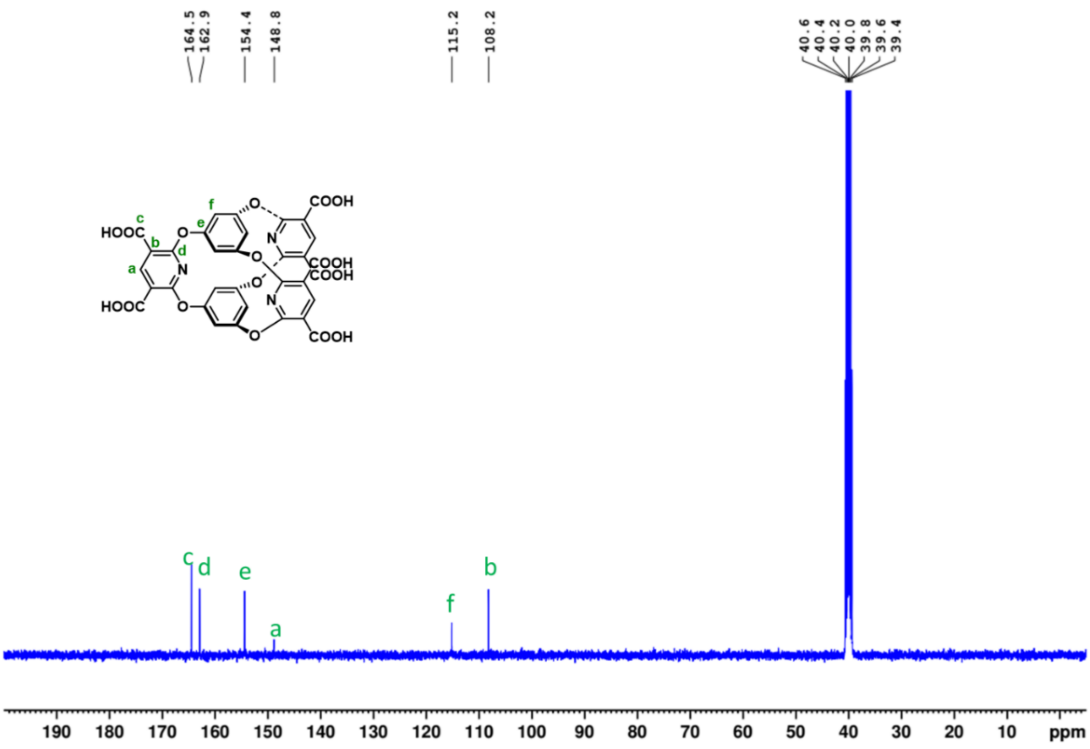

Figure S3 ^{13}C NMR (100 MHz, DMSO-d_6) spectrum of **Cage-6-COOH**.

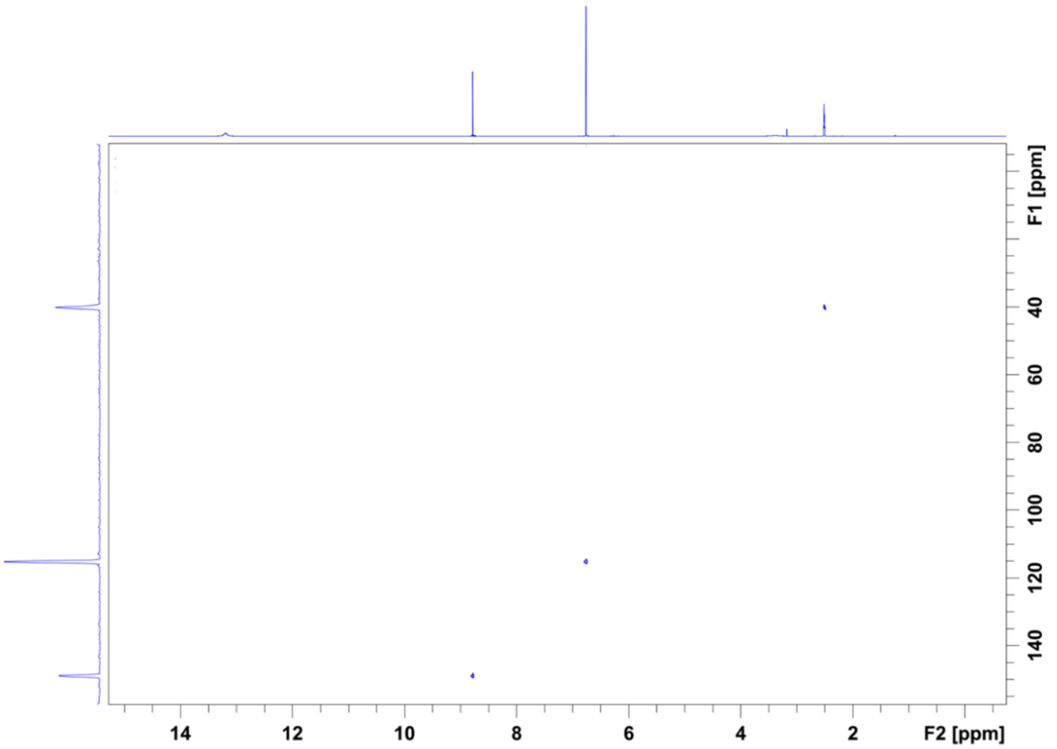

Figure S4 HSQC (DMSO-d_6) spectrum of **Cage-6-COOH**.

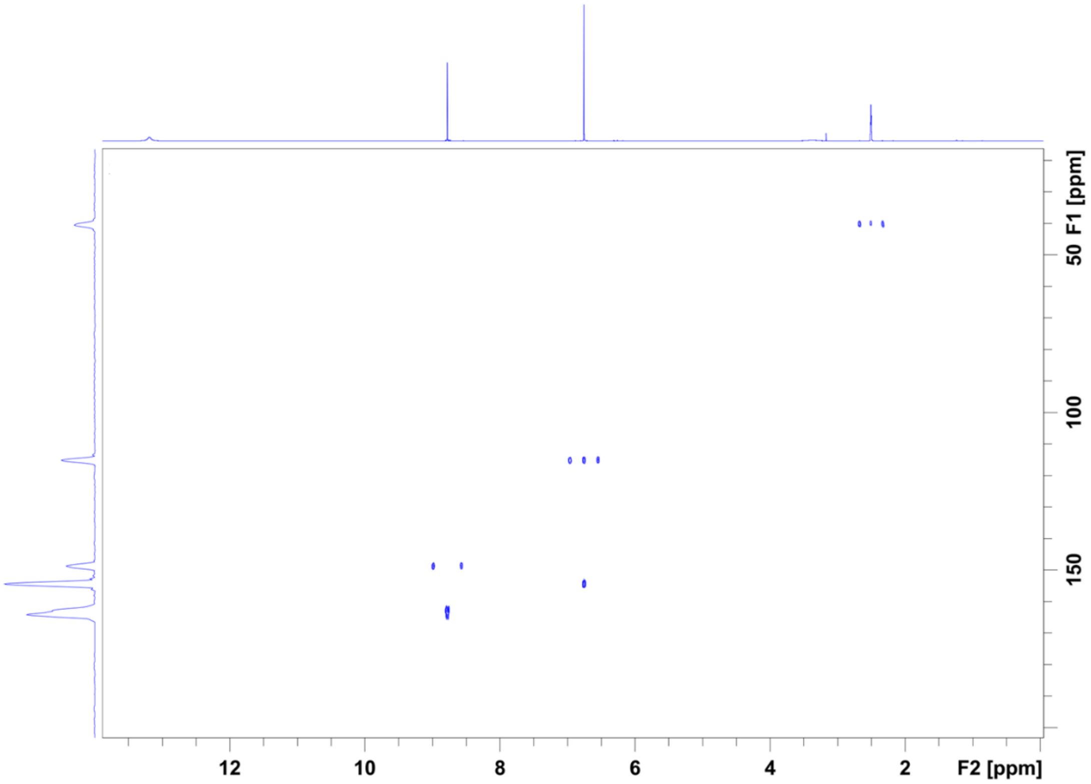

Figure S5 HMBC (DMSO-d_6) spectrum of **Cage-6-COOH**.

Section 4 Crystallization of Cage-6-COOH

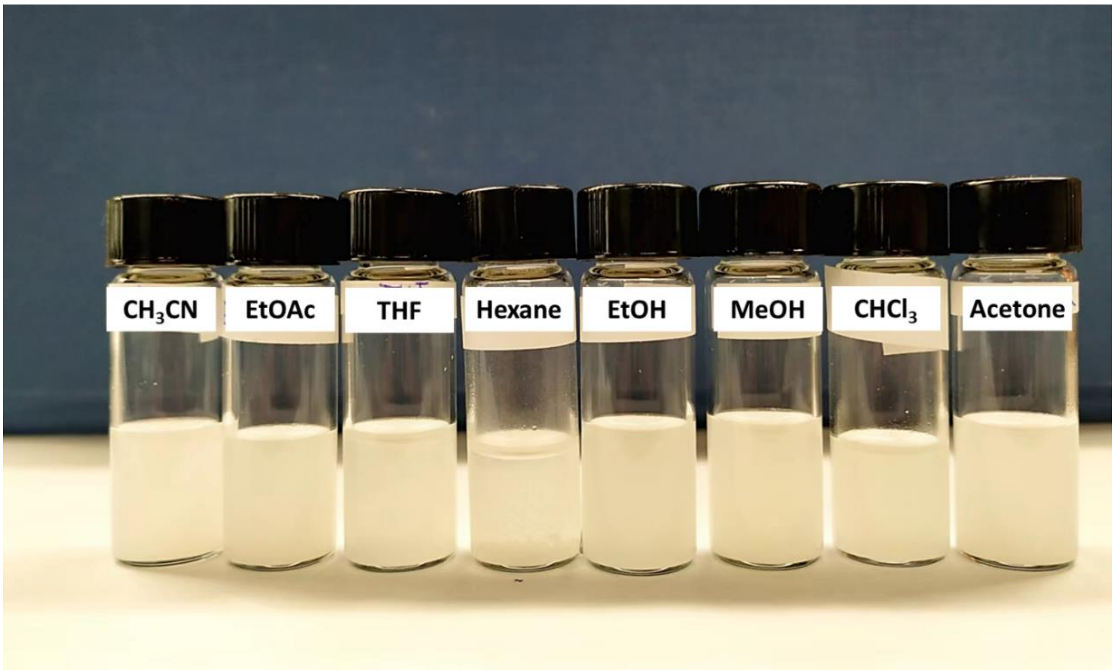

Figure S6 Solubility test of scCO_2-activated **CageHOF-2α** in various solvents, which was conducted by dissolving Cage-6-COOH (2 mg) in various solvents (2 mL) and sonicating for 10 min. The white suspension indicates Cage-6-COOH displays poor solubility in these solvents.

Crystallization procedures for CageHOF-2α

Cage-6-COOH (100 mg) was added to THF (150 mL) and sonicated for 10 mins to help dissolve the compound. The solution was filtered into a 500 mL round bottom bottle through a 0.45 um nanofilm and layered by 300 mL CH_3CN. The mixture was then sealed and left undisturbed for 5 days at room temperature, which afforded needle-like single crystals suitable for SCXRD analysis. Before gas sorption analysis, the crystallization solvents were exchanged with acetone (×10) and pentane (×10), and the crystals were then activated under dynamic vacuum conditions.

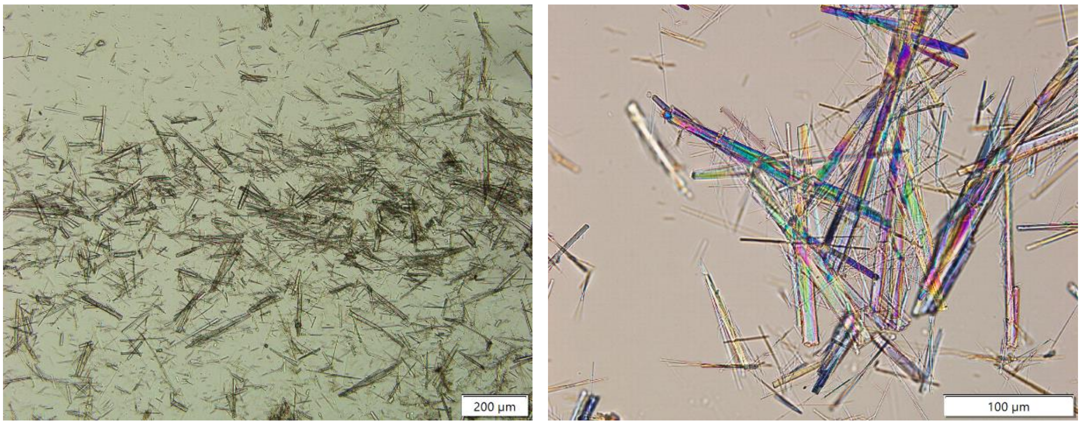

Figure S7 Optical images of **CageHOF-2α**.

Crystallization procedures for CageHOF-2β

Cage-6-COOH (10 mg) was dissolved in EtOH (20 mL) and sonicated for 5–10 min. The solution was filtered into 4 vials (15 mL) through a 0.45 μm syringe filter. The vials were then covered with a cap, which had a small hole on the top, and placed in an oven for 3 days at 70 °C, which afforded plate-like single crystals suitable for SCXRD analysis. The sample was activated after solvent exchanging the crystallization with acetone and pentane ten times within two days and used for further gas sorption analysis.

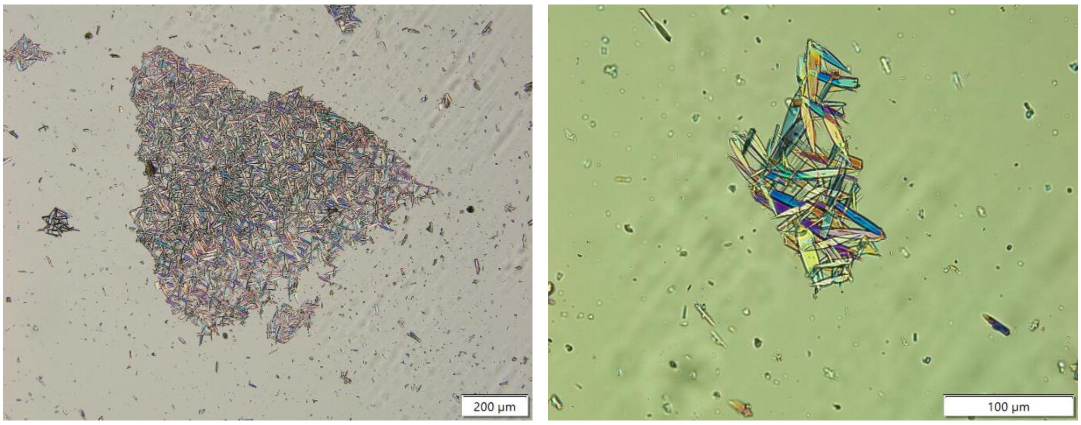

Figure S8 Optical images of **CageHOF-2β**.

Crystallization procedures for CageHOF-2·DMAc

Cage-6-COOH (10 mg) was dissolved in dimethylacetamide (DMAc, 4 mL) and sonicated for 5–10 min. The solution was filtered into two 4 mL glass vials through a 0.45 um PTFE syringe filter. The vials were then placed in a sealed chamber with acetone and left undisturbed, which afforded plate-like single crystals suitable for SCXRD analysis.

Crystallization of CageHOF-2·DESO and CageHOF-2·NMP via Encapsulated Nanodroplet Crystallization (ENaCt)

The crystallization of **CageHOF-2·DESO** and **CageHOF-2·NMP** were carried out using encapsulated nanodroplet crystallization (ENaCt) protocols.[26] Stock solutions were prepared by dispensing **Cage-6-COOH** into individual screw top vials (1 mg per vial) then different solvents (Table S1) were added in 12 µL portions to each vial until the sample was fully dissolved or until 192 µL of solvent was added. Where the samples were not fully soluble, the supernatant was taken forward to the ENaCt crystallization experiment.

Table S1 The type, volume and concentration of stock solutions used in the ENaCt experiments.

Solvent	Volume of solvent added to Cage-6-COOH / μL	Approximate concentration of Cage-6-COOH / mg mL^{-1}
Dimethyl sulfoxide (DMSO)	96	10.4
N,N-dimethylformamide (DMF)	96	10.4
Methanol (MeOH)	192[a]	5.2
1,1,1,3,3,3-hexafluoro-2-propanol (HFIP)	192[a]	5.2
Toluene	192[a]	5.2
1,2-dichloroethane (DCE)	192[a]	5.2
2-methyl tetrahydrofuran (2-MeTHF)	192[a]	5.2
tert-butyl methyl ether (MTBE)	192[a]	5.2
Ethyl acetate (EtOAc)	192[a]	5.2
Acetonitrile (MeCN)	192[a]	5.2
4-Methyl-2-pentanone (MIBK)	192[a]	5.2
Nitromethane (NM)	192[a]	5.2
Diethyl sulfoxide	48	20.8
Tetrahydrothiophene-1-oxide (THTP-1-oxide)	24	41.6
Glycol sulfite	192[a]	5.2
N-methyl imidazole (NMI)	72	13.9
Formamide	192[a]	5.2
Di-*iso*-propyl formamide	192[a]	5.2
N-methyl-2-pyrrolidone (NMP)	192	5.2

[a]Supernatant was taken forward for ENaCt experiments.

The stock solutions of **Cage-6-COOH** (50 nL) were dispensed via an SPT Labtech Mosquito liquid handling robot into 96-well glass plates (SWISSCI LCP Modular, 100 mm spacer) containing either an appropriate crystallization oil (200 nL) or no oil (Table S2). For wells containing oil, the oil (PDMSO, FC-40, Fomblin-Y or mineral oil) was dispensed prior to injection of the stock solution into the oil droplet. Plates were sealed with a glass cover slip and allowed to stand undisturbed at room

temperature in the dark. After 14 days, the crystallization wells were assessed visually and by cross-polarized light microscopy for crystal growth. From 480 individual ENaCt experiments across five plates, 19 wells (4.0 %) contained large single crystals (class 4; Table S3) suitable for SCXRD analysis. A single crystal of **CageHOF-2·DESO** was grown from diethyl sulfoxide (50 nL, 20.8 mg mL^{-1}) encapsulated in a 200 nL droplet of PDMSO (Plate 4, well C4) and a single crystal of **CageHOF-2·NMP** was grown from *N*-methyl pyrrolidinone (50 nL, 5.2 mg mL^{-1}) encapsulated in a 200 nL droplet of FC-40 (Plate 5, well H4) which were subsequently analysed by SCXRD.

Wells were opened with use of a tungsten carbide scriber to remove a small portion of the glass cover slide, and the crystal manipulated using MiTeGen Kapton microtools. Crystals were transferred to a glass slide and extracted under oil (Fomblin YR-1800) and mounted onto a 35 µm MiTeGen Kapton loop before being flash-cooled to 150 K under N_2 using an Oxford Cryosystems cryostream before the collection.

Table S2 The standard plate layout used for the ENaCt experiments.

Plate number														
			Volume of Oil (200 nL)											
	Volume of Solvent (50 nL)		1	2	3	4	5	6	7	8	9	10	11	12
1	Solvent A	A	No oil	PDMSO					No oil	Fomblin-Y				
		B	No oil	FC-40					No oil	Mineral oil				
2	Solvent B	C	No oil	PDMSO					No oil	Fomblin-Y				
		D	No oil	FC-40					No oil	Mineral oil				
3	Solvent C	E	No oil	PDMSO					No oil	Fomblin-Y				
		F	No oil	FC-40					No oil	Mineral oil				
4	Solvent D	G	No oil	PDMSO					No oil	Fomblin-Y				
		H	No oil	FC-40					No oil	Mineral oil				

Table S3 Crystallization results for ENaCt plates 1–5 after two weeks. Key: F = failed to dispense correctly due to liquid handling robot failure; 1 = still solvated; 2 = non-crystalline or amorphous material; 3 = microcrystalline material; 4 = single crystals suitable for SCXRD studies.

Plate 1

	Solvent		1	2	3	4	5	6	7	8	9	10	11	12
1	dimethyl sulfoxide	A	2	2	2	2	2	2	2	2	2	3	3	2
	dimethyl sulfoxide	B	3	2	2	3	2	2	2	3	2	2	2	2
2	N,N-dimethylformamide	C	2	2	2	2	2	2	2	2	2	2	2	2
	N,N-dimethylformamide	D	2	2	2	2	2	2	2	2	2	2	2	2
3	Methanol	E	2	2	2	2	2	2	2	1	1	1	1	1
	Methanol	F	2	F	2	2	2	2	2	2	1	1	1	1
4	1,1,1,3,3,3-hexafluoro-2-propanol	G	2	2	2	2	2	2	2	2	2	2	2	2
	1,1,1,3,3,3-hexafluoro-2-propanol	H	2	2	2	2	2	2	2	2	2	2	2	2

Plate 2

	Solvent		1	2	3	4	5	6	7	8	9	10	11	12
1	toluene	A	1	1	2	1	2	2	1	1	1	1	1	1
	toluene	B	1	1	1	1	1	1	1	1	1	1	1	1
2	1,2-dichloroethane	C	1	1	1	1	1	1	1	1	1	1	1	1
	1,2-dichloroethane	D	1	1	1	1	1	1	1	1	1	1	1	1
3	2-methyl tetrahydrofuran	E	1	1	1	1	1	1	1	1	1	1	1	1
	2-methyl tetrahydrofuran	F	1	1	1	1	1	1	1	1	1	1	1	1
4	*tert*-butyl methyl ether	G	1	1	1	1	1	1	1	1	1	1	1	1
	tert-butyl methyl ether	H	1	1	1	1	1	1	1	1	1	1	1	2

Plate 3

	Solvent		1	2	3	4	5	6	7	8	9	10	11	12
1	ethyl acetate	A	1	1	1	1	1	1	1	1	1	1	1	1
	ethyl acetate	B	1	1	1	1	1	1	1	1	1	1	1	1
2	acetonitrile	C	1	1	1	1	1	1	1	1	1	2	1	1
	acetonitrile	D	1	1	1	1	1	1	1	1	1	1	1	1
3	4-Methyl-2-pentanone	E	1	1	1	1	1	1	1	1	1	1	1	1
	4-Methyl-2-pentanone	F	1	1	1	1	1	1	1	1	1	1	1	1
4	nitromethane	G	1	1	1	1	1	1	1	1	1	1	1	1
	nitromethane	H	1	1	1	1	1	1	1	1	1	1	1	1

Plate 4

	Solvent		1	2	3	4	5	6	7	8	9	10	11	12
1	dimethyl sulfoxide	A	2	2	2	2	2	2	2	1	1	1	2	2
	dimethyl sulfoxide	B	2	2	2	2	2	2	2	1	1	1	1	1
2	diethyl sulfoxide	C	4	3	4	4	4	4	3	1	1	2	1	2
	diethyl sulfoxide	D	4	2	2	2	2	4	3	3	3	4	4	4
3	tetrahydrothiophene-1-oxide	E	1	1	1	1	1	1	1	1	1	1	1	1
	tetrahydrothiophene-1-oxide	F	1	1	1	1	1	1	1	1	1	1	1	1
4	glycol sulfite	G	1	1	1	2	2	2	2	1	2	1	1	1
	glycol sulfite	H	2	2	1	2	1	2	2	1	1	1	1	1

Plate 5

	Solvent		1	2	3	4	5	6	7	8	9	10	11	12
1	N-methyl imidazole	A	1	1	1	1	1	1	1	1	1	1	1	1
	N-methyl imidazole	B	1	1	1	1	1	1	1	1	1	1	1	1
2	formamide	C	2	2	2	2	2	2	2	2	2	2	2	2
	formamide	D	2	2	2	2	2	2	2	2	2	2	2	2
3	di-*iso*-propyl formamide	E	2	1	1	2	2	2	2	1	2	2	1	1
	di-*iso*-propyl formamide	F	2	1	1	2	2	2	1	1	1	2	1	1
4	N-methyl-2-pyrrolidone	G	3	4	4	3	4	3	3	1	1	3	1	4
	N-methyl-2-pyrrolidone	H	3	4	3	4	3	4	3	3	3	4	3	4

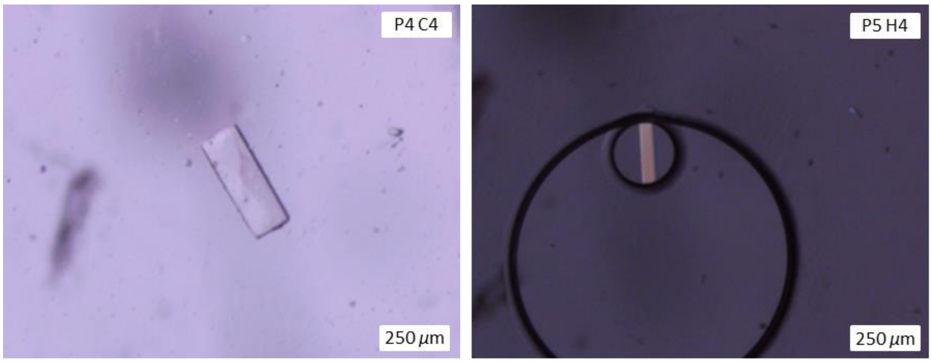

Figure S9 Optical images of **CageHOF-2·DESO** (left) and **CageHOF-2·NMP** (right).

Section 5 Characterization of CageHOF-2α

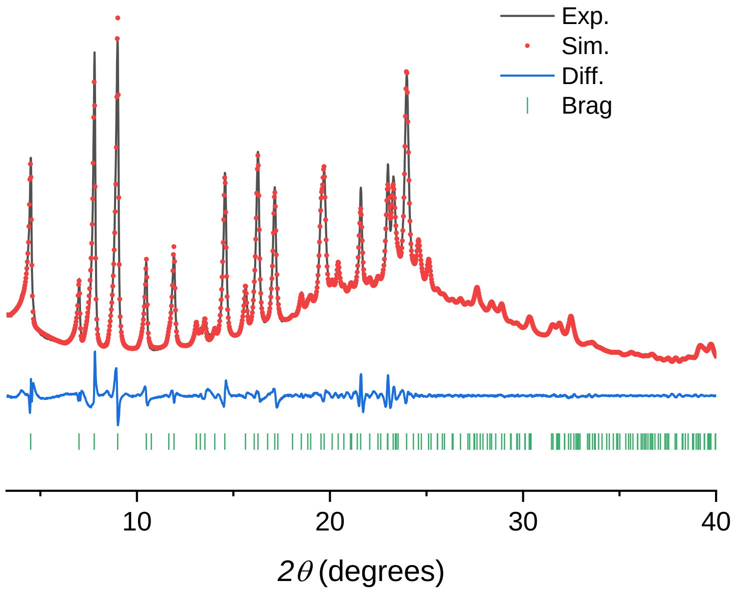

Figure S10 Pawley fitting of **CageHOF-2α** using structural parameters from SCXRD data. Black line: solvated experimental PXRD pattern, red points: fitting pattern, blue curve: the difference between experimental and refinement, green bars: reflection positions. A low value of R_{wp} = 3.55% and R_p = 2.29% confirms the high phase purity of **CageHOF-2α** powder, yielding a cell parameter of $P\bar{6}2c$, a = b =22.67 Å, c = 16.45 Å, V = 7320 Å^3. The CH_3CN-solvated sample was loaded in borosilicate glass capillaries and ran at room temperature.

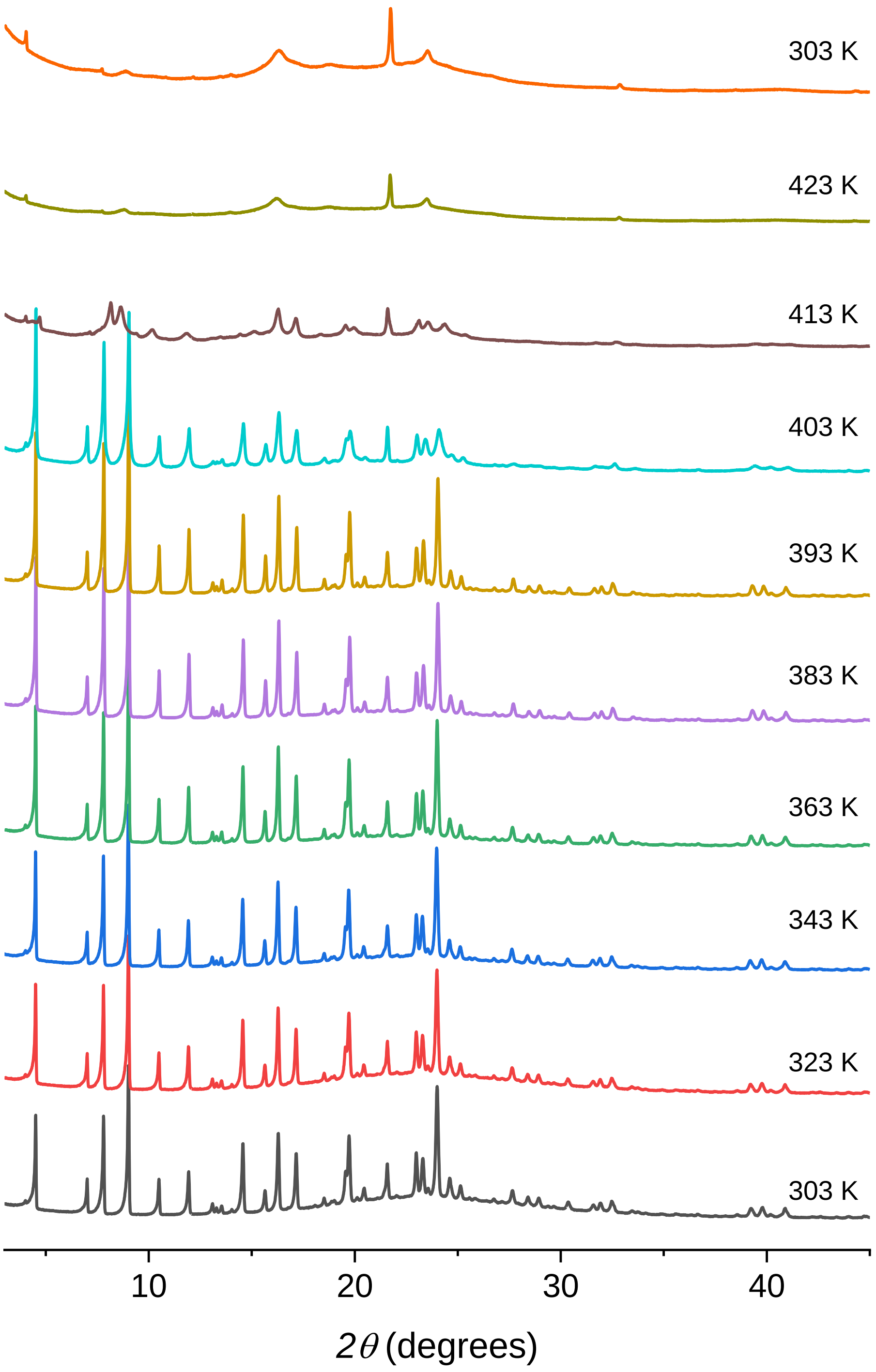

Figure S11 *In situ* VT-PXRD patterns recorded while heating a CH_3CN-solvated sample of **CageHOF-2α**. PXRD patterns were recorded every 10 or 20 K after equilibrating the sample temperature for 10 minutes.

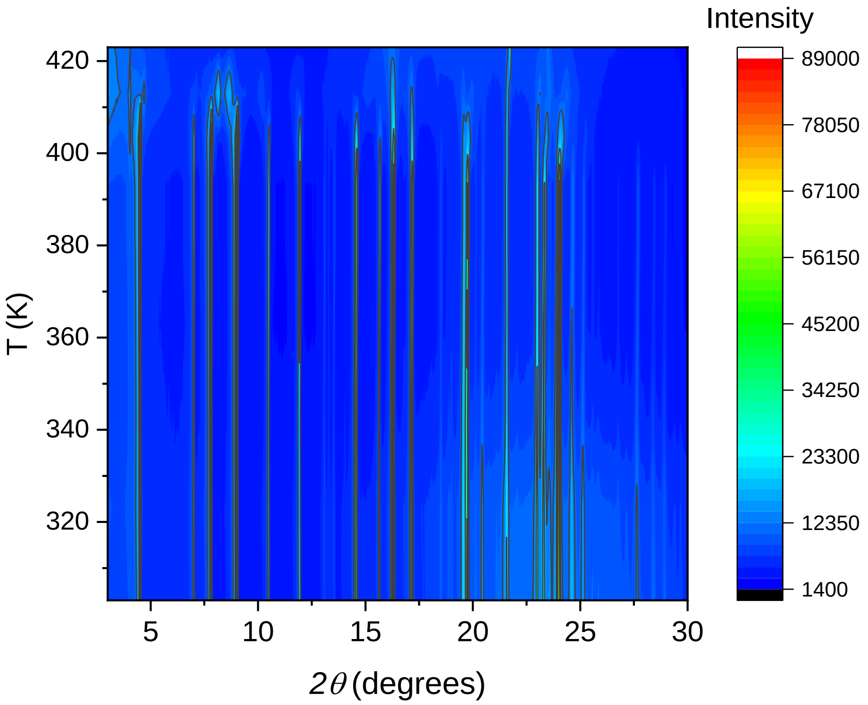

Figure S12 Surface plot of VT-PXRD patterns of CH_3CN-solvated **CageHOF-2α** recorded over the temperature range 303–423 K. PXRD patterns were recorded every 10 or 20 K, after equilibrating the sample temperature for 10 minutes.

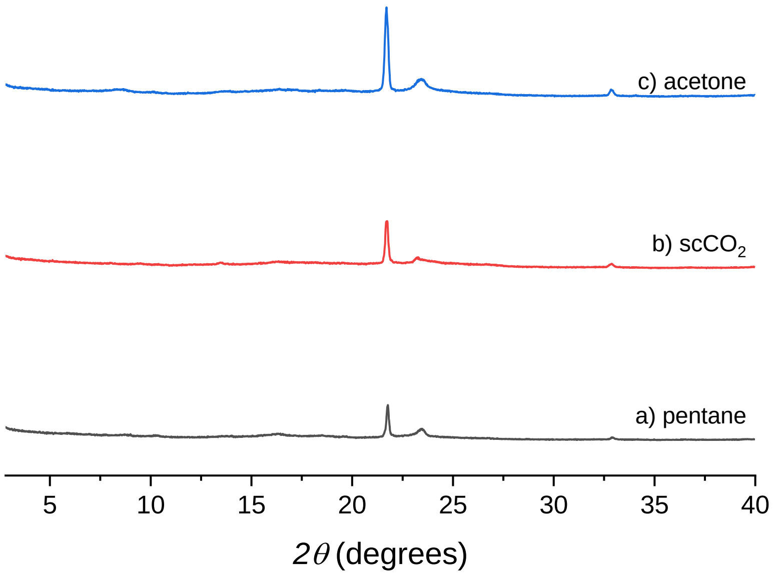

Figure S13 PXRD patterns of **CageHOF-2α** recorded after various activation treatments. We used anhydrous and dry conditions during all the activation processes to avoid water affecting the stability of the hydrogen-bonded structure. We attribute the diffraction peak at 2θ (Cu-Kα) = 21.7 ° to the (004) reflection, which aligns with the packing of **Cage-6-COOH** layers in the **CageHOF-2α** structure.

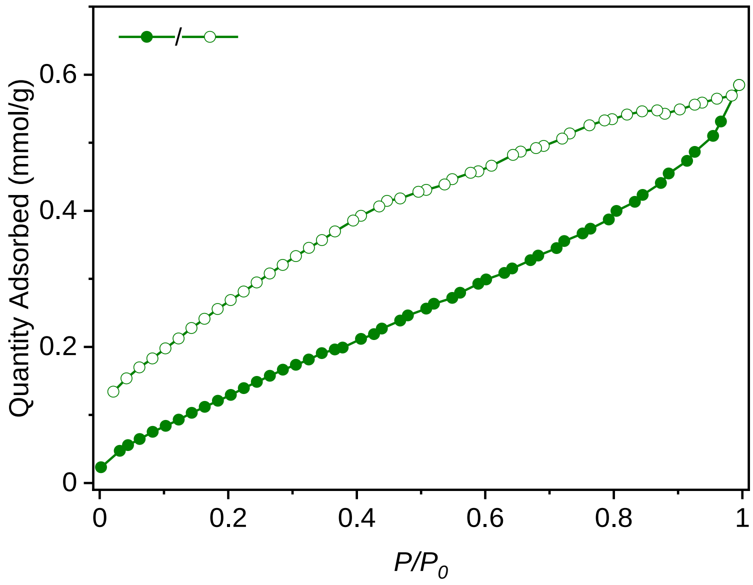

Figure S14 N_2 sorption isotherms of scCO_2 activated **CageHOF-2α** powder at 77 K.

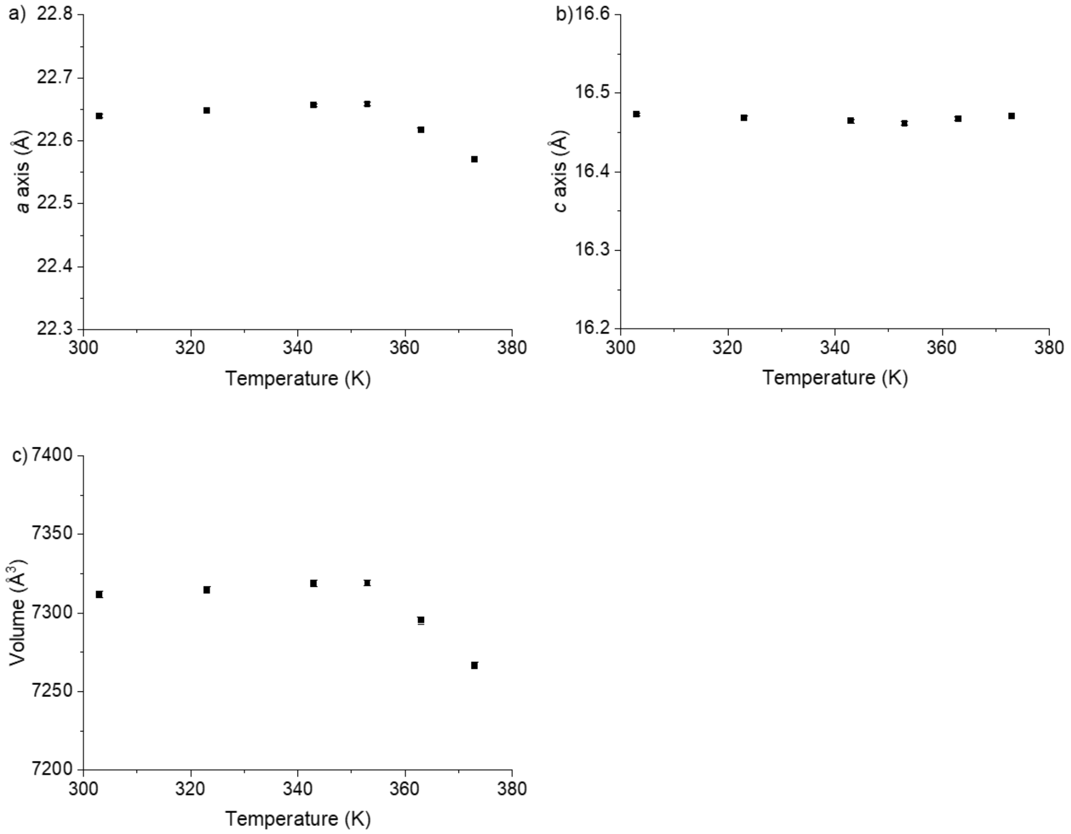

Figure S15 Unit cell information from the *in situ* VT-PXRD patterns that were recorded while heating a CH_3CN solvated **CageHOF-2α** sample loaded in a glass capillary. The sample temperature was equilibrated at each value for 10 mins before each 30 mins PXRD pattern was recorded.

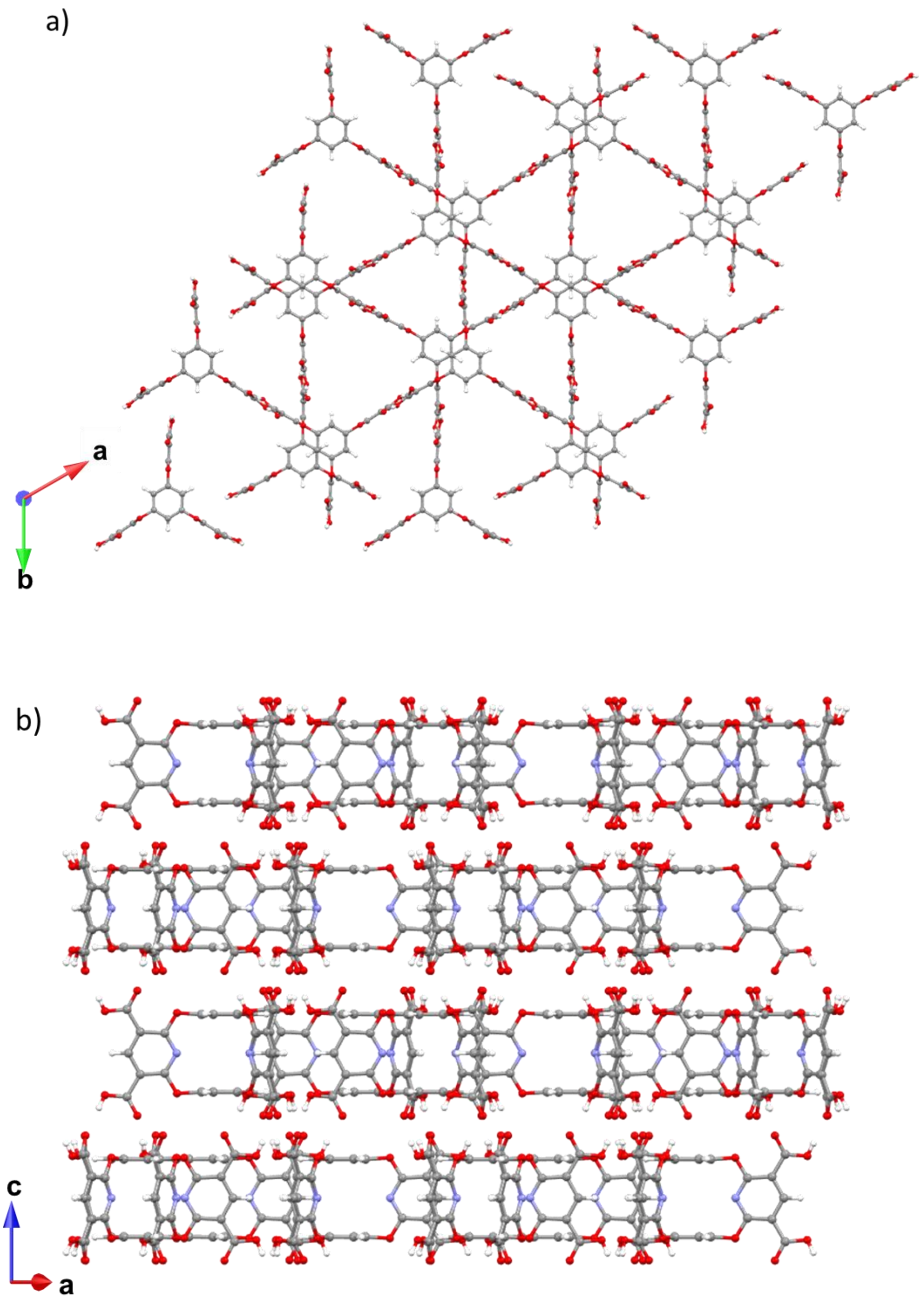

Figure S16 Crystal packing in the SCXRD structure of **CageHOF-2α** shown (a) along the crystallographic *c*-axis, and (b) along the crystallographic *a*-axis. In the crystal structure, the **Cage-6-COOH** molecule are less densely packed along the *ab* directions, generating large 1-D pores. By contrast, layers of **Cage-6-COOH** molecules are more densely packed along *c*.

Section 6 Characterization of CageHOF-2β

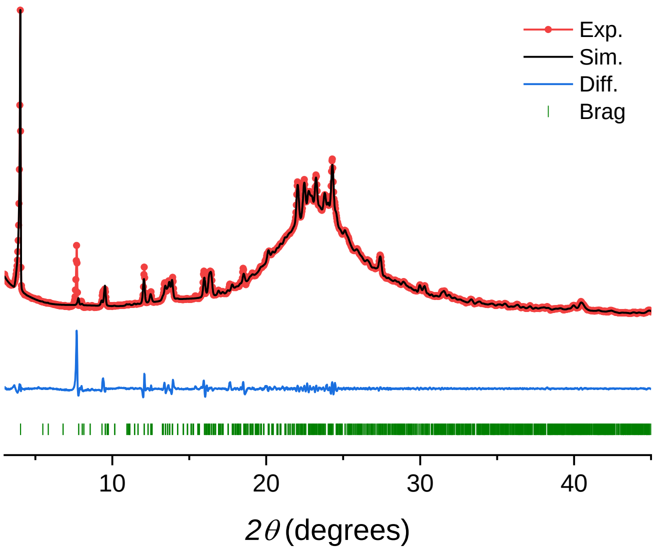

Figure S17 Rietveld refinement of **CageHOF-2β** (R_{wp} = 4.26% and R_p = 1.62%, $P2_12_12$, a = 44.06 Å, b =11.74 Å, c = 16.19 Å, $\alpha = \beta = \gamma$ = 90°, V = 8374.5 Å^3). Black line: solvated simulated PXRD pattern, red points: experimental pattern, blue curve: the difference between experimental and refinement, green bars: reflection positions. For the measurement, we loaded the EtOH-solvated **CageHOF-2β** material into a borosilicate glass capillary and recorded the PXRD patterns at room temperature. Due to the large size of the asymmetric unit (108 non-H atoms, excluding solvent) and the flexibility of the cage and crystal structure, we performed the Rietveld refinement using a rigid body restraint where all the atom positions were constrained.

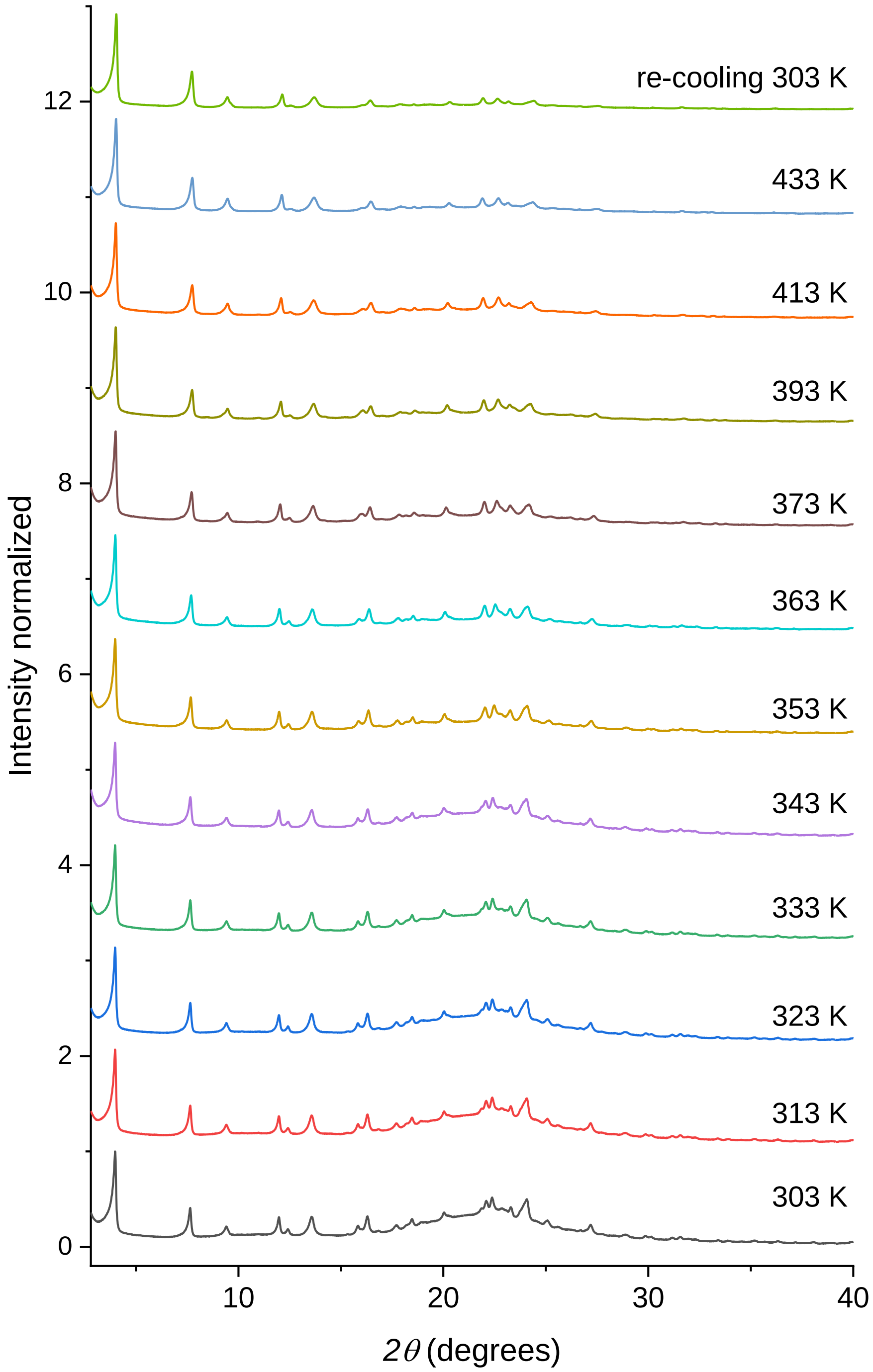

Figure S18 *In situ* VT-PXRD patterns that were recorded while heating an EtOH-solvated sample of **CageHOF-2β**. We recorded the PXRD patterns every 10 or 20 K after equilibrating the sample temperature at each value for 10 minutes.

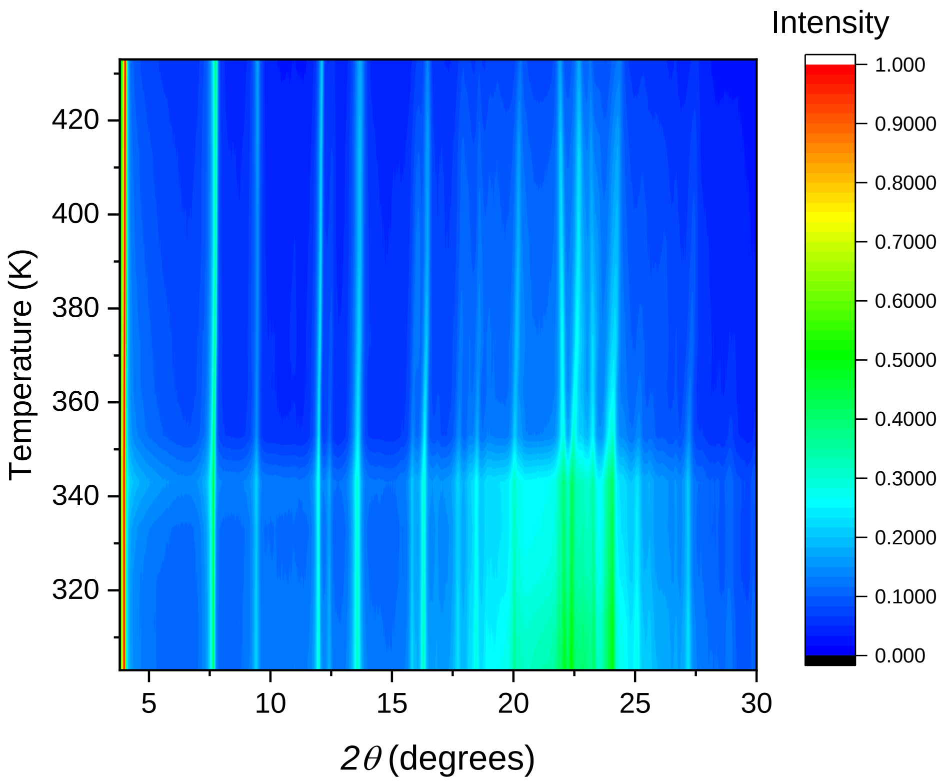

Figure S19 Surface plot of VT-PXRD patterns of EtOH-solvated **CageHOF-2β** recorded while heating a sample loaded in a glass capillary over the temperature range 303–433 K. PXRD patterns were recorded every 10 or 20 K after equilibrating the sample temperature for 10 minutes.

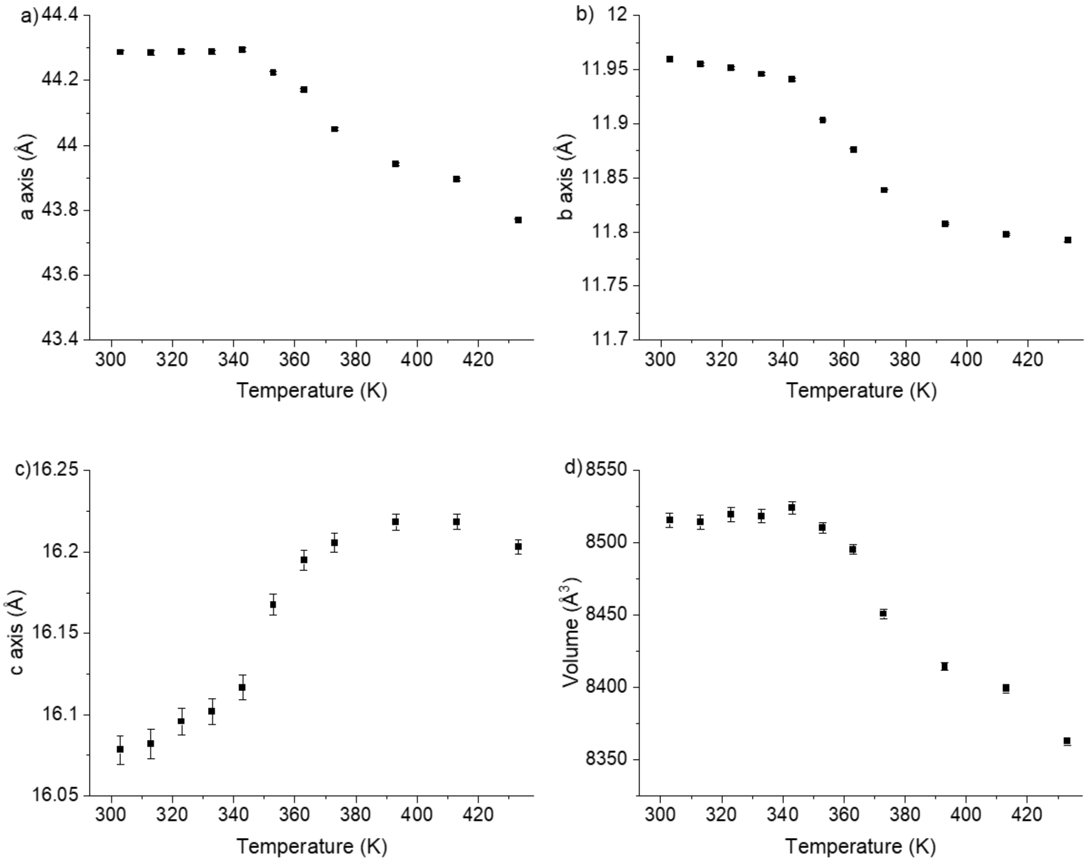

Figure S20 Changes in unit cell parameter for EtOH-solvated **CageHOF-2β** recorded while heating a sample loaded in a glass capillary over the temperature range 303–433 K; (a) *a*-axis, (b) *b*-axis, (c) *c*-axis, and (d) unit cell volume.

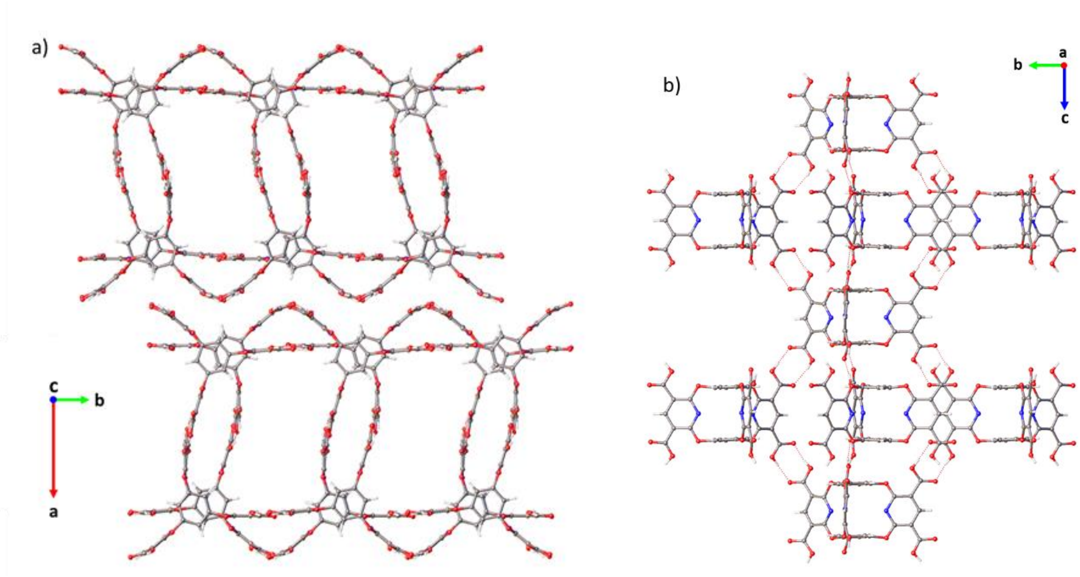

Figure S21 Crystal packing in the SCXRD structure of **CageHOF-2β** shown along the (a) *c*-unit cell axis and (b) *a*-unit cell axis. The VT-PXRD data indicates that the crystal structure expands along the c-axis slightly during the thermal removal of solvent from the crystal pores.

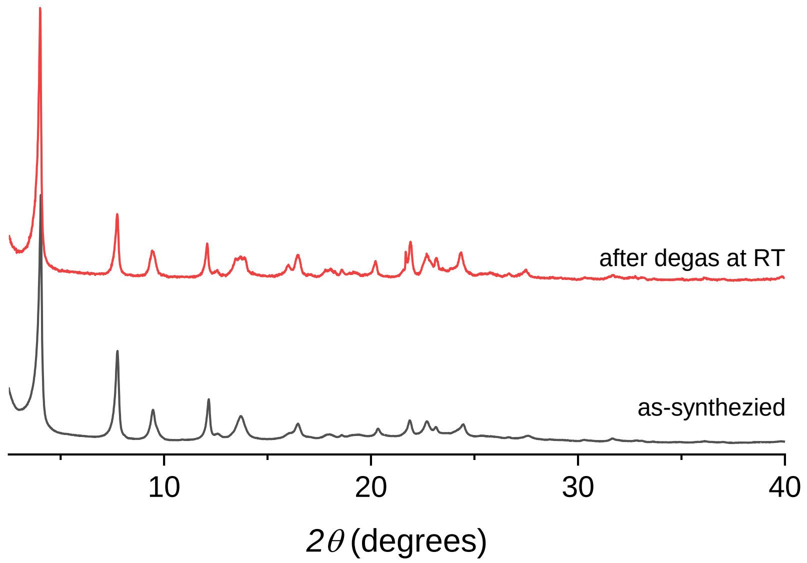

Figure S22 PXRD patterns comparison of **CageHOF-2β** between the solvate sample and the powder after the treatment with acetone, pentane and drying under vacuum.

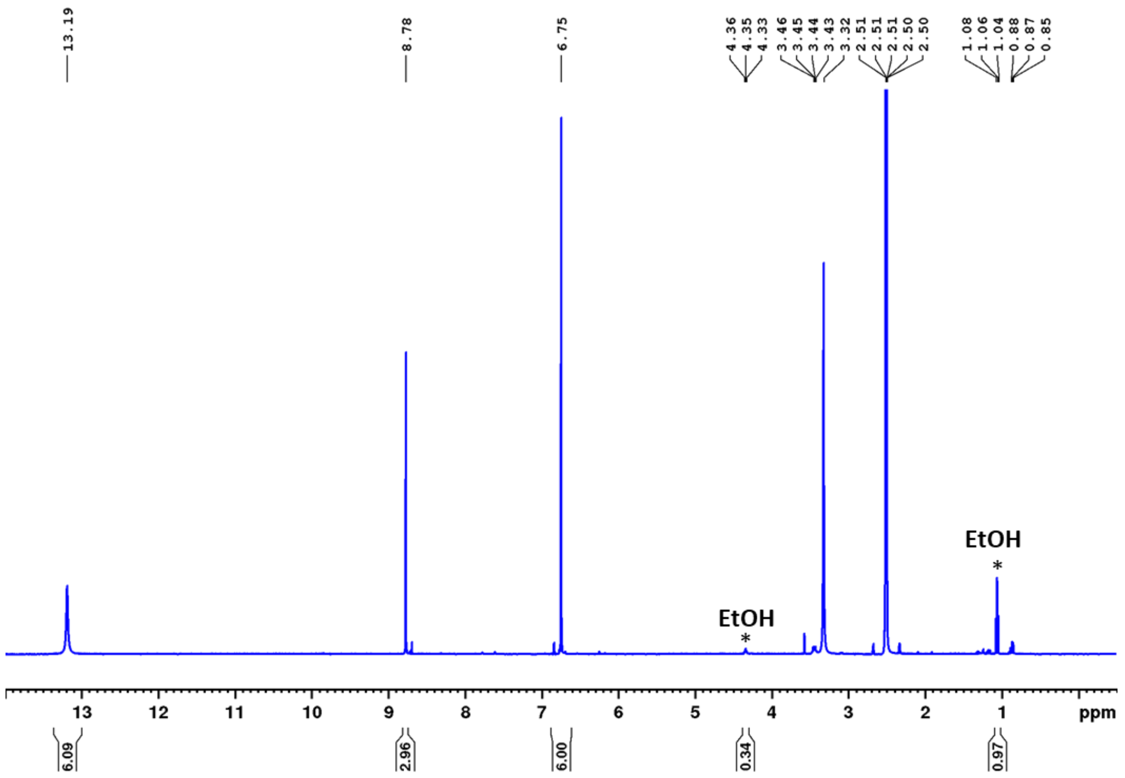

Figure S23 NMR spectrum of **CageHOF-2β** measured after N_2 sorption. The NMR spectrum indicates that the activated **CageHOF-2β** still comprises a trace amount of EtOH molecule (3.5 wt.%).

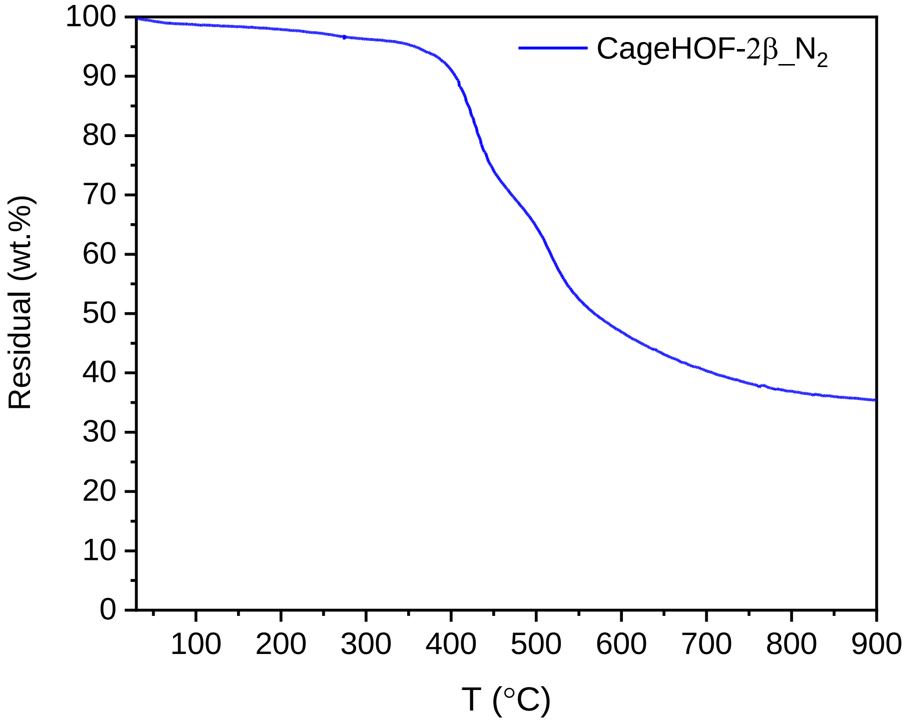

Figure S24 TGA plot of **CageHOF-2β** measured after N_2 sorption; a 5% weight loss was observed before 450 °C.

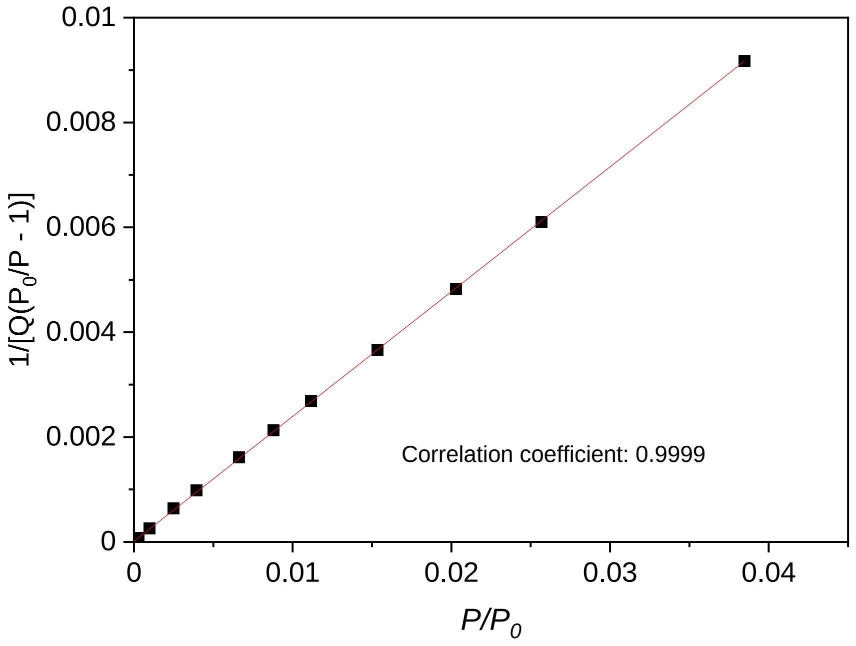

Figure S25 t-plot for the BET surface areas calculation of **CageHOF-2β**.

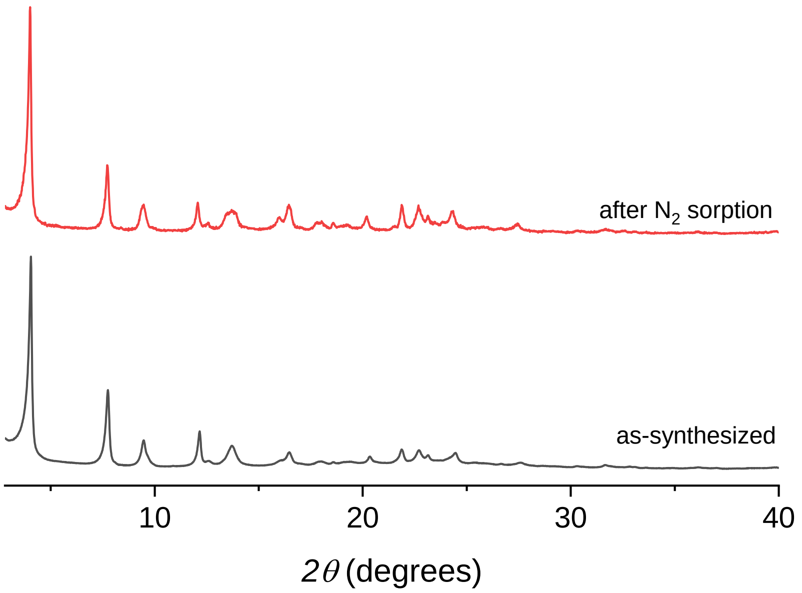

Figure S26 PXRD pattern of **CageHOF-2β** recorded after N_2 sorption analysis.

Section 7 Structural information

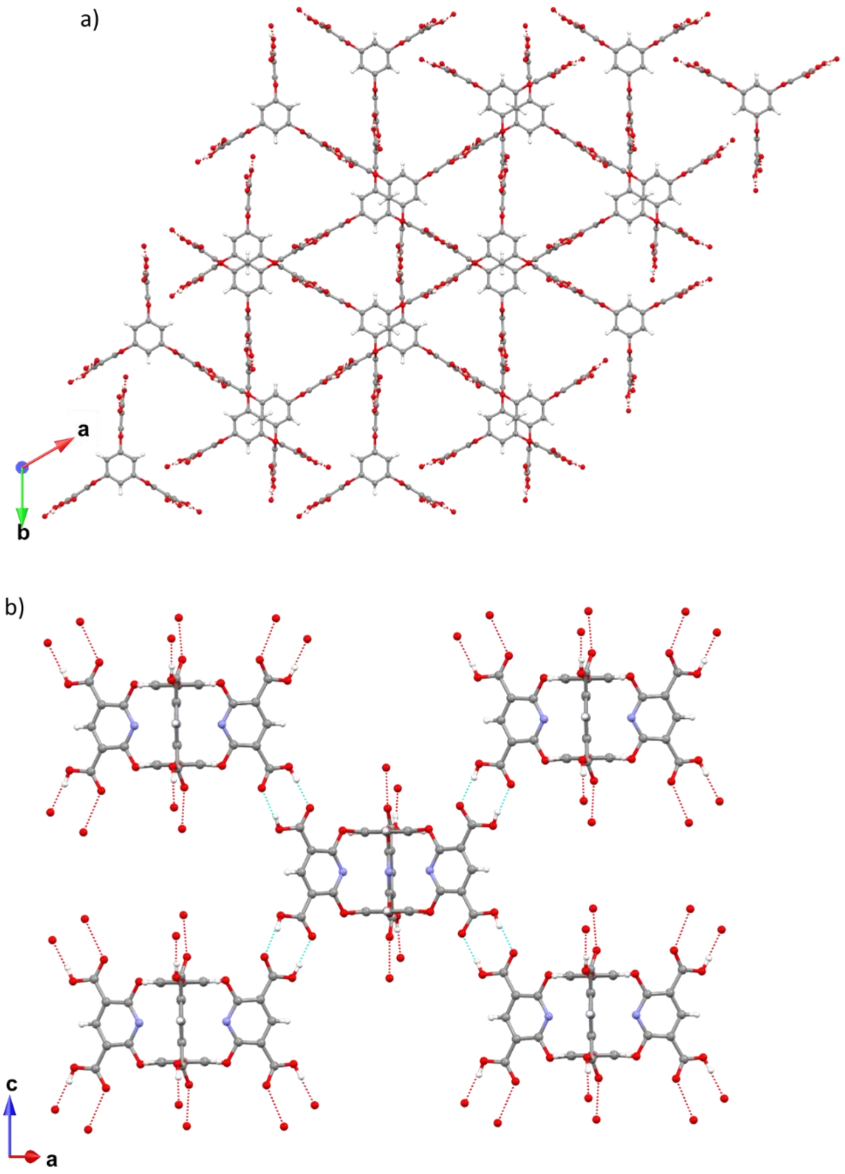

Figure S27 Illustration for the single crystal structure of **CageHOF-2α**. Two views are shown, top view (a), side view (b). Atoms, H = white; C = grey; N = blue; O = red.

Table S4. Single crystal refinement details for **CageHOF-2α.**

**Name**	**CageHOF-2α**
**Crystallization solvent**	THF-CH_3CN
**Wavelength/ Å**	Mo-Kα, 0.71073
**Formula**	C_{33} H_{15} N_3 O_{18}
**Weight**	741.48
**Crystal system**	hexagonal
**Space group**	$P\bar{6}2c$
$\boldsymbol{a = b}$ **(Å)**	22.6444(8)
$\boldsymbol{c}$ **(Å)**	16.4319(5)
$\boldsymbol{\alpha = \beta}$ **(°)**	90
$\boldsymbol{\gamma}$ **(°)**	120
$\boldsymbol{V}$ **(Å^3)**	7296.9(6)
$\boldsymbol{\rho}$ **calcd (g cm^{-1})**	1.012
$\boldsymbol{Z}$	6
$\boldsymbol{T}$ **(K)**	300
$\boldsymbol{\mu}$ **(mm^{-1})**	0.085
$\boldsymbol{F}$**(000)**	2268
$\boldsymbol{\theta}$ **range (°)**	2.688–27.534
**Reflections collected**	59047
**Independent reflections**	5733
**Obs. Data [$I > 2\sigma$]**	3550
**Data / restraints / parameters**	5733 / 0 / 253
$\boldsymbol{R_{int}}$	0.0513
**Final R_1 values ($I > 2\sigma(I)$)**	0.0672
**Final R_1 values (all data)**	0.1034
**wR_2 (all data)**	0.2084
**Goodness-of-fit on $\lvert F\rvert^2$**	1.043
**CCDC**	2253692

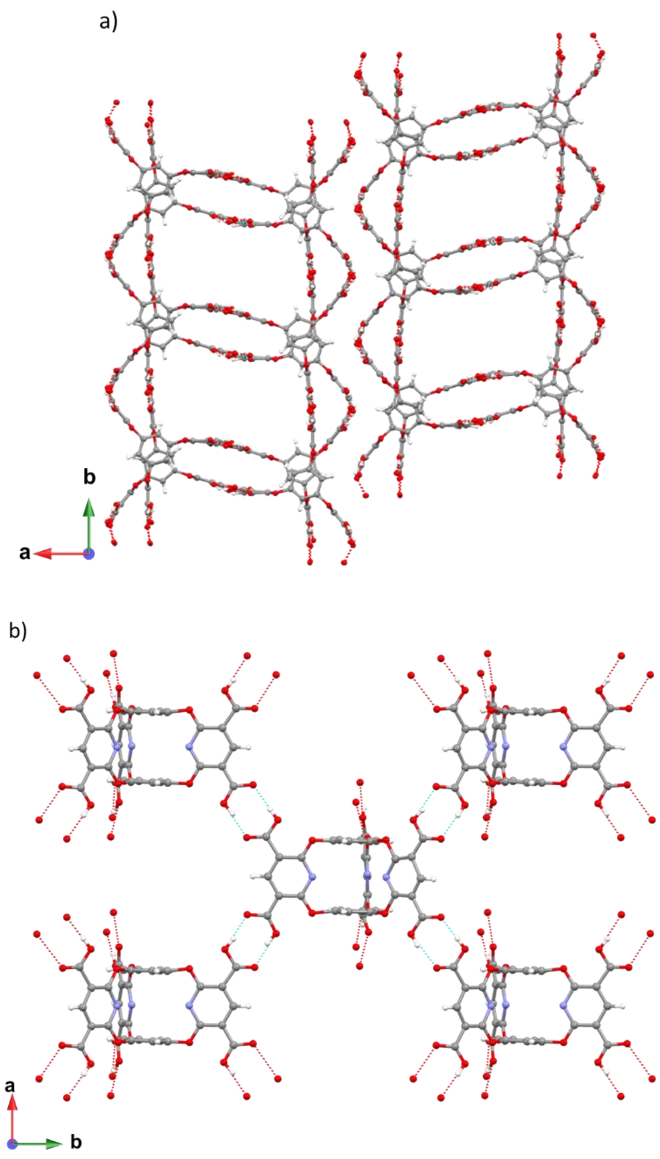

Figure S28 Crystal packing in the SCXRD structure of **CageHOF-2β** shown along the (a) *c*-unit cell axis, and b) *a*-unit cell axis. VT-PXRD data indicates that the crystal structure expands along the *c*-axis slightly during thermal removal of solvent from the crystal pores. Atoms, H = white; C = grey; N = blue; O = red.

Table S5. Single crystal refinement details for **CageHOF-2β**.

**Name**	**CageHOF-2β**
**Crystallization solvent**	EtOH
**Wavelength/ Å**	Mo-Kα, 0.71073
**Formula**	$C_{33} H_{15} N_3 O_{18}$
**Weight**	741.48
**Crystal system**	orthorhombic
**Space group**	$P2_12_12$
a **(Å)**	43.576(4)
b **(Å)**	11.7207(9)
c **(Å)**	16.2030(8)
$\alpha = \beta = \gamma$ **(°)**	90
V **(Å^3)**	8275.5(11)
ρ_{calcd} **(g cm^{-1})**	1.190
Z	8
T **(K)**	300
μ **(mm^{-1})**	0.1
F**(000)**	3024
θ **range (°)**	1.869–23.303
**Reflections collected**	49644
**Independent reflections**	11849
**Obs. Data [$I > 2\sigma$]**	4829
**Data / restraints / parameters**	11849 / 1259 / 902
**Final R_1 values ($I > 2\sigma(I)$)**	0.1039
**Final R_1 values (all data)**	0.1741
**wR_2 (all data)**	0.2675
**Goodness-of-fit on $\|F\|^2$**	1.063
**CCDC**	2253694

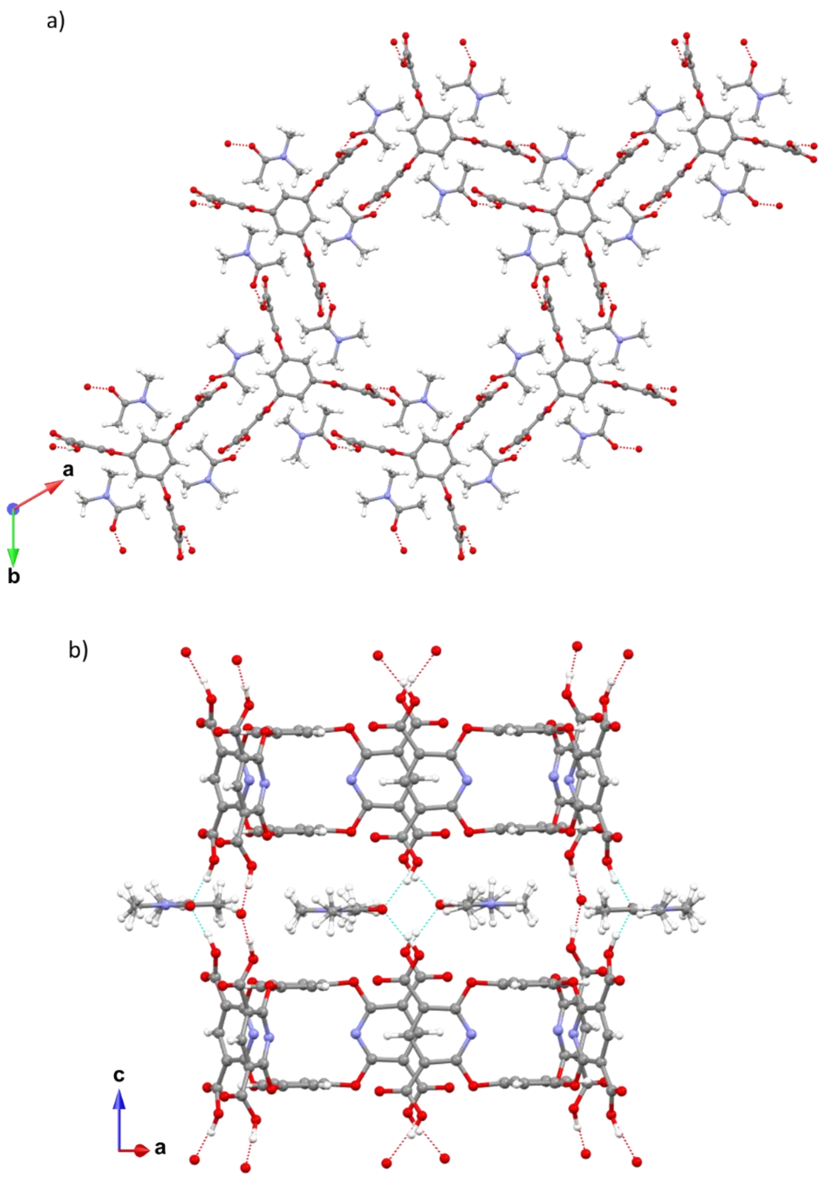

Figure S29 Illustration for the single crystal structure of **CageHOF-2·DMAc**. Two views are shown, top view (a), side view (b). Atoms, H = white; C = grey; N = blue; O = red.

Table S6. Single crystal refinement details for **CageHOF-2·DMAc**.

**Name**	**CageHOF-2·DMAc**
**Crystallization solvent**	DMAc, acetone
**Wavelength/ Å**	Mo-Kα, 0.71073
**Formula**	$C_{33}H_{15}N_3O_{18}$, 1.5($C_8H_{18}N_2O_2$)
**Weight**	1002.84
**Crystal system**	hexagonal
**Space group**	$P6/m$
$a = b$ **(Å)**	18.8265(18)
c **(Å)**	11.1935(8)
$\alpha = \beta$ **(°)**	90
γ **(°)**	120
V **(Å^3)**	3435.9(7)
ρ_{calcd} **(g cm^{-1})**	0.969
Z	2
T **(K)**	200
μ **(mm^{-1})**	0.078
F**(000)**	1044.0
θ **range (°)**	1.819–21.950
**Reflections collected**	12711
**Independent reflections**	1491
**Obs. Data [$I > 2\sigma$]**	1112
**Data / restraints / parameters**	1491 / 33 /128
**Final R_1 values ($I > 2\sigma(I)$)**	0.0732
**Final R_1 values (all data)**	0.0950
wR_2 **(all data)**	0.1989
**Goodness-of-fit on $\|F\|^2$**	1.060
**CCDC**	2253693

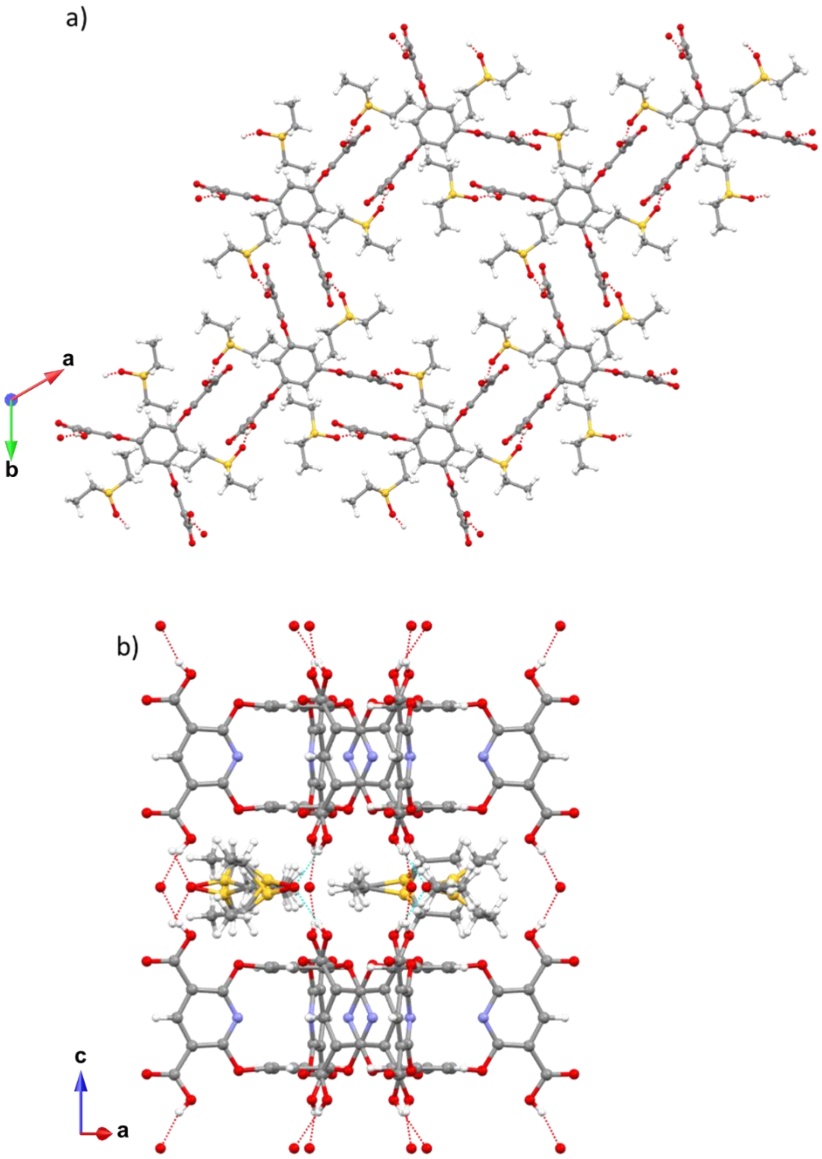

Figure S30 Illustration for the single crystal structure of **CageHOF-2·DESO**. Two views are shown, top view (a), side view (b). Atoms, H = white; C = grey; N = blue; O = red; S = yellow.

Table S7. Single crystal refinement details for **CageHOF-2·DESO**.

**Name**	**CageHOF-2·DESO**
**Crystallization solvent**	DESO
**Wavelength/ Å**	Cu-Kα, 1.54184
**Formula**	$C_{33} H_{15} N_3 O_{18}$, 1.5($C_8 H_{20} O_2 S_2$)
**Weight**	1060.02
**Crystal system**	hexagonal
**Space group**	*P*6/m
***a* = *b* (Å)**	18.3738(2)
***c* (Å)**	11.2811(2)
***α* = *β* (°)**	90
***γ* (°)**	120
***V* (Å^3)**	3298.22(9)
**ρ_{calcd} (g cm^{-1})**	1.067
Z	2
***T* (K)**	150
**μ (mm^{-1})**	1.570
***F*(000)**	1104.0
**θ range (°)**	4.805–77.15
**Reflections collected**	35018
**Independent reflections**	2392
**Obs. Data [$I > 2\sigma$]**	2148
**Data / restraints / parameters**	2392/ 115/ 135
**Final R_1 values ($I > 2\sigma(I)$)**	0.0610
**Final R_1 values (all data)**	0.0646
**wR_2 (all data)**	0.1989
**Goodness-of-fit on $\|F\|^2$**	1.083
**CCDC**	2290411

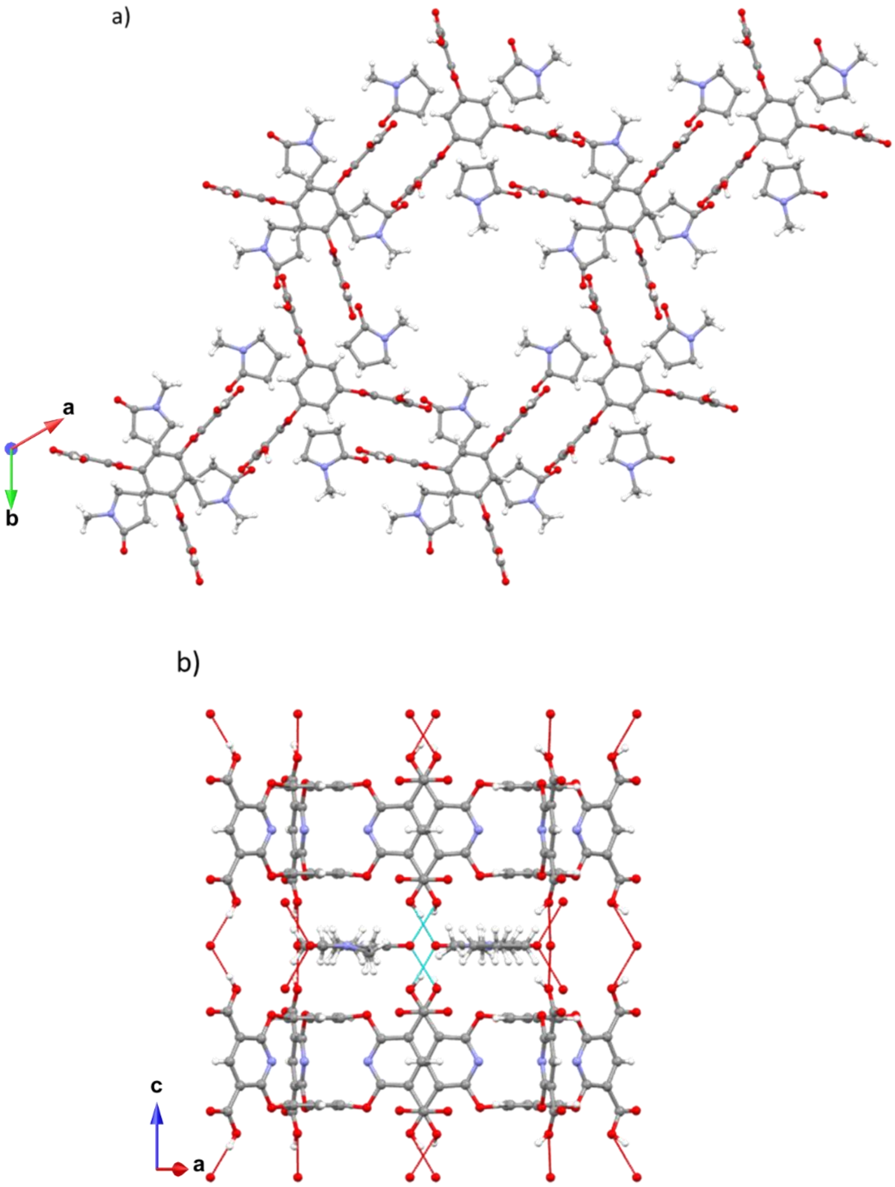

Figure S31 Illustration for the single crystal structure of **CageHOF-2·NMP**. Two views are shown, top view (a), side view (b). Atoms, H = white; C = grey; N = blue; O = red.

Table S8. Single crystal refinement details for **CageHOF-2·NMP**.

**Name**	**CageHOF-2·NMP**
**Crystallization solvent**	NMP
**Wavelength/ Å**	CuKα, 1.54184
**Formula**	$C_{33} H_{15} N_3 O_{18}$, 3($C_5 H_9$ N O)
**Weight**	1038.87
**Crystal system**	hexagonal
**Space group**	*P*6/*m*
***a* = *b* (Å)**	18.5271(3)
***c* (Å)**	11.3256(2)
***α* = *β* (°)**	90
***γ* (°)**	120
***V* (Å^3)**	3366.72(12)
**ρ_{calcd} (g cm^{-1})**	1.025
Z	2
***T* (K)**	150
**μ (mm^{-1})**	0.699
***F*(000)**	1080
**θ range (°)**	2.754–77.228
**Reflections collected**	14233
**Independent reflections**	2424
**Obs. Data [$I > 2\sigma$]**	1996
**Data / restraints / parameters**	2424 / 110 / 181
**Final R_1 values ($I > 2\sigma(I)$)**	0.0618
**Final R_1 values (all data)**	0.0702
**wR_2 (all data)**	0.1971
**Goodness-of-fit on $\|F\|^2$**	1.052
**CCDC**	2290410

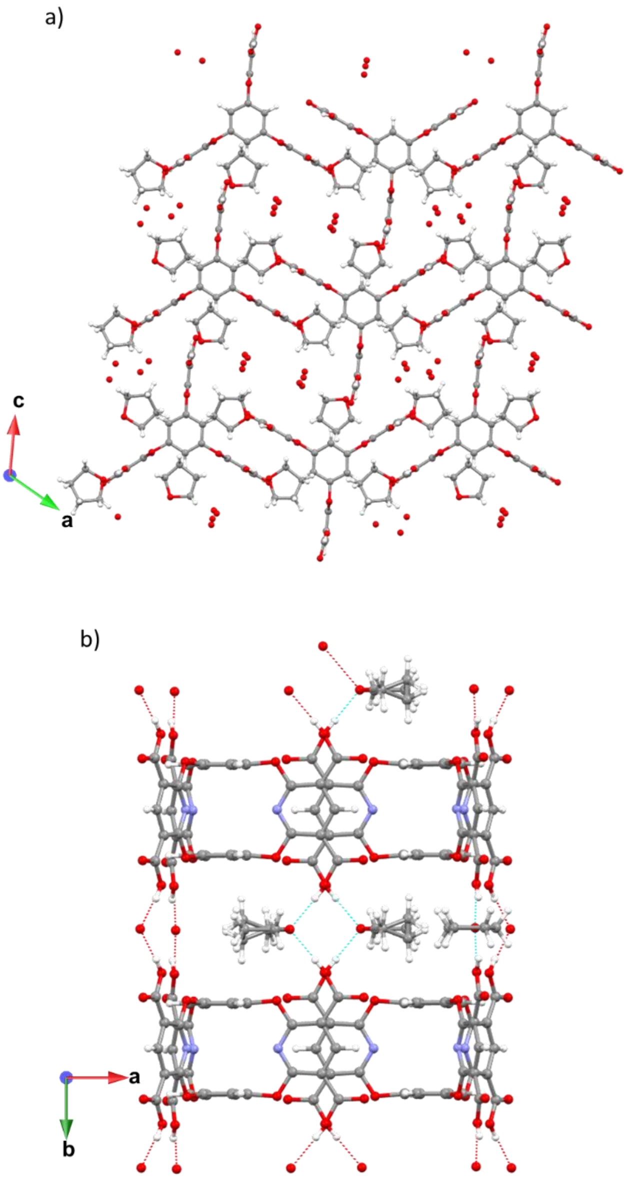

Figure S32 Illustration for the single crystal structure of **CageHOF-2·THF**. Two views are shown, top view (a), side view (b). Atoms, H = white; C = grey; N = blue; O = red.

Table S9. Single crystal refinement details for **CageHOF-2·THF**.

**Name**	**CageHOF-2·THF**
**Crystallization solvent**	THF, pentane
**Wavelength/ Å**	Mo-Kα, 0.71073
**Formula**	$C_{33} H_{15} N_3 O_{18}$, 1.5($C_8 H_{16} O_2$), 5($H_2 O$)
**Weight**	1039.15
**Crystal system**	monoclinic
**Space group**	*P*2/*m*
a **(Å)**	11.3464(18)
b **(Å)**	11.3308(16)
c **(Å)**	20.629(3)
$\alpha = \gamma$ **(°)**	90
β **(°)**	101.247(16)
V **(Å^3)**	2601.2(7)
ρ_{calcd} **(g cm^{-1})**	1.327
Z	2
T **(K)**	200
μ **(mm^{-1})**	0.111
F**(000)**	1077.0
θ **range (°)**	1.830–23.255
**Reflections collected**	6867
**Independent reflections**	6867
**Obs. Data [$I > 2\sigma$]**	2918
**Data / restraints / parameters**	6867 / 60 /378
**Final R_1 values ($I > 2\sigma(I)$)**	0.0681
**Final R_1 values (all data)**	0.1532
**wR_2 (all data)**	0.1922
**Goodness-of-fit on $\|F\|^2$**	0.840
**CCDC**	2253691

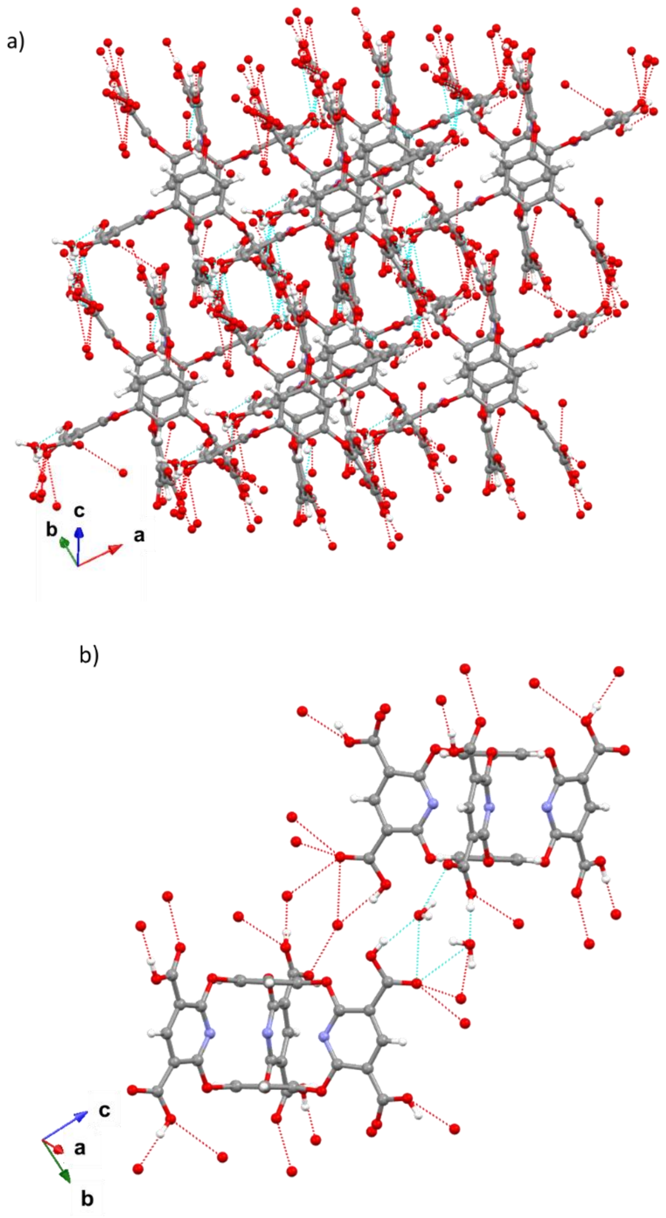

Figure S33 Illustration for the single crystal structure of **CageHOF-2·H_2O**. Two views are shown, top view (a), side view (b). Atoms, H = white; C = grey; N = blue; O = red.

Table S10. Single crystal refinement details for **CageHOF-2·H_2O**.

**Name**	**CageHOF-2·H_2O**
**Crystallization solvent**	EtOH, H_2O
**Wavelength/ Å**	Mo-Kα, 0.71073
**Formula**	C_{33} H_{15} N_3 O_{18}, 2.75(H_2 O), 0.5(O)
**Weight**	800.03
**Crystal system**	triclinic
**Space group**	$P\bar{1}$
a **(Å)**	10.9801(6)
b **(Å)**	12.3246(8)
c **(Å)**	12.4321(8)
α **(°)**	89.298(5)
β (°)	80.180(5)
γ **(°)**	80.363(5)
V **($Å^3$)**	1634.10(18)
**ρ_{calcd} (g cm^{-1})**	1.626
Z	2
T **(K)**	200
**μ (mm^{-1})**	0.140
F**(000)**	821.0
θ **range (°)**	2.308–23.420
**Reflections collected**	17011
**Independent reflections**	4687
**Obs. Data [I > 2σ]**	3024
**Data / restraints / parameters**	4687 / 2 / 539
**Final R_1 values (I > 2σ(I))**	0.0535
**Final R_1 values (all data)**	0.1009
**wR_2 (all data)**	0.1313
**Goodness-of-fit on $\|F\|^2$**	1.004
**CCDC**	2253690

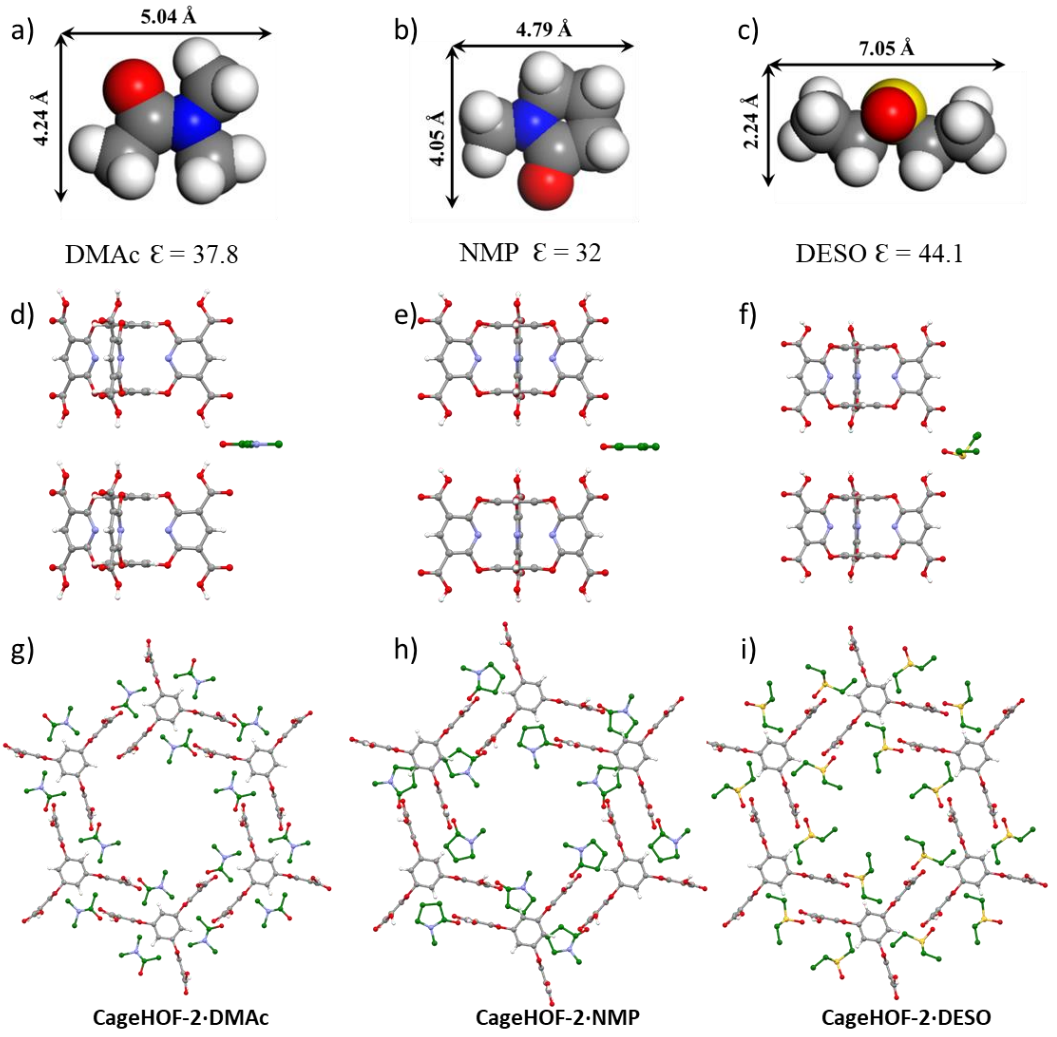

Figure S34 A comparison of the molecular sizes of the organic solvents NMP, DMAc, DESO and their dielectric constant, alongside the isostructural experimental SCXRD structures. In each of the solvates, the O atom of the solvent hydrogen bonds to one of the carboxylic acid groups of **Cage-6-COOH**, influencing its crystal packing.

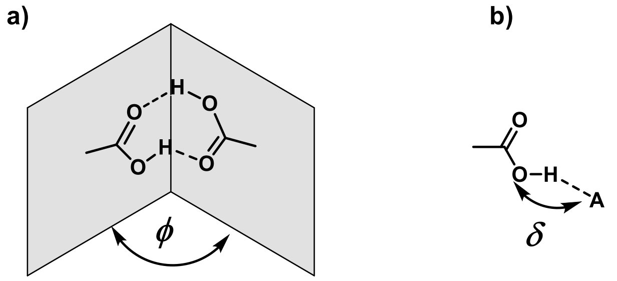

Figure S35 Illustration for (a) the dihedral angles measurement of carboxylic dimers, and (b) the H-bond angle between the carboxylic acid groups and the H-bond acceptors.

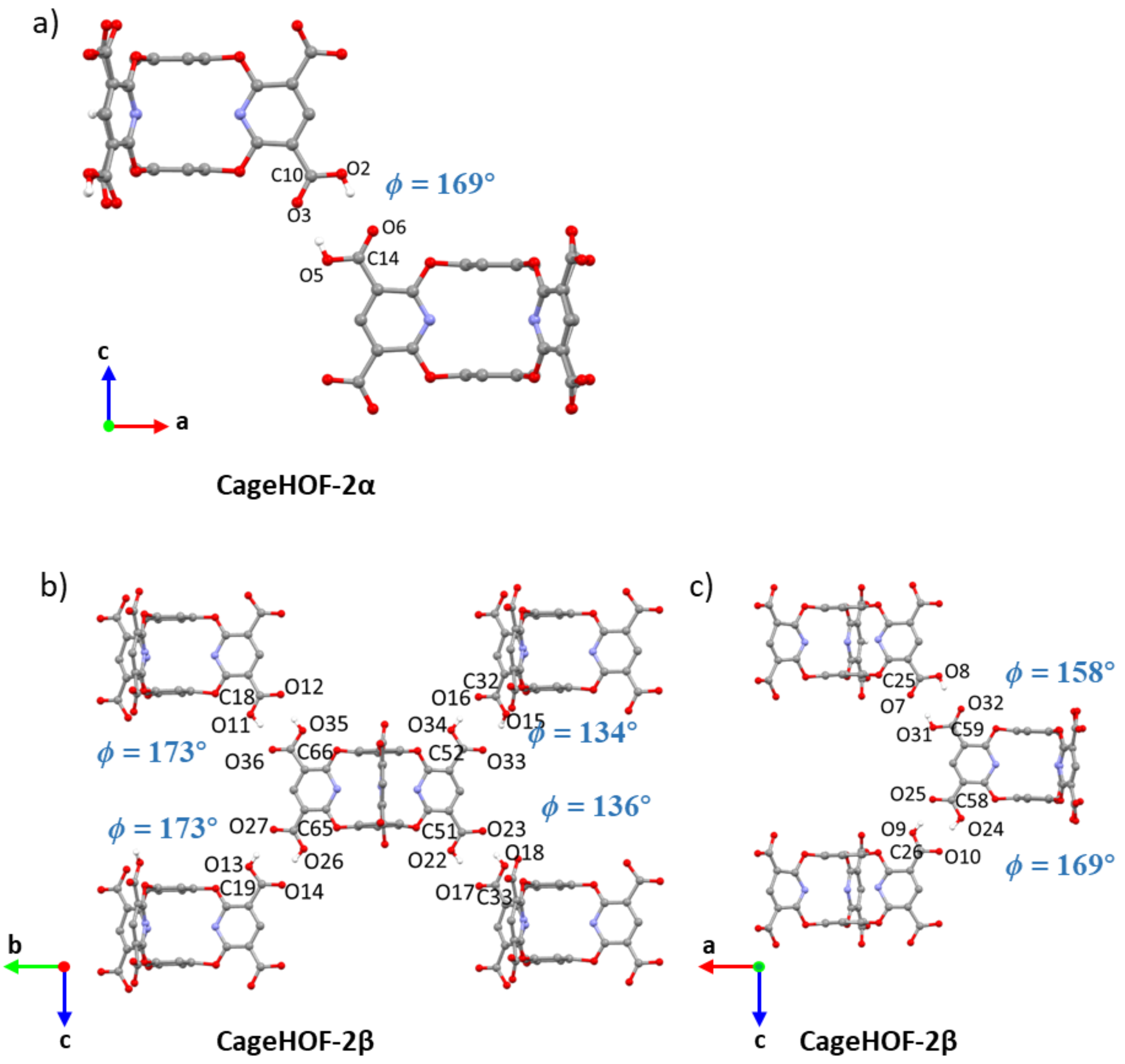

Figure S36 Hydrogen bonding interactions in the SCXRD structures (a) **CageHOF-2α**, (b) **CageHOF-2β**. The atoms involved in the hydrogen-bonding interactions are labelled and the distances are listed in Table S12.

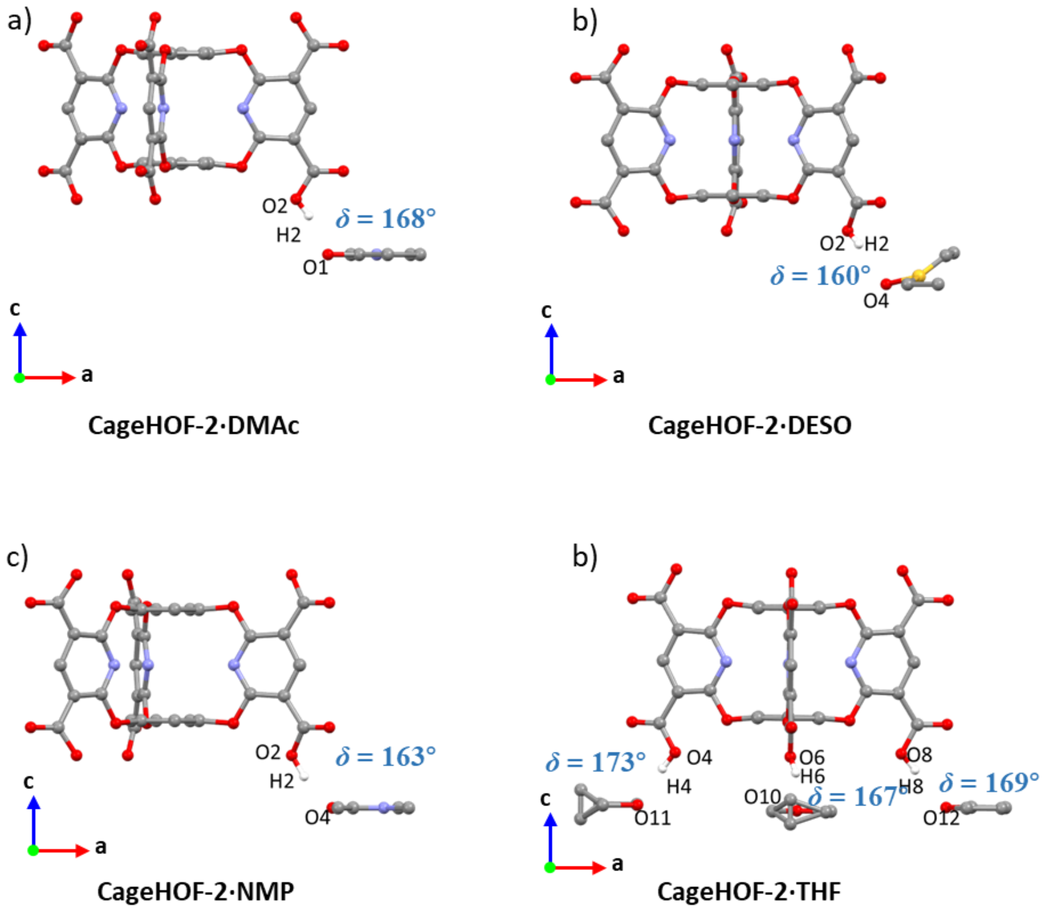

Figure S37 Hydrogen bonding interactions in the SCXRD structures (a) **CageHOF-2·DMAc**, (b) **CageHOF-2·DESO**, (c) **CageHOF-2·NMP**, (d) **CageHOF-2·THF**. The atoms involved in the hydrogen-bonding interactions are labelled and the distances are listed in Table S13.

Table S11. Summary of the dihedral angle information of **Cage-6-COOH** in the seven SCXRD structures reported in this study.

**SCXRD Structure**	$\boldsymbol{\Phi_{min}}$ **(°)**	$\boldsymbol{\Phi}$ **(°)**	$\boldsymbol{\Phi_{max}}$ **(°)**
**CageHOF-2α**	116	118	126
**CageHOF-2β**[a]	113	113	134
	90	127	143
**CageHOF-2·DMAc**	120	120	120
**CageHOF-2·DESO**	120	120	120
**CageHOF-2·NMP**	120	120	120
**CageHOF-2·THF**	114	117	129
**CageHOF-2·H_2O**	99	104	157

[a] The are two **Cage-6-COOH** molecules in the asymmetric units in **CageHOF-2β**.

Table S12 Dihedral angles of carboxylic acid dimers from the SCXRD structure of HOFs reported in this study.

SCXRD Structure	Plane 1	Plane 2	Dihedral Angle (δ, °)
**CageHOF-2α**	O2-C10-O3	O5-C14-O6	169
**CageHOF-2β**	O11-C18-O12	O35-C66-O36	173
	O13-C19-O14	O26-C65-O27	173
	O15-C32-O16	O33-C52-O34	134
	O17-C33-O18	O22-C51-O23	136
	O7-C25-O8	O31-C59-O32	158
	O9-C26-O10	O24-C58-O25	169

Table S13 H-bond interaction information from the SCXRD structure of solvate HOFs reported in this study.

SCXRD Structure	D–H–A Atom Labels	DH (Å)	HA (Å)	DA (Å)	Angle (δ, °)
**CageHOF-2·DMAc**	O2-H2-O4	0.96	1.67	2.62	168
**CageHOF-2·DESO**	O2-H2-O4	0.75	1.91	2.62	159
**CageHOF-2·NMP**	O2-H2-O1	0.82	1.83	2.62	162.7
**CageHOF-2·THF**	O4-H4-O11	0.84	1.87	2.70	173.1
	O6-H6-O10	0.84	1.89	2.71	166.6
	O8-H8-O12	0.84	1.91	2.74	169.2

Table S14 Summary of the distances and overlap between the 1,3,5-subsituted aromatic rings in the seven SCXRD structure recorded during this study. The graphic below the table shows how we calculated the distances and angles for each structure.

**SCXRD structure**	***d*** **(Å)**[a]	***δ*** **(°)**[a]
**CageHOF-2α**	4.41	90.5
**CageHOF-2β**	4.40	89.1
**CageHOF-2·DMAc**	4.36	90.1
**CageHOF-2·DESO**	4.35	90.8
**CageHOF-2·NMP**	4.32	90.1
**CageHOF-2·THF**	4.31	90.0
**CageHOF-2·H_2O**[c]	4.38	90.1

[a] see graphic, below, used to measure the distance (*d*) and angle (*δ*) between the two 1,3,5-sustituted aromatic ring.

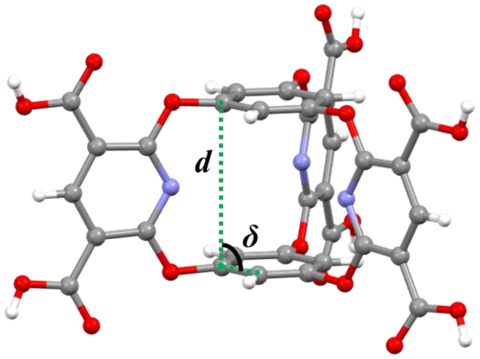

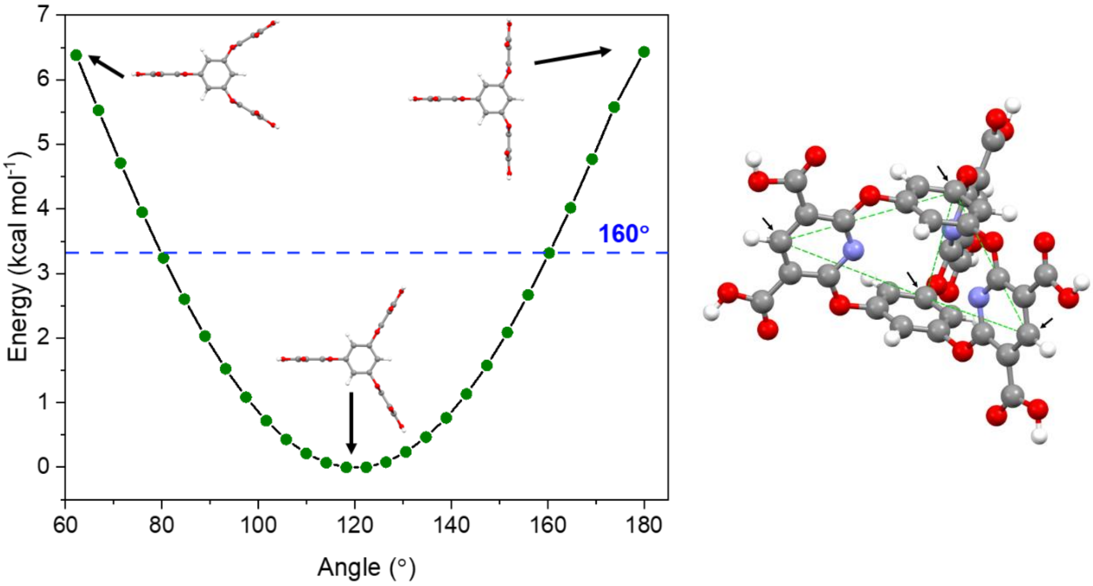

Figure S38 Conformational energies from relaxed scan DFT calculations that we used to investigate the flexibility of **Cage-6-COOH**. We performed these DFT calculations by scanning through the dihedral angle between the green dashed triangles shown in the righthand image. We then calculated the dihedral angles using the planes of the aromatic rings, as we used for the experimental structures, to generate the lefthand plot. These DFT calculations revealed that the lowest energy conformer had dihedral angles between the aromatic pillars of around 120°. Plotting the relative potential energy variation vs the dihedral angle from relaxed scan DFT calculation revealed that the energy required to rotate the aromatic pillars ± 60° was 6.43 kcal mol^{-1}.

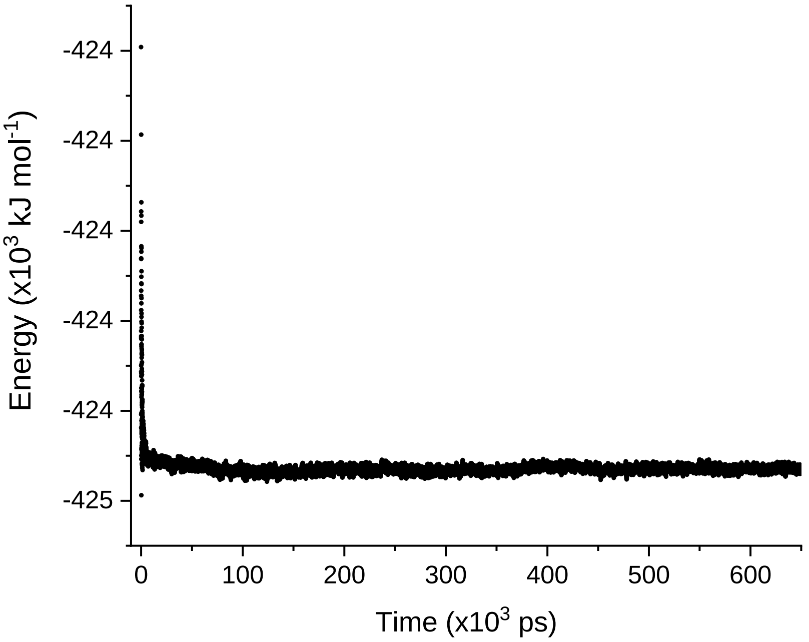

Figure S39 Plot of the system energy changes during the molecular dynamic simulations.

Section 8 Structural flexibility investigation

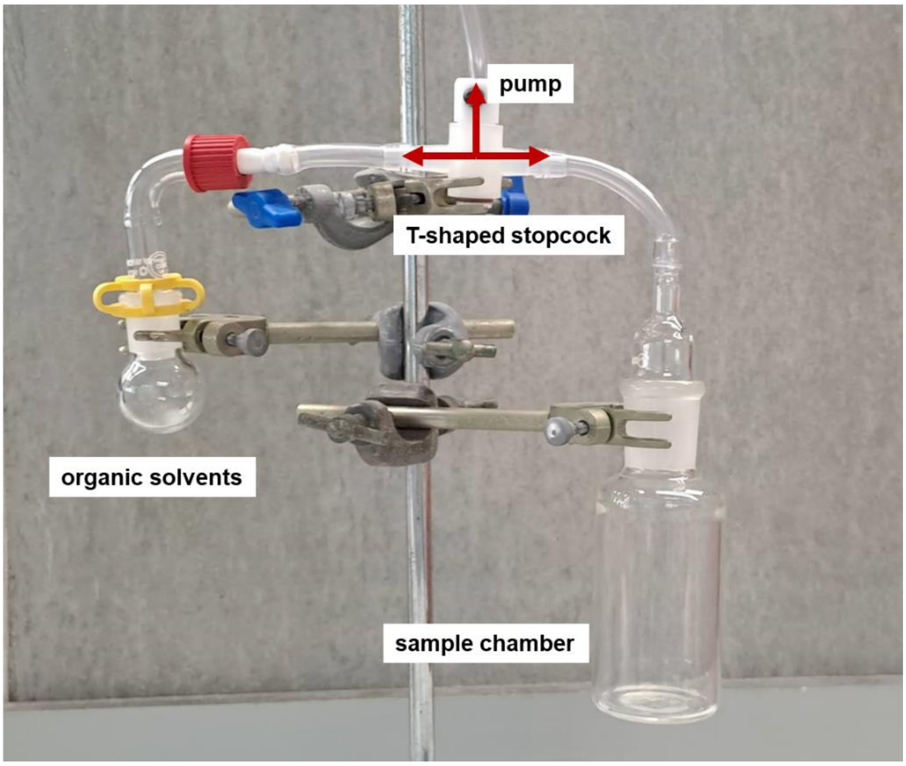

Figure S40 Equipment used to investigate the dynamic behaviour of HOFs under various solvents. The sample was placed in the chamber and then evacuated through the pump at room temperature for 3 h. Afterwards, the chamber was purged with organic vapor and then left for equilibrium for 1 h. Finally, the sample was added to a capillary for PXRD analysis.

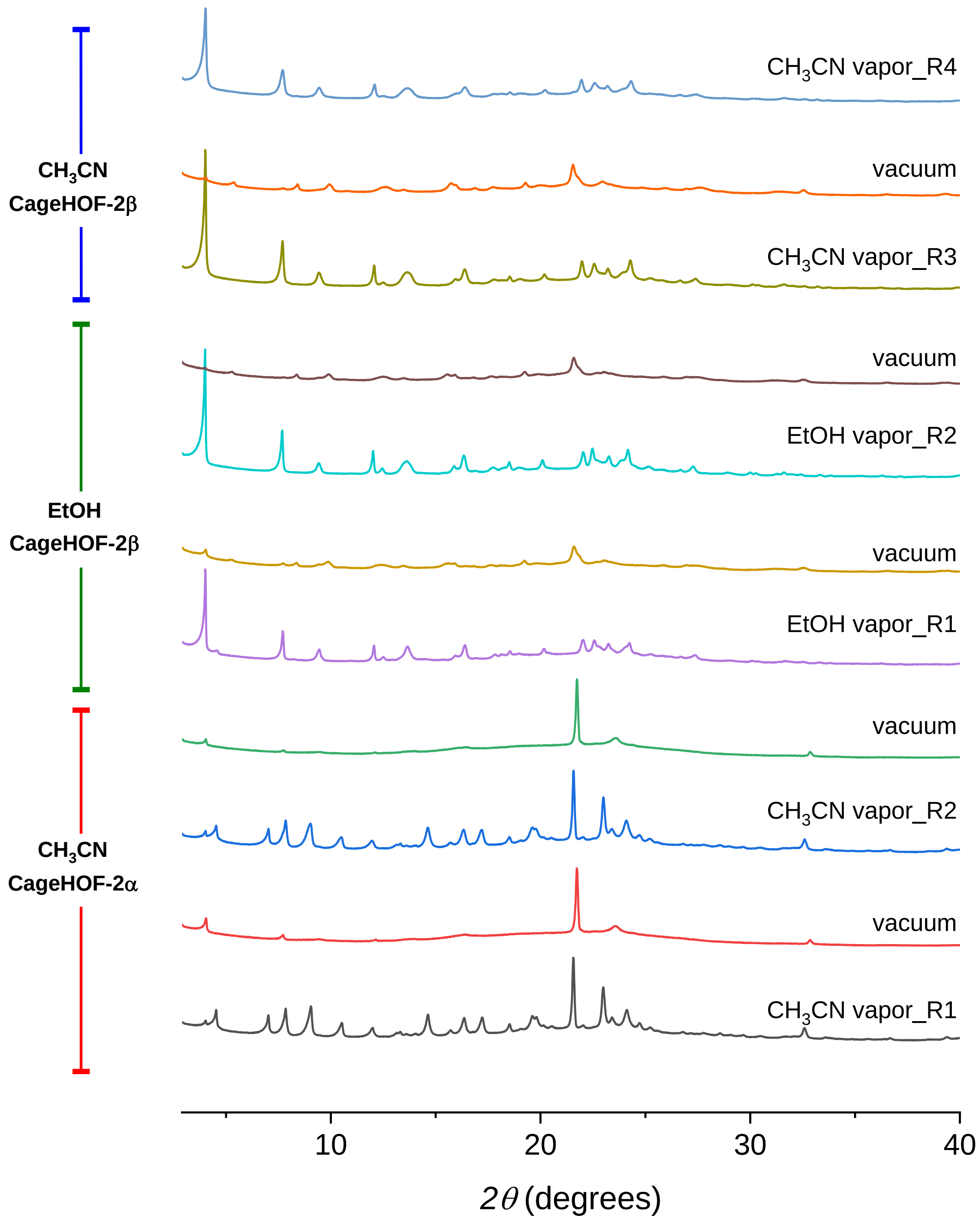

Figure S41 Phase transformation of **CageHOF-2α** upon exposure to CH_3CN vapor or EtOH vapor. The stepwise PXRD patterns were collected after each step using the capillary model. The activated **CageHOF-2α** was used as the starting material. First, each sorption process was conducted for 1 h. Then, the sample was degassed for 3 h at room temperature under dynamic vacuum conditions.

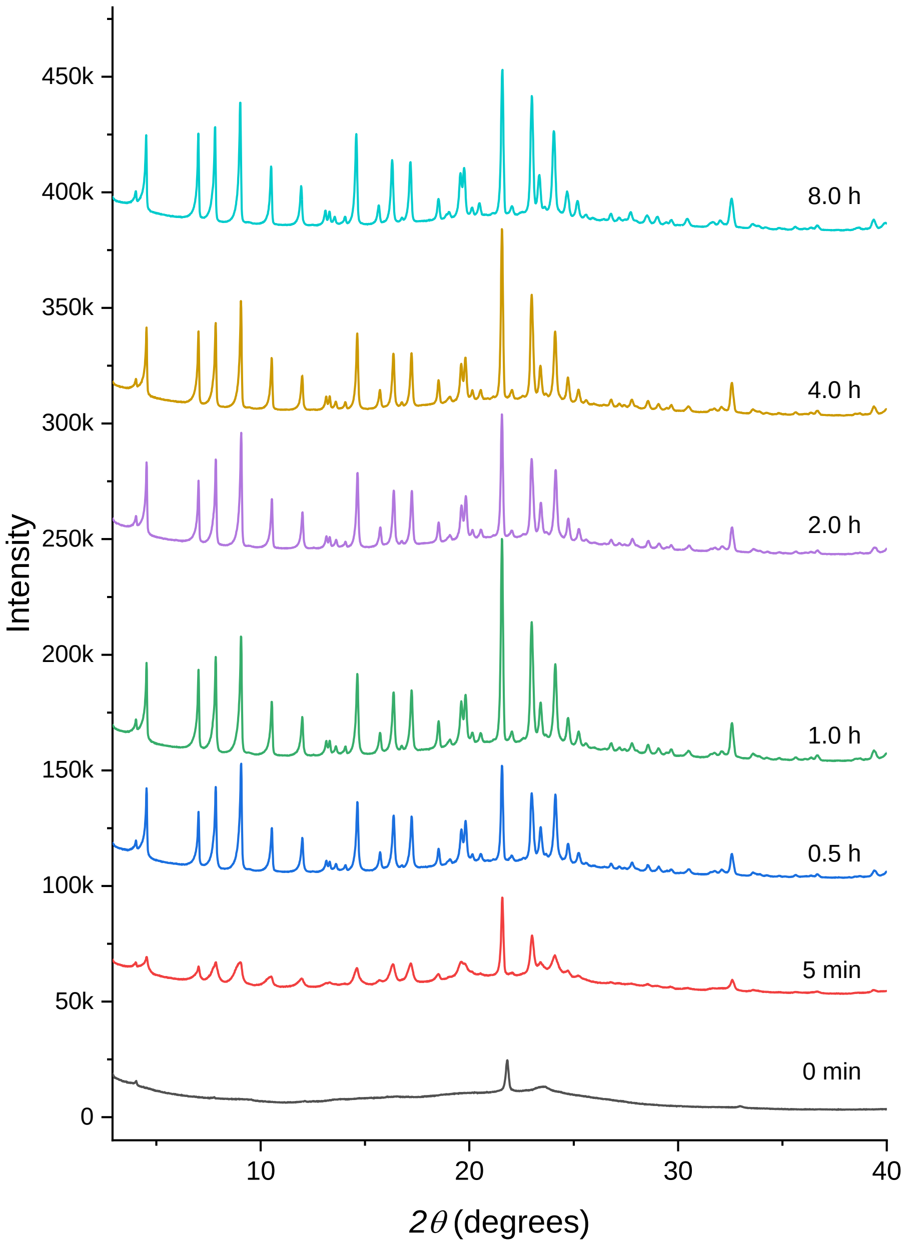

Figure S42 Time-dependent study of the self-healing behaviour of scCO_2-activated **CageHOF-2α** in the presence of CH_3CN. The scCO_2-activated **CageHOF-2α** and 0.5 mL CH_3CN were added to a vial, which was sealed and left without being disturbed at room temperature for various times. After exposure to CH_3CN, the PXRD patterns were recorded in a capillary.

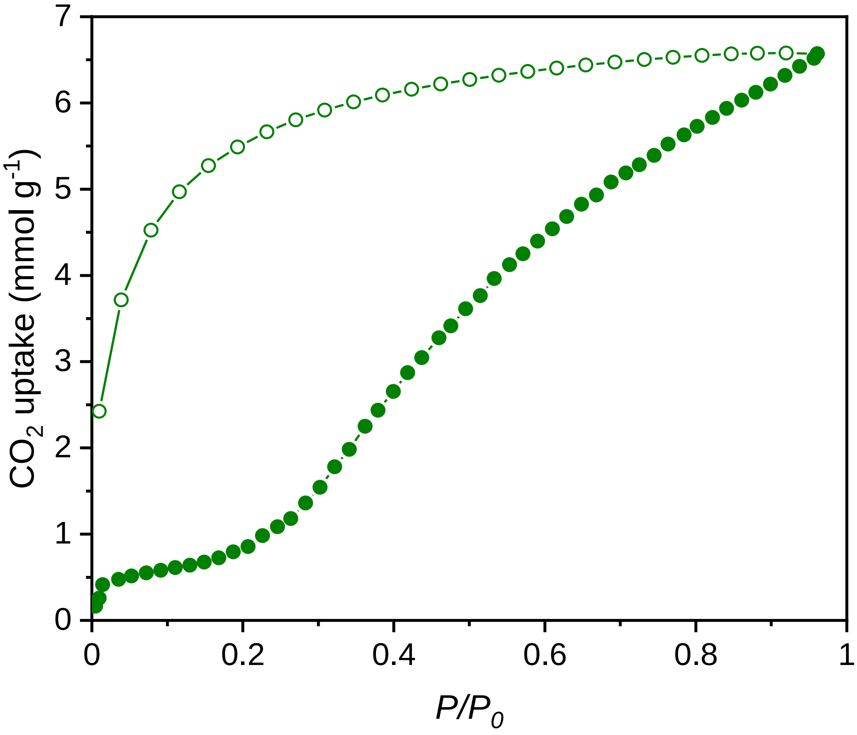

Figure S43 CO_2 sorption isotherms of scCO_2-activated **CageHOF-2α** at 195 K. An open-gate behaviour was observed at P/P_0 = 0.21, where a pore volume was calculated as 0.024 cm^3 g^{-1} and changed to 0.185 cm^3 g^{-1} at P/P_0 = 0.96.

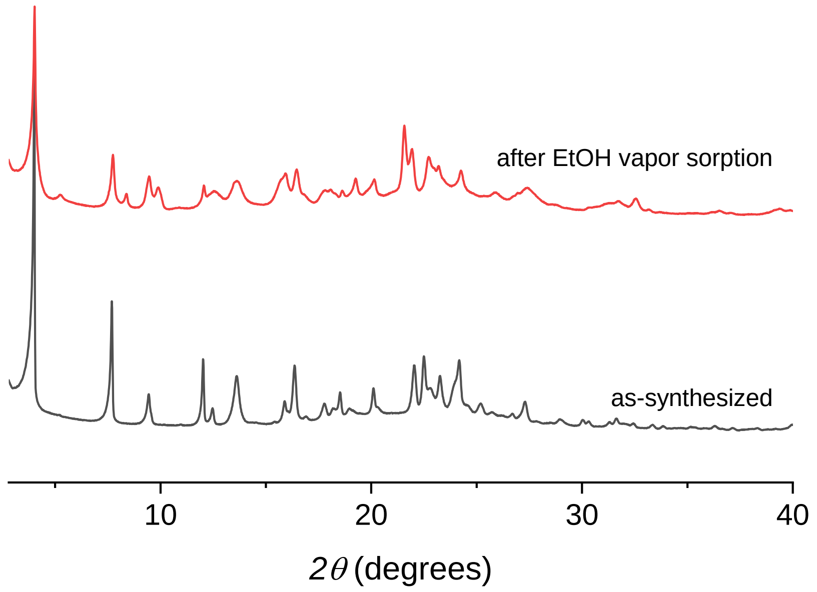

Figure S44 PXRD profiles recorded after the EtOH vapor sorption experiment.

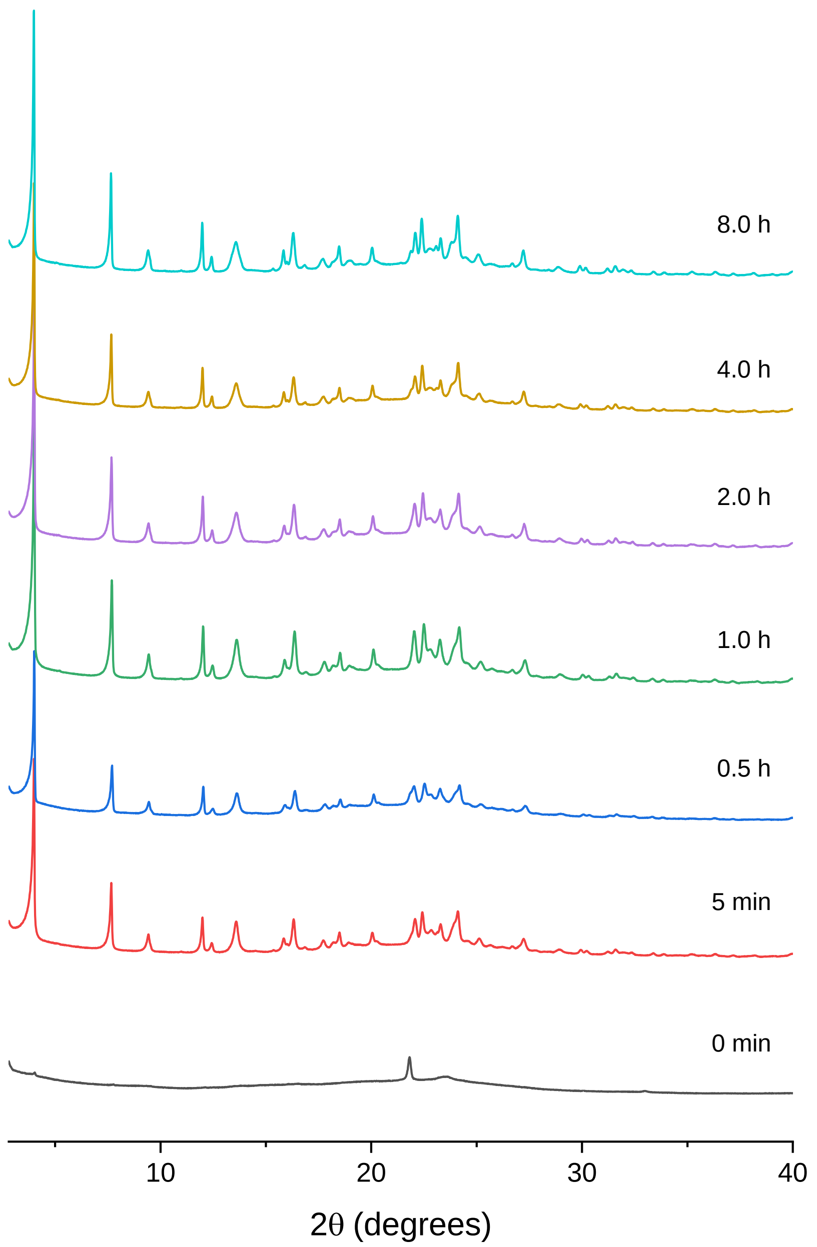

Figure S45 EtOH vapor adsorption dynamics study of $scCO_2$-activated **CageHOF-2α**. The $scCO_2$-activated powder and 0.5 mL EtOH were added to a vial, which was sealed and left without being disturbed at room temperature for various times. After exposure to EtOH vapor, the PXRD patterns were recorded in a capillary.

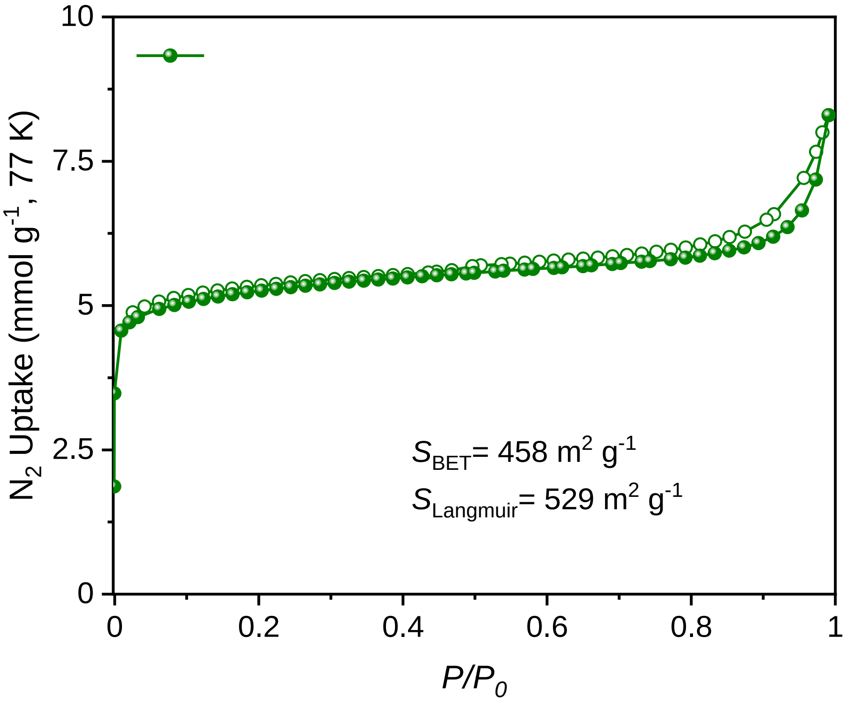

Figure S46 N_2 sorption Isotherms of **CageHOF-2β** powder at 77 K, which was prepared by the phase transformation from **CageHOF-2α**. The sample was exchanged with acetone and pentane (ten times) before degassing at 30 °C under dynamic vacuum conditions.

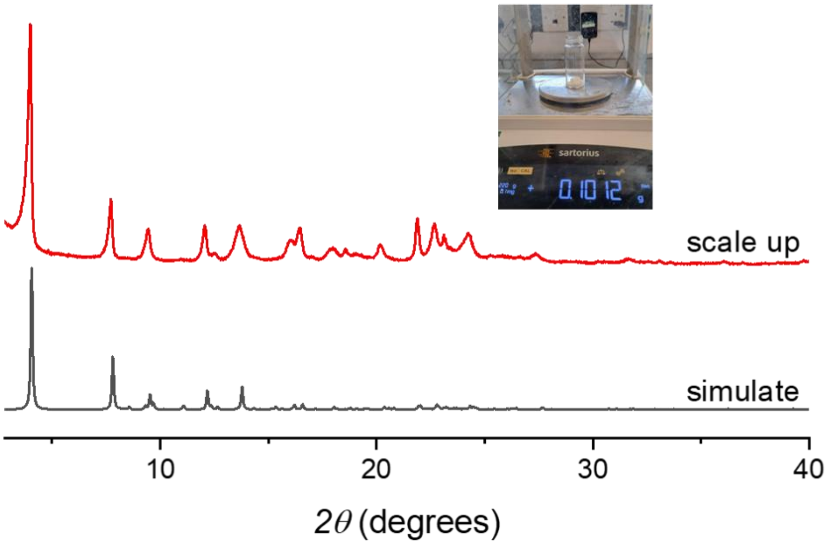

Figure S47 Comparison between the scale-up PXRD pattern of **CageHOF-2β** and simulated PXRD patten of the SCXRD structure. For this measurement, we used 101 mg of sample.

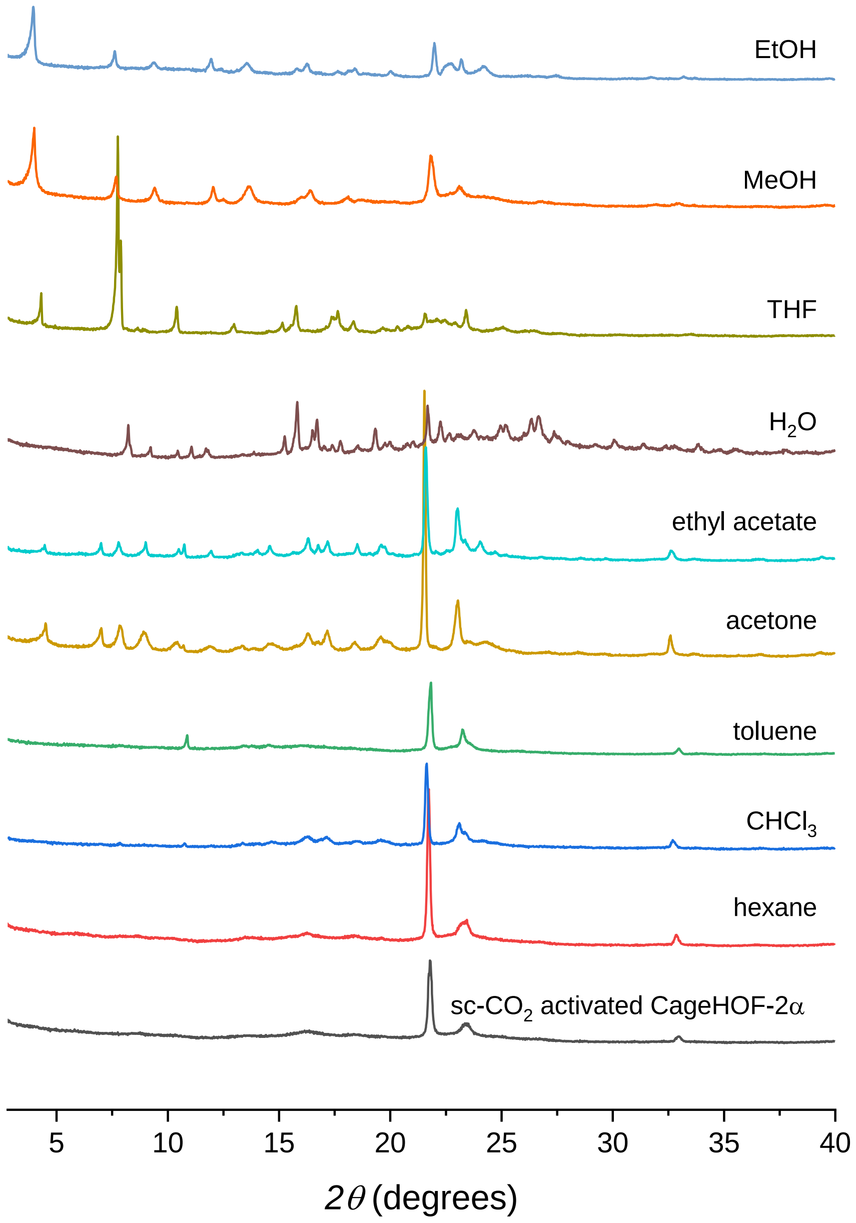

Figure S48 PXRD patterns recorded after exposing activated **CageHOF-2** powder to 10 µL of the different solvents listed in the figure. The activated **CageHOF-2** powder for these measurements was prepared from **CageHOF-2α** after activation with $scCO_2$.

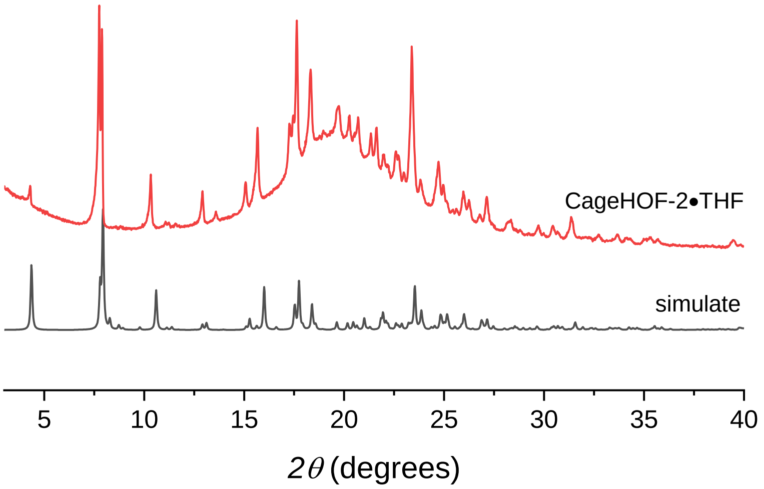

Figure S49 Comparison between the experimental PXRD pattern of **CageHOF-2α·THF** that was transformed from activated **CageHOF-2** powder in response to THF and the simulated PXRD pattern based on the SCXRD structure. We used the activated **CageHOF-2** powder as the starting material for this measurement.

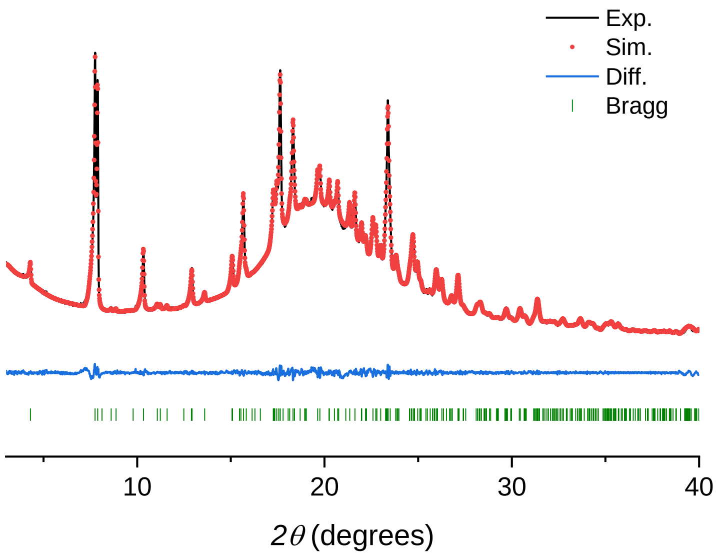

Figure S50 Pawley fitting of **CageHOF-2·THF** powder (*P*2/*m*, a = 11.47 Å, b =11.40 Å, c = 21.04 Å, $\alpha = \gamma = 90°$, $\beta = 102.6°$, V = 2683.6 Å^3, R_{wp} = 1.75% and R_p = 1.30%), which confirms that the structure of **CageHOF-2·THF** powder is comparable to the SCXRD structure of **CageHOF-2·THF** (Table S9). Black line: solvated experimental PXRD pattern, red points: fitting pattern, blue curve: the difference between experimental and refinement, green bars: reflection positions.

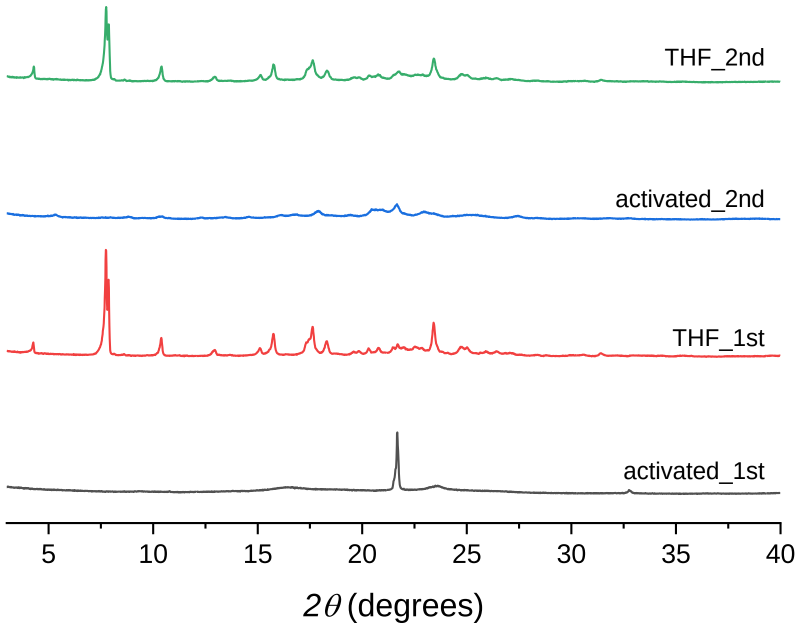

Figure S51 Phase transformation of activated **CageHOF-2** powder upon wetting with THF (20 µL).

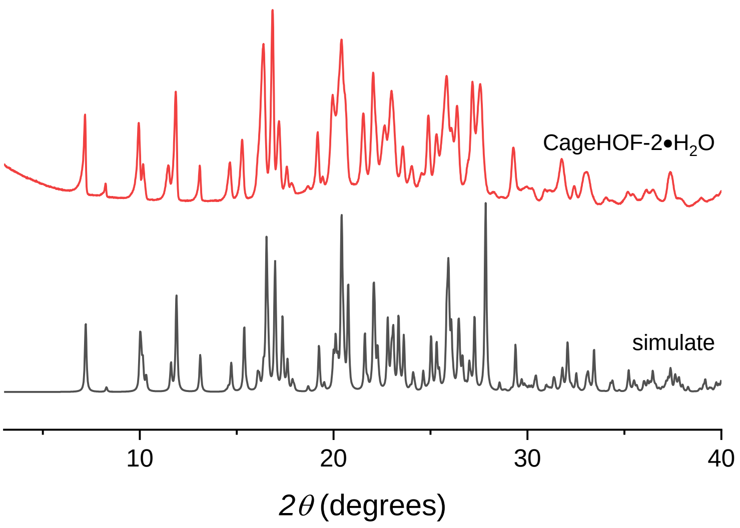

Figure S52 Comparison between the experimental PXRD pattern of **CageHOF-2α·H₂O** that was obtained by the solvent exchange of **CageHOF-2α** with H_2O shown above the simulated PXRD pattern based on the SCXRD structure **CageHOF-2α·H₂O**.

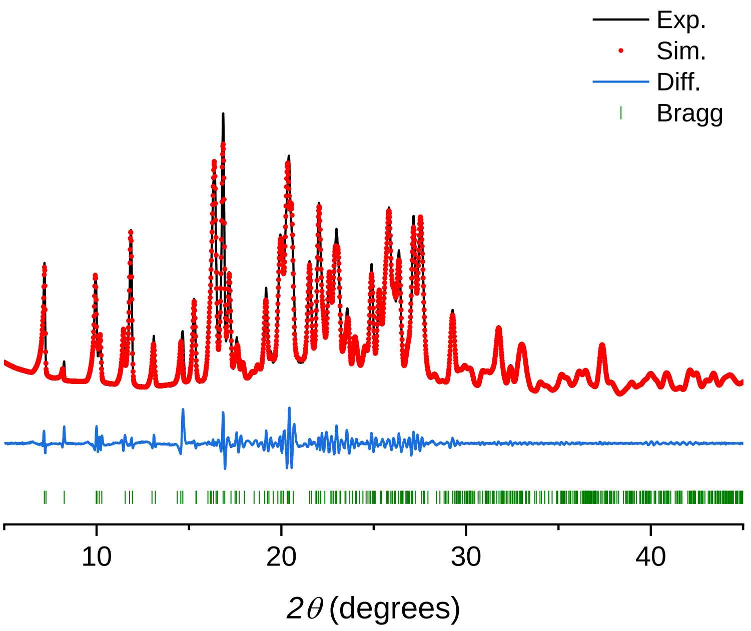

Figure S53 Pawley fitting of **CageHOF-2·H₂O** powder ($P\bar{1}$, a = 11.03 Å, b = 12.36 Å, c = 12.49 Å, α = 89.09°, β = 80.66°, γ = 79.69°, V = 1652 Å^3, R_{wp} = 5.12%, R_p = 3.27%), which confirms that the **CageHOF-2·H₂O** powder is comparable to the SCXRD structure of **CageHOF-2·H₂O** (Table S10). Black line: solvated experimental PXRD pattern, red points: fitting pattern, blue curve: the difference between experimental and refinement, green bars: reflection positions.

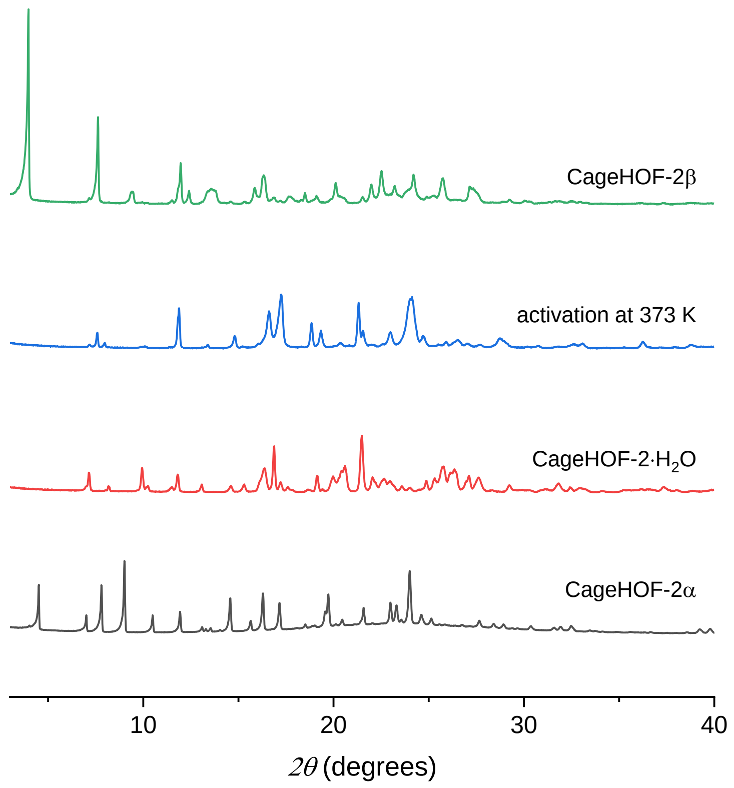

Figure S54 Phase transformation of **CageHOF-2·H_2O** powder after activation under dynamic vacuum at 373 K or wetting with EtOH (20 μL). **CageHOF-2·H_2O** powder exhibited a reversible phase switch upon the treatment by activating under a dynamic vacuum at 373 K for 1 h or wetting with H_2O.

Reference

(1) Sheldrick, G. SHELXT. *Acta Cryst.* **2015**, *A71* (A71), 3–8.

(2) Sheldrick, G. SHELXL. *Acta Cryst.* **2015**, *C71* (C71), 3–8.

(3) O. V. Dolomanov R. J. Gildea, J. A. K. Howard and H. Puschmann, L. J. B. OLEX2. *J. Appl. Cryst.* **2009**, *42* (42), 339–341.

(4) Frisch, M. J.; Trucks, G. W.; Schlegel, H. B.; Scuseria, G. E.; Robb, M. A.; Cheeseman, J. R.; Scalmani, G.; Barone, V.; Petersson, G. A.; Nakatsuji, H. Gaussian 16 Revision a. 03. 2016; Gaussian Inc. *Wallingford CT* **2016**, *2* (3), 4.

(5) Zhao, Y.; Truhlar, D. G. The M06 Suite of Density Functionals for Main Group Thermochemistry, Thermochemical Kinetics, Noncovalent Interactions, Excited States, and Transition Elements: Two New Functionals and Systematic Testing of Four M06-Class Functionals and 12 Other Function. *Theor. Chem. Acc.* **2008**, *120* (1–3), 215–241.

(6) Weigend, F.; Ahlrichs, R. Balanced Basis Sets of Split Valence, Triple Zeta Valence and Quadruple Zeta Valence Quality for H to Rn: Design and Assessment of Accuracy. *Phys. Chem. Chem. Phys.* **2005**, *7* (18), 3297–3305.

(7) Weigend, F. Accurate Coulomb-Fitting Basis Sets for H to Rn. *Phys. Chem. Chem. Phys.* **2006**, *8* (9), 1057–1065.

(8) Humphrey, W.; Dalke, A.; Schulten, K. VMD: Visual Molecular Dynamics. *J. Mol. Graph.* **1996**, *14* (1), 33–38.

(9) Dubbeldam, D.; Calero, S.; Ellis, D. E.; Snurr, R. Q. RASPA: Molecular Simulation Software for Adsorption and Diffusion in Flexible Nanoporous Materials. *Mol. Simul.* **2016**, *42* (2).

(10) Rizzo, R. C.; Jorgensen, W. L. OPLS All-Atom Model for Amines: Resolution of the Amine Hydration Problem. *J. Am. Chem. Soc.* **1999**, *121* (20).

(11) Kaminski, G. A.; Friesner, R. A.; Tirado-Rives, J.; Jorgensen, W. L. Evaluation and Reparametrization of the OPLS-AA Force Field for Proteins via Comparison with Accurate Quantum Chemical Calculations on Peptides. *J. Phys. Chem. B* **2001**, *105* (28).

(12) Robertson, M. J.; Tirado-Rives, J.; Jorgensen, W. L. Improved Peptide and Protein Torsional Energetics with the OPLS-AA Force Field. *J. Chem. Theory Comput.* **2015**, *11* (7).

(13) Melchionna, S.; Ciccotti, G.; Holian, B. L. Hoover Npt Dynamics for Systems

Varying in Shape and Size. *Mol. Phys.* **1993**, *78* (3).

(14) Mastalerz, M.; Oppel, I. M. Rational Construction of an Extrinsic Porous Molecular Crystal with an Extraordinary High Specific Surface Area. *Angew. Chem. Int. Ed.* **2012**, *51* (21), 5252–5255.

(15) Pulido, A.; Chen, L.; Kaczorowski, T.; Holden, D.; Little, M. A.; Chong, S. Y.; Slater, B. J.; McMahon, D. P.; Bonillo, B.; Stackhouse, C. J.; Stephenson, A.; Kane, C. M.; Clowes, R.; Hasell, T.; Cooper, A. I.; Day, G. M. Functional Materials Discovery Using Energy-Structure-Function Maps. *Nature* **2017**, *543* (7647), 657–664.

(16) Yan, W.; Yu, X.; Yan, T.; Wu, D.; Ning, E.; Qi, Y.; Han, Y. F.; Li, Q. A Triptycene-Based Porous Hydrogen-Bonded Organic Framework for Guest Incorporation with Tailored Fitting. *Chem. Commun.* **2017**, *53* (26), 3677–3680.

(17) Li, P.; Li, P.; Ryder, M. R.; Liu, Z.; Stern, C. L.; Farha, O. K.; Stoddart, J. F. Interpenetration Isomerism in Triptycene-Based Hydrogen-Bonded Organic Frameworks. *Angew. Chem. Int. Ed.* **2019**, *58* (6), 1664–1669.

(18) Li, P.; Chen, Z.; Ryder, M. R.; Stern, C. L.; Guo, Q. H.; Wang, X.; Farha, O. K.; Stoddart, J. F. Assembly of a Porous Supramolecular Polyknot from Rigid Trigonal Prismatic Building Blocks. *J. Am. Chem. Soc.* **2019**, *141* (33), 12998–13002.

(19) Zhou, Y.; Kan, L.; Eubank, J. F.; Li, G.; Zhang, L.; Liu, Y. Self-Assembly of Two Robust 3D Supramolecular Organic Frameworks from a Geometrically Non-Planar Molecule for High Gas Selectivity Performance. *Chem. Sci.* **2019**, *10* (26), 6565–6571.

(20) Zhang, X.; Wang, J. X.; Li, L.; Pei, J.; Krishna, R.; Wu, H.; Zhou, W.; Qian, G.; Chen, B.; Li, B. A Rod-Packing Hydrogen-Bonded Organic Framework with Suitable Pore Confinement for Benchmark Ethane/Ethylene Separation. *Angew. Chem. Int. Ed.* **2021**, *60* (18), 10304–10310.

(21) Yamagishi, H.; Sato, H.; Hori, A.; Sato, Y.; Matsuda, R.; Kato, K.; Aida, T. Self-Assembly of Lattices with High Structural Complexity from a Geometrically Simple Molecule. *Science (80-.).* **2018**, *361* (6408), 1242–1246.

(22) Zhu, Q.; Johal, J.; Widdowson, D. E.; Pang, Z.; Li, B.; Kane, C. M.; Kurlin, V.; Day, G. M.; Little, M. A.; Cooper, A. I. Analogy Powered by Prediction and Structural Invariants: Computationally Led Discovery of a Mesoporous Hydrogen-Bonded Organic Cage Crystal. *J. Am. Chem. Soc.* **2022**, *144* (22),

9893–9901.

(23) Han, B.; Wang, H.; Wang, C.; Wu, H.; Zhou, W.; Chen, B.; Jiang, J. Postsynthetic Metalation of a Robust Hydrogen-Bonded Organic Framework for Heterogeneous Catalysis. *J. Am. Chem. Soc.* **2019**, *141* (22), 8737–8740.

(24) Graffner-Nordberg, M.; Kolmodin, K.; Åqvist, J.; Queener, S. F.; Hallberg, A. Design, Synthesis, Computational Prediction, and Biological Evaluation of Ester Soft Drugs as Inhibitors of Dihydrofolate Reductase from *Pneumocystis carinii*. *J. Med. Chem.* **2001**, *44* (15), 2391–2402

(25) Katz, J. L.; Selby, K. J.; Conry, R. R. Single-Step Synthesis of $D3_h$-Symmetric Bicyclooxacalixarenes. *Org. Lett.* **2005**, *7* (16), 3505–3507.

(26) Tyler, A. R.; Ragbirsingh, R.; McMonagle, C. J.; Waddell, P. G.; Heaps, S. E.; Steed, J. W.; Thaw, P.; Hall M. J.; Probert, M. R. Encapsulated Nanodroplet Crystallization of Organic-Soluble Small Molecules. *Chem*, **2020**, *6* (7), 1755–1765.
